# A Multi-Strategy Improved Seagull Optimization Algorithm for Global Optimization and Artistic Image Segmentation

**DOI:** 10.3390/biomimetics11040247

**Published:** 2026-04-03

**Authors:** Yangyang Jiang

**Affiliations:** 1 Academy of Fine Arts, Chongqing University of Education, Chongqing 400067, China; jiangyy@cque.edu.cn; 2 Department of Design Innovation, Sejong University, Seoul 05006, Republic of Korea

**Keywords:** seagull optimization algorithm, image segmentation, multi-strategy improved, numerical optimization

## Abstract

Multilevel threshold image segmentation is a key task in image processing, yet it faces challenges such as low search efficiency in high-dimensional spaces, difficulty in balancing segmentation accuracy and stability, and insufficient adaptability to complex scenes. Existing solutions mainly include traditional thresholding methods and metaheuristic optimization-based schemes, but they still face limitations in high-dimensional and complex segmentation tasks. The standard Seagull Optimization Algorithm (SOA) suffers from shortcomings including a single exploration mechanism, weak local exploitation capability, and a tendency for population diversity to deteriorate, making it difficult to meet the demands of high-dimensional optimization. To address these issues, this paper proposes a multi-strategy fused improved Seagull Optimization Algorithm (MFISOA), which integrates three strategies: adaptive cooperative foraging, differential evolution-driven exploitation, and centroid opposition-based boundary control. These strategies jointly construct a collaborative optimization framework with dynamic resource allocation, fine local search, and population diversity maintenance, thereby improving global exploration efficiency, local exploitation accuracy, and population stability. To evaluate the optimization performance of MFISOA, numerical simulation experiments were conducted on the CEC2017 and CEC2022 benchmark test suites, and comparisons were made with nine other mainstream advanced algorithms. The results show that MFISOA outperforms the competing algorithms in terms of optimization accuracy, convergence speed, and operational stability. Its superiority is further verified by the Wilcoxon rank-sum test and the Friedman test, with statistical significance (*p* < 0.05). In the multilevel threshold image segmentation task, using the Otsu criterion as the objective function, MFISOA was tested on nine benchmark images under 4-, 6-, 8-, and 10-threshold segmentation scenarios. The results indicate that MFISOA achieves better performance on metrics such as Structural Similarity Index (SSIM), Peak Signal-to-Noise Ratio (PSNR), and Feature Similarity Index (FSIM), enabling more accurate characterization of image grayscale distribution features and producing higher-quality segmentation results. This study provides an efficient and reliable approach for numerical optimization and multilevel threshold image segmentation.

## 1. Introduction

With the rapid development of image acquisition and analysis technologies, image segmentation has become a fundamental topic in computer vision and image processing, with important applications in medical diagnosis, remote sensing interpretation, industrial inspection, and environmental monitoring [1,2,3,4,5]. In particular, accurate image segmentation can support lesion localization, ecological monitoring, land-use planning, and quality control [6,7]. However, traditional segmentation methods often suffer from low efficiency and limited accuracy when dealing with high-resolution images with complex textures, which restricts their applicability in practical scenarios.

As a core image processing technique, threshold-based segmentation aims to separate meaningful regions from the background and provide a basis for subsequent image analysis, feature extraction, and decision-making [1,8,9,10,11,12]. Among existing thresholding methods, multilevel threshold segmentation has attracted increasing attention because it can describe complex image structures more precisely than single-threshold methods [13]. Nevertheless, as the number of thresholds increases, the search space expands exponentially, making exhaustive search infeasible and turning multilevel threshold selection into a high-dimensional nonlinear optimization problem [14,15,16,17]. In practical applications, this problem is further aggravated by noise interference, grayscale overlap, and the limited adaptability of many existing algorithms to different image types and complex scenes [18,19].

Intelligent optimization algorithms, also known as metaheuristic algorithms, usually draw inspiration from biological behaviors, physical processes, or social laws in nature and perform optimization through stochastic search strategies. Because of their strong global optimization capability, broad applicability, and independence from the analytical properties of objective functions, they are well suited to solving the challenges in multilevel threshold segmentation [20,21]. Such algorithms generally follow an optimization process involving population iteration, fitness evaluation, and position updating, as well as model multilevel threshold segmentation, a global optimization task in which K thresholds are treated as optimization variables and a segmentation criterion, such as Otsu’s between-class variance, is used as the fitness function. Under this framework, the algorithms can efficiently locate the optimal threshold combination in a search space that grows exponentially with the number of thresholds [22].

Early studies mainly focused on introducing classical swarm intelligence and evolutionary algorithms into multilevel threshold image segmentation. Particle Swarm Optimization (PSO) was among the earlier methods applied to multilevel thresholding. It has a simple structure and fast convergence, but it is prone to premature convergence in high-dimensional threshold search because of the loss of population diversity [23]. Gray Wolf Optimizer (GWO) exhibits strong local exploitation capability and relatively stable search behavior, but its global exploration range is limited, which may cause key thresholds to be missed when dealing with images with multimodal grayscale distributions [24]. Whale Optimization Algorithm (WOA) provides a certain balance between global exploration and local exploitation, but its convergence speed becomes slow in the later iterations, thereby affecting its optimization efficiency in complex segmentation tasks [25]. Differential Evolution (DE) achieves high local search precision, but it is sensitive to control parameters, and inappropriate parameter settings may lead to threshold selection bias [26]. Overall, these classical methods verified the feasibility of modeling multilevel threshold selection as a global optimization problem and laid the foundation for subsequent research, but they still exhibit obvious limitations in complex and high-dimensional scenarios.

Subsequently, researchers began to introduce newly developed bio-inspired metaheuristic algorithms in an attempt to improve exploration capability and population diversity through more diversified search behaviors. The Dung Beetle Optimizer (DBO) enhances population search diversity through behavior-inspired mechanisms and has shown promising exploration ability in some multimodal optimization problems, but its stability remains insufficient in high-dimensional multilevel threshold segmentation and noisy scenarios [27]. Bird-predation-inspired optimization methods, such as BPBO, enrich position updating patterns by introducing novel predation and foraging behaviors, and they have demonstrated certain competitiveness in some test tasks; however, their local exploitation precision and convergence stability still need further improvement [28]. In addition, other nature-inspired algorithms proposed in recent years have expanded the solution strategies for multilevel threshold segmentation to some extent, but they still commonly suffer from strong task dependence and limited generalization capability in high-dimensional and complex scenarios [29]. Therefore, although these newer bio-inspired algorithms employ more flexible search mechanisms, their overall performance in high-dimensional multilevel threshold image segmentation still has considerable room for improvement.

In recent years, research attention has gradually shifted toward improved and hybrid metaheuristic methods, with the aim of overcoming the limitations of a single algorithm through mechanism enhancement or algorithm fusion. In this context, the Seagull Optimization Algorithm (SOA) [30] has attracted increasing attention because it simulates the natural behaviors of seagulls during migration, aggregation, and attack, thereby constructing a search mechanism that balances global exploration and local exploitation. Existing SOA-related studies mainly improve algorithm performance by optimizing initialization strategies, introducing local perturbation mechanisms, or combining SOA with other optimization operators [31,32]. Although these improvements enhance the convergence speed and segmentation performance of SOA to a certain extent, most of them still focus on single-stage enhancement or loosely coupled hybridization and have not yet formed a coordinated mechanism that can simultaneously balance exploration efficiency, local exploitation precision, and population diversity preservation. In general, existing SOA and its improved variants still exhibit common shortcomings in multilevel threshold segmentation, including rigid exploration strategies, insufficient local exploitation precision, and coarse boundary control. Overall, although existing methods have achieved certain improvements in convergence speed, local exploitation capability, or search diversity, most of them still struggle to simultaneously balance global exploration efficiency, local exploitation precision, and population diversity preservation in high-dimensional multilevel threshold segmentation tasks. This further highlights the necessity of constructing a collaborative multi-strategy optimization framework.

To address the deficiencies of the standard SOA in multilevel threshold image segmentation, including rigid exploration strategy, insufficient local exploitation precision, and the tendency to lose population diversity, this paper proposes a Multi-Strategy Fused Improved Seagull Optimization Algorithm (MFISOA). Rather than being a simple combination of multiple strategies, the proposed method achieves a systematic improvement in algorithmic performance through three mutually coordinated mechanisms. First, an adaptive cooperative foraging strategy based on individual quality is introduced to realize dynamic allocation of search resources and enhance exploration guidance. Second, a differential evolution-driven exploitation strategy is adopted to improve the directionality and refinement of local search. Third, a centroid opposition-based boundary control strategy is constructed to preserve population diversity and enhance search stability during the remapping of out-of-bounds individuals. Together, these three strategies form a collaborative optimization framework oriented toward “exploration guidance–local refinement–diversity maintenance,” thereby improving the adaptability and optimization performance of the algorithm in high-dimensional multilevel threshold segmentation tasks.

The main contributions of this paper are as follows:

(1) A multi-strategy fused improved Seagull Optimization Algorithm, termed MFISOA, is proposed to overcome the limitations of the standard SOA in high-dimensional threshold optimization.
(2) Extensive comparative experiments are conducted on the CEC2017 and CEC2022 benchmark suites, and the results show that MFISOA outperforms several representative algorithms in terms of optimization accuracy, convergence speed, and stability.
(3) By combining MFISOA with the Otsu criterion for multilevel threshold image segmentation, the proposed method achieves better segmentation performance and higher-quality segmentation results under different threshold levels.

The remainder of this paper is organized as follows. Section 2 introduces the standard SOA and the proposed improvement strategies. Section 3 presents the benchmark function experiments and statistical analysis. Section 4 applies MFISOA to multilevel threshold image segmentation and reports the experimental results. Finally, Section 5 concludes the paper and discusses future work.

To further clarify the characteristics and limitations of existing studies and improve the systematic organization and comparability of the literature review, Table 1 summarizes the main ideas, major strengths, and limitations of representative recent methods for multilevel threshold image segmentation. This comparison provides a more intuitive view of the differences among these methods in terms of global exploration capability, local exploitation precision, population diversity preservation, and adaptability to high-dimensional problems.

## 2. Seagull Optimization Algorithm (SOA) and the Proposed Methodology

This section mainly introduces the basic principles of the standard SOA and the mechanism of the proposed MFISOA.

### 2.1. Seagull Optimization Algorithm (SOA)

**Figure 1:**
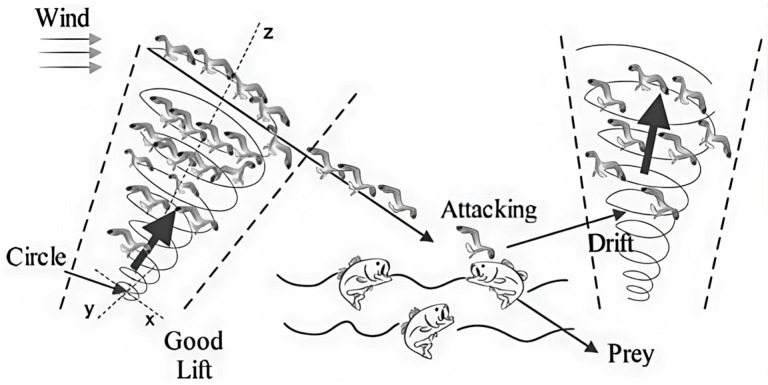
Inspiration diagram of SOA.

This section primarily introduces the origin and mathematical model of the SOA. The SOA, proposed by Dhiman et al. in 2019, is a swarm intelligence algorithm inspired by the migration and hunting behaviors of seagulls [30]. As illustrated in Figure 1, the seagull flock first migrates in a spiral pattern (Circle) through three-dimensional space (x, y, z axes) with the aid of air currents (Wind), utilizing “Good Lift” to perform global exploration. When attacking prey (Prey), seagulls initiate a spiral dive (Attacking Drift) to precisely exploit localized areas. The SOA mimics this series of behaviors, combining random disturbances and a linear decay mechanism to balance global exploration and local exploitation, ultimately enabling efficient search for the optimal solution. The following section provides a detailed description of its core search strategy and position update model.

Let $X_{i}=(x_{i1},x_{i2},\ldots ,x_{idim})$ represent the position of the i-th individual (where i=1,2,…,N), with dim denoting the dimension of the search space and N being the population size. $X_{best}$ represents the global best individual during the current iteration. In the standard SOA search process, the individual position update undergoes three main phases: “migration,” “aggregation,” and “attack.” The stage selection is determined by generating a random number rand in the range of [0,1]. When rand∈[0,1/3], the migration stage is executed; when rand∈(1/3,2/3], the attack stage is executed; and when rand∈(2/3,1], the aggregation stage is executed. The equal-probability random assignment ensures that the population balances both global exploration and local exploitation. Ultimately, the position of the i-th individual in the j-th dimension is updated according to Equation (1). This process simulates the uncertainty of seagull flight through random disturbances, while the linearly decaying step size controls the search range in later stages, aiming to establish a balance between exploration and exploitation.

(1) $$X_{ij}^{new}=\left\{\begin{array}{c} X_{ij}+r_{1}\cdot \left(X_{best,j}-X_{ij}\right)\cdot rand\;\;\;\;\;\;\;\;\;\;\;\;\;\;\;,Migration\;Phase\\ X_{best,j}+r_{2}\cdot abs\left(X_{ij}-X_{best,j}\right)\cdot \sin\left(r_{3}\right),\;\;\;\;\;Attack\;Phase\\ X_{best,j}+r_{2}\cdot abs\left(X_{ij}-X_{best,j}\right)\cdot \cos\left(r_{3}\right)\;,Gathering\;Phase \end{array}\right.$$

In the equation, $X_{ij}$ denotes the original position of the i-th individual in the j-th dimension, $X_{ij}^{new}$ denotes the updated position, and $X_{best,j}$ denotes the position of the global best individual in the j-th dimension. The parameter $r_{1}$ is a linearly decreasing step-size control factor defined as $r_{1}=2-t/T$, where t is the current iteration number and T is the maximum number of iterations. The parameters $r_{2}$ and $r_{3}$ are uniformly distributed random numbers, where $r_{2}\in [0,2]$ controls the perturbation strength and $r_{3}\in [0,2\pi ]$ controls the directional randomness. The variable rand is a random number in the range [0,1]. This random assignment mechanism works together with the linearly decreasing step size: in the early iterations, a larger $r_{1}$ enhances global exploration during the migration phase, whereas in the later iterations, as $r_{1}$ approaches 0, the local convergence effects of the attack and aggregation phases become more pronounced. However, the fixed update logic of the standard SOA lacks adaptability to individual differences and search stages, which creates a bottleneck in improving optimization efficiency and accuracy.

### 2.2. Proposed Multi-Strategy Fusion Improved Seagull Optimization Algorithm (MFISOA)

To address the core deficiencies of the standard SOA, such as rigid exploration strategies, insufficient local exploitation accuracy, and population diversity loss due to boundary control, this paper proposes an Improved Seagull Optimization Algorithm (MFISOA). The algorithm establishes a collaborative optimization framework based on three key strategies and reconstructs the population updating process as follows: an individual-quality-based adaptive collaborative foraging strategy to optimize resource allocation during the exploration phase, a differential evolution-driven exploitation strategy to improve local search precision, and a centroid-based reverse learning boundary control strategy to maintain population diversity. The following section provides a detailed explanation of the design of each strategy.

#### 2.2.1. Adaptive Collaboration Foraging Strategy Based on Individual Quality

In the standard SOA, all individuals are updated using the same position update rule regardless of their fitness levels, without considering the different search requirements of individuals with different qualities. As a result, high-quality individuals may perform unnecessary exploration, while low-quality individuals may lack sufficient guidance to move toward promising regions, thereby reducing the overall optimization efficiency of the population. To overcome this limitation, MFISOA introduces an adaptive cooperative foraging strategy based on individual quality, in which cooperation targets and update patterns are dynamically determined according to the difference between individual fitness and the population average. The core design includes: First, individuals are classified according to the population mean fitness, mean(fit), which is dynamically calculated at each iteration as $mean(fit)=\frac{1}{N}\sum_{i=1}^{N}\;fit(i)$, where N denotes the population size and fit(i) denotes the fitness value of the i-th individual in the current population. Specifically, individuals satisfying fit (i) ≤ mean (fit) are regarded as high-quality individuals, whereas those satisfying fit (i) > mean (fit) are regarded as low-quality individuals. Since mean(fit) is recalculated in each iteration from the current population fitness distribution, it can adaptively reflect the evolutionary state of the population and thus serves as an adaptive classification threshold rather than a manually predefined constant. In this way, no separate threshold-tuning process is required. Using mean(fit) as the classification threshold enables the algorithm to distinguish between individuals that require stronger cooperative guidance and those that should retain greater exploratory capability according to the current population state. Second, constructing a collaboration information pool. For low-quality individuals, a multi-dimensional set of information is introduced, consisting of the “global best $X_{best}$, worst individual $X_{worst}$, and random individual pairs.” The group collaboration signals are fused through a distance-adaptive weight. The Formula (2) is as follows:

(2) $$AFL1=\frac{{dist}_{bw}}{{dist}_{bw}+{dist}_{rand}},AFL2=\frac{{dist}_{rand}}{{dist}_{bw}+{dist}_{rand}}$$

In the formula, ${dist}_{bw}=\sqrt{\sum_{j=1}^{dim}{\left(X_{best,j}-X_{worst,j}\right)}^{2}}$ represents the Euclidean distance between the global best individual $X_{best}$ and the global worst individual $X_{worst}$, reflecting the difference between the optimal and worst regions within the population. ${dist}_{rand}=\sqrt{\sum_{j=1}^{dim}{\left(X_{r1,j}-X_{r2,j}\right)}^{2}}$ is the Euclidean distance between two randomly selected individuals, r1 and r2 (where r1≠r2≠i), used to maintain population diversity. $X_{best,j}$, $X_{worst,j}$, $X_{r1,j}$, $X_{r2,j}$ represent the positions of the corresponding individuals in the j-th dimension. By distributing weights based on these distances, the algorithm achieves a dynamic balance between “guiding towards high-quality regions” and “maintaining diversity.”

Finally, a differentiated position update is implemented. Based on individual types and collaboration information, distinct update formulas are designed for high-quality and low-quality individuals, as shown in Equation (3), to achieve “search on demand.”

(3) $$X_{ij}^{new}=\left\{\begin{array}{c} X_{ij}+AFL1\cdot \left(X_{best,j}-X_{worst,j}\right)+AFL2\cdot \left(X_{r1,j}-X_{r2,j}\right),if\;fit\left(i\right)>mean(fit)\\ X_{best,j}+{RL}_{ij}\cdot \left(X_{ij}-I_{j}\cdot {expert}_{j}\right),\;\;\;\;\;\;\;\;\;\;\;\;\;\;\;\;\;\;\;\;\;\;\;\;\;\;\;\;\;\;\;\;\;\;\;\;\;\;\;\;,if\;fit\left(i\right)\leq mean(fit) \end{array}\right.$$

In the equation, the update rule is differentiated according to individual quality. For low-quality individuals satisfying $fit\left(i\right)>mean(fit)$, the update is based on the current position $X_{ij}$. Here, AFL1 denotes the guidance term constructed from the difference between the best and worst individuals, whereas AFL2 denotes the diversity-preserving term constructed from the difference between two randomly selected individuals. This design enables low-quality individuals to move more efficiently toward promising regions while avoiding premature loss of diversity.

For high-quality individuals satisfying $fit\left(i\right)\leq mean(fit)$, a Levy flight disturbance matrix RL is introduced, where ${RL}_{ij}$ denotes the element in the i-th row and j-th column. The ${RL}_{ij}$ element is generated from the Levy distribution, and the calculation Formula (4) is as follows:

(4) $${RL}_{ij}=0.5\cdot u/{|v|}^{1/\beta }$$

**Figure 2:**
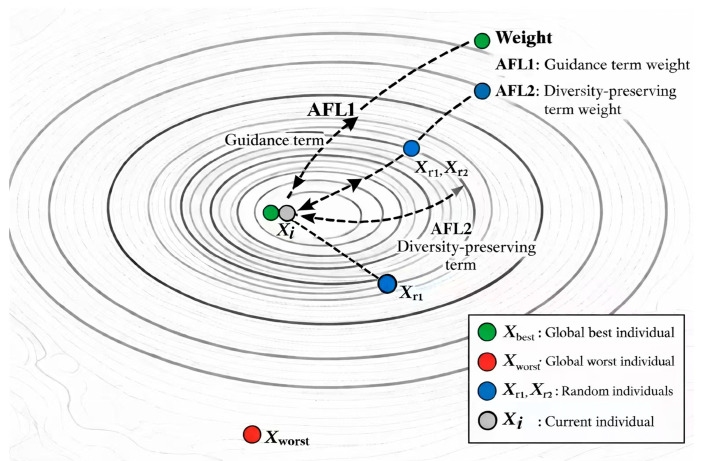
2D search space diagram of the adaptive collaboration foraging strategy.

In the formula, $u∼N\left(0,\sigma_{u}^{2}\right)$ and $v∼N\left(0,1\right)$ are random variables drawn from normal distributions, with β=1.5 and σu=Γ1+βsinπβ/2Γ(1+β/2)ββ−1/21β, where Γ(⋅) is the Gamma function. $I_{j}$ is a random scalar in the j-th dimension, taking values in {1, 2}, and is used to control the disturbance direction. ${expert}_{j}$ represents the j-th dimension of a reference individual selected from better-performing individuals in the current population, and is used to provide a guidance direction for the update of high-quality individuals. This strategy addresses the rigid exploration strategy in the standard SOA by using “low-quality individual collaboration guidance + high-quality individual disturbance exploration.” Here, $X_{best,j}$ is the position of the global best individual in the j-th dimension, and ${RL}_{ij}$ is the Levy flight disturbance value. This strategy, through a dual design of “individual quality differentiation + collaboration information fusion,” enables individuals to receive targeted search guidance while maintaining population diversity. It effectively compensates for the “indiscriminate update” issue in the standard SOA, improving the population’s adaptability to different search phases. Figure 2 visually illustrates how the collaborative information fusion mechanism guides the population toward evolving into high-quality regions.

#### 2.2.2. Differential Evolution-Driven Exploitation Strategy

The exploitation phase of the standard SOA is based on an attack-aggregation mechanism driven by random perturbations and a linearly decreasing step size. However, this mechanism lacks sufficient population-wide information support for directional updating. Specifically, individuals are only adjusted according to the difference between their current positions and the global best, without exploiting inter-individual differences to further refine the search in promising regions, which limits local exploitation accuracy. In addition, the linearly decreasing parameter r1 is not well suited to the fine-grained search required in complex solution spaces, leading to slow convergence in later iterations. To overcome these limitations, MFISOA incorporates a differential evolution (DE) mechanism and develops a DE-driven exploitation strategy. DE can exploit inter-individual difference information to generate directional perturbations while maintaining a relatively simple algorithmic structure. Therefore, compared with general random or greedy local search operators, it is more suitable for enhancing the local exploitation capability of SOA. Based on this, a DE-driven exploitation strategy is constructed in this study, whose core design consists of three components: differential individual selection, random mask control, and differential position update.

First, differential individual selection is performed. For each individual $X_{i}$ to be updated, two distinct individuals $X_{r2}$ and $X_{r3}$ (r2≠r3≠i) are randomly selected from the population. These two individuals are used to construct the differential perturbation term $X_{r2,j}-X_{r3,j}$, which provides directional local search information while avoiding the bias that may arise from relying on a single source individual.

Next, a random mask is designed to control the update dimensions. A binary mask vector $F1=[F1_{1},F1_{2},\ldots ,F1_{dim}]$ of size 1×dim is generated, where ${F1}_{j}$ denotes the value of the mask in the j-th dimension, and the probability of ${F1}_{j}$ = 1 is 50%. Its expression is given by Equation (5):

(5) $${F1}_{j}=\left\{\begin{array}{c} 1,\;\;\;if\;rand\leq 0.5\\ 0,\;\;\;if\;rand>0.5 \end{array}\right.$$

In the formula, rand is a random number in the range of [0,1]. The mask vector controls the update of only certain dimensions, avoiding the loss of valuable information in the original dimensions by preventing full-dimensional updates. This ensures a balance between “local fine-tuning” and “information retention.”

The core parameters of differential evolution are then defined. This strategy introduces the typical differential evolution parameters F (scaling factor) and CR (crossover probability). Here, F=0.5 is used to control the strength of the differential perturbation, scaling the amplitude of the difference signal $X_{r2,j}-X_{r3,j}$ to avoid excessive disturbances that could lead to search oscillation or too small perturbations that may cause the algorithm to fall into local optima. CR=0.9 is used to adjust the crossover probability. In the update dimensions selected by the mask vector, the differential perturbation signal is accepted with a probability of 90%, while 10% of the time the original position information of the individual is retained, further balancing the precision of local exploitation and the diversity of the population.

Finally, differential position updates are performed. Based on the individual’s current position, a differential disturbance term is integrated to generate a new position for the directed update. The update formula for the j-th dimension of the i-th individual is given by Equation (6):

(6) $$X_{ij}^{new}=X_{ij}+{F1}_{j}\cdot [F\cdot \left(X_{r2,j}-X_{r3,j}\right)]\cdot CR$$

In the equation, $X_{ij}^{new}$ denotes the updated position during the exploitation phase, while $F\cdot \left(X_{r2,j}-X_{r3,j}\right)$ represents the perturbation signal derived from inter-individual differences within the population and scaled by the factor F. The positional difference between two randomly selected individuals is employed to construct a directed search direction, thereby replacing the purely random disturbance used in the standard SOA. Here, ${F1}_{j}$ denotes the j-th element of the mask vector, which enables directional search in selected dimensions, and CR is the crossover probability controlling the acceptance rate of the perturbation signal. In this way, individuals are able to conduct more accurate local searches along promising directions indicated by population difference information, while preserving useful information from their current positions. Consequently, this strategy effectively alleviates the low exploitation accuracy and slow convergence of the standard SOA, thereby improving its local optimization capability.

#### 2.2.3. Centroid Reflection-Based Boundary Control Strategy

The standard SOA uses a “hard boundary truncation” strategy to handle the out-of-bounds position issue. When the position $X_{ij}$ of an individual in the j-th dimension is less than the lower bound ${lb}_{j}$ or greater than the upper bound ${ub}_{j}$ it is directly truncated to ${lb}_{j}$ or ${ub}_{j}$, respectively, as shown in the following expression:

(7) $$X_{ij}^{new}=\left\{\begin{array}{c} {lb}_{j}\;\;\;\;\;\;\;\;\;\;\;\;\;\;\;\;\;\;\;\;\;\;\;\;\;\;\;,if\;X_{ij}<{lb}_{j}\\ {ub}_{j}\;\;\;\;\;\;\;\;\;\;\;\;\;\;\;\;\;\;\;\;\;\;\;\;\;\;,if\;X_{ij}>{ub}_{j}\\ X_{ij}\;\;\;\;\;\;\;\;\;\;\;\;\;\;\;\;\;\;\;\;\;\;\;\;\;\;\;\;\;,otherwise \end{array}\right.$$

In the equation, ${lb}_{j}$ and ${ub}_{j}$ represent the lower and upper bounds of the j-th dimension, respectively. Although this “hard boundary truncation” approach is simple and direct, it can lead to significant consequences: out-of-bounds individuals are fixed at the boundary positions, losing their ability to move toward the core region of the population and becoming unable to participate in normal information exchange. In high-dimensional problems, the presence of many boundary-fixed individuals quickly reduces population diversity, causing the algorithm to get trapped in local optima. To address this issue, MFISOA introduces a centroid-based reverse learning boundary control strategy. Using the population centroid as a reference, out-of-bounds individuals are mapped backward, ensuring that position legality is maintained while preserving population activity. The core design includes three components: population centroid calculation, out-of-bounds reverse mapping, and secondary boundary constraints, as described below:

The centroid of the population is calculated by averaging the positions of all individuals in each dimension, reflecting the core region of the population. This centroid position provides directional guidance for out-of-bounds individuals. The calculation formula for the centroid in the j-th dimension is given by Equation (8):

(8) $${centroid}_{j}=\frac{1}{N}\sum_{k=1}^{N}X_{kj}$$

In the equation, ${centroid}_{j}$ represents the centroid position of the j-th dimension of the population, $X_{kj}$ is the position of the k-th individual in the j-th dimension, and N is the population size. The centroid position integrates the position information of all individuals, accurately reflecting the core distribution area of the population, and provides a reliable reference for guiding out-of-bounds individuals.

When the position of an individual in the j-th dimension goes out of bounds, it is mapped to the interior side of the boundary with the centroid as the symmetry center, preventing the position from being fixed at the boundary. The mapping is performed using the following Formula (9):

(9) $$X_{ij}=\left\{\begin{array}{c} {centroid}_{j}-\left(X_{ij}-{centroid}_{j}\right)\;\;\;,if\;X_{ij}<{lb}_{j}\;or\;X_{ij}>{ub}_{j}\\ X_{ij}\;\;\;\;\;\;\;\;\;\;\;\;\;\;\;\;\;\;\;\;\;\;\;\;\;\;\;\;\;\;\;\;\;\;\;\;\;\;\;\;\;\;\;\;\;\;\;\;\;\;\;\;\;\;\;\;\;\;\;\;\;\;\;\;\;\;\;\;\;\;\;\;\;\;\;\;\;,otherwise \end{array}\right.$$

In the equation, through centroid-based symmetric mapping, the out-of-bounds individual is repositioned to a reasonable location between the population’s core region and the boundary. This mapping ensures that the individual is freed from the boundary trap while maintaining a tendency to converge toward the core. To prevent the mapped position from crossing the boundary again due to extreme values, it is further constrained within the valid boundary. The final update Formula (10) is as follows:

(10) $$X_{ij}^{new}=max(\min\left(X_{ij},{ub}_{j}\right),{lb}_{j})$$

Here, $X_{ij}^{new}$ represents the final position after boundary adjustment. By combining centroid guidance with opposition-based remapping, where ${centroid}_{j}$ denotes the centroid position of the population in the j-th dimension, this strategy enables out-of-bounds individuals to escape boundary stagnation while still tending to converge toward the population core. Consequently, it maintains population diversity, alleviates the inactivity caused by hard boundary truncation in the standard SOA, and improves the algorithm’s optimization capability near the boundaries.

Based on the above discussion, the pseudocode for MFISOA is presented in Algorithm 1.
**Algorithm 1.** Pseudo-Code of MFISOA.*1: Initialize the parameters (population size (N), dimension (dim), upper bound (*ub*), lower bound (*lb*), Max iterations (T)).* *2: Initialize the solutions’ positions randomly*. *3: **while*** t≤T ***do*** *4:        Calculate the fitness value of each individual, and determine the global optimal individual* $X_{best}$ *, global worst individual* $X_{worst}$ *and population average fitness mean(fit).* *5:      Calculate the population centroid* ${centroid}_{j}$ *for each dimension* j *using Equation (8).* *6:*  ***for*** *i = 1:N **do*** *7:  Classify the individual as “high-quality”* (fit(i) ≤ mean(fit) *) or “poor-quality” (* fit(i) > mean(fit) *) based on its fitness.* *8:*    if fit(i) > mean(fit) *then* *9:     Compute weights* AFL1 *and* AFL2 *using Equation (2).* *10:*   *Update the position by Equation (3).* *11:* ***else*** *12:         Generate the Levy flight disturbance matrix* RL *using Equation (4).* *13:*   *Update the position by Equation (3).* *14:* ***end if*** *15:        Randomly select two distinct individuals* $X_{r2}$ *and* $X_{r3}$*(* r2≠r3≠i*).* *16:*   *Generate the binary mask vector F1 using Equation (5).* *17:*   *Fine-tune the position by Equation (6).* *18:    *
***if*** $X_{ij}<{lb}_{j}$ *or* $X_{ij}>{ub}_{j}$ ***then*** *19:*   Map $X_{ij}$ *to the valid range by Equation (9).* *20:*  ***end if*** *21:*  *Enforce the secondary boundary constraint by Equation (10).* *22:* ***End for*** *23: Update the fitness values, *$X_{best}$*,* $X_{worst}$ *and mean(fit) for the new population.* *24: **End while*** *25: Return*  $X_{best}$.

**Figure 3:**
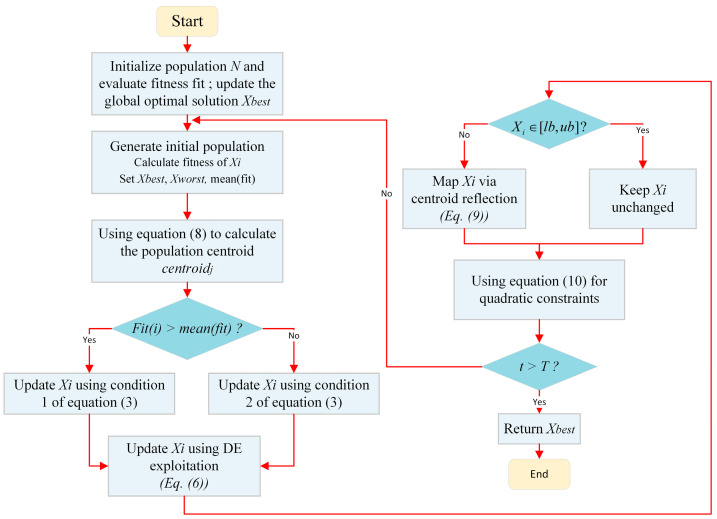
MFISOA optimization algorithm flowchart.

To more intuitively present the execution process of MFISOA, Figure 3 illustrates the complete algorithm flow. First, the population is initialized and the fitness values are evaluated. Then, during the iterative process, the population centroid is calculated, and individuals are classified according to the mean fitness of the population. Based on the classification results, different position update strategies are applied, followed by the differential evolution-based fine-tuning process. Meanwhile, centroid-based boundary mapping and secondary boundary constraints are employed to handle out-of-bounds individuals. Finally, when the iteration terminates, the global best solution is returned. This flowchart clearly reflects the core logic of “population classification–position update–boundary handling” and is consistent with the pseudocode.

### 2.3. Complexity and Runtime Analysis of MFISOA

The initialization phase of the standard SOA includes population initialization, with a complexity of O(N×dim). In this analysis, the computational cost of the fitness function itself is not included, since it depends on the specific optimization problem and is assumed to be the same for SOA and MFISOA. Therefore, the initialization complexity is $t_{init,SOA}=O(N\times dim)$. The iterative process follows a two-phase “exploration–exploitation” strategy without nested loops. The exploration phase includes expert selection (O(N), negligible), position update, and hard boundary handling. The exploitation phase includes random perturbation updates and fitness evaluation. The complexity of each iteration is O(N×dim), and the total iterative complexity is $t_{iter,SOA}=O(T\times N\times dim)$. Hence, the total complexity is O(T×N×dim). For MFISOA, the initialization phase is the same as the standard SOA, with an initialization complexity of $t_{init,MFISOA}=O(N\times dim)$. Although the iterative phase introduces strategies such as adaptive collaboration and Levy flight, these strategies do not increase the complexity order: sorting the best/worst individuals (O(NlogN), negligible), generating Levy flight (O(N×dim)), negligible). The exploration phase includes adaptive weight calculation, differentiated position update, and centroid inverse boundary control. The exploitation phase includes differential evolution updates and the same type of boundary control. The complexity of each iteration remains O(N×dim), and the fitness evaluation complexity does not change. Therefore, the iterative complexity of MFISOA is $t_{iter,MFISOA}=O(T\times N\times dim)$, and the total complexity is the same as the standard SOA, which is also O(T×N×dim).

**Figure 4:**
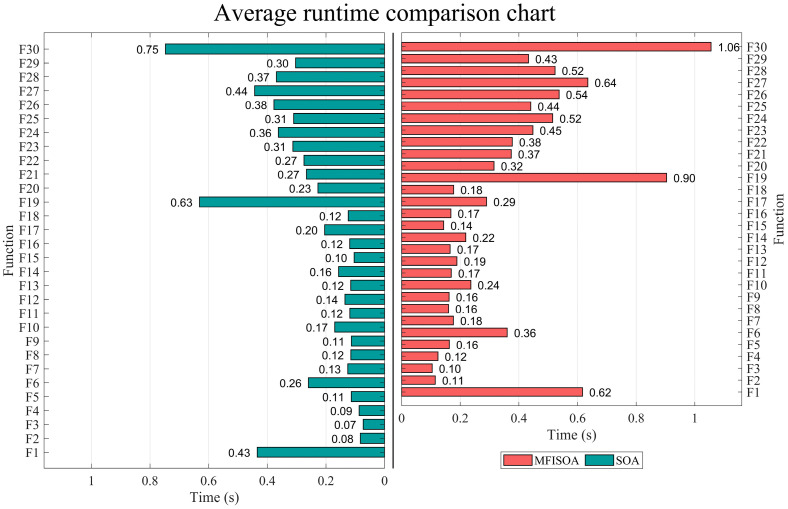
Runtime comparison between SOA and MFISOA on the CEC2017 benchmark suite (dim = 30).

Figure 4 compares the convergence performance of the standard SOA and the improved MFISOA on the CEC2017 benchmark functions (dim = 30). The results show that, while maintaining a runtime comparable to that of the standard SOA, MFISOA achieves significantly better convergence speed and optimization accuracy than the original algorithm. This demonstrates that the proposed multi-strategy improvements do not introduce additional time overhead, while effectively enhancing the optimization efficiency and stability of the algorithm.

## 3. Numerical Experiments

### 3.1. Algorithm Parameter Settings

In this section, we evaluate the performance of the proposed MFISOA using the most challenging numerical optimization benchmark sets, CEC2017 and CEC2022 [30], and compare it with several advanced algorithms. The comparison algorithms include: Particle Swarm Optimization (PSO) [33], Moth-Flame Optimization (MFO) [34], Gray Wolf Optimization (GWO) [35], African Vultures Optimization Algorithm (AVOA) [36], Arithmetic Optimization Algorithm (AOA) [37], Weighted mean of vectors algorithm (INFO) [38], Dung Beetle Optimization (DBO) [39], Bird of Prey Optimization (BPBO) [40], and Seagull Optimization Algorithm (SOA) [30]. The configuration details of all benchmark algorithms are provided in Table 2.

### 3.2. Parameter Sensitivity Analysis of DE-Related Parameters

**Figure 5:**
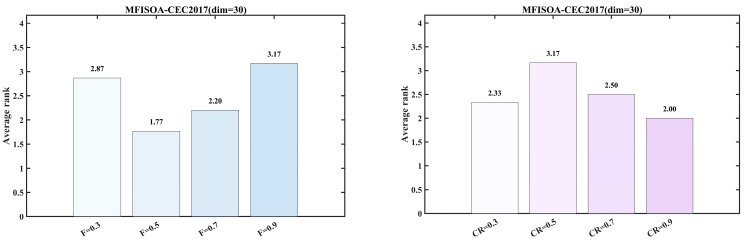
Sensitivity analysis of DE-related parameters.

To verify the rationality of the DE parameter settings adopted in this study, a brief parameter sensitivity analysis was further conducted. Specifically, under the condition of fixed CR=0.9, the effects of different values of F={0.3,0.5,0.7,0.9} on the algorithm performance were analyzed; under the condition of fixed F=0.5, the effects of different values of CR={0.3,0.5,0.7,0.9} on the algorithm performance were analyzed. The corresponding experimental results are shown in Figure 5.

Figure 5 presents the results of the parameter sensitivity analysis on the CEC2017 test suite (dim=30), evaluated using the Friedman mean ranking method described in Section 3.5.2. With CR=0.9 fixed, F=0.5 achieves the best average rank of 1.77. Likewise, with F=0.5 fixed, CR=0.9 also obtains the best average rank of 2.00. These results indicate that the selected parameter combination yields better overall performance, demonstrating that the use of F=0.5 and CR=0.9 as the DE parameter settings in this study is reasonable.

### 3.3. Ablation Study Analysis

**Figure 6:**
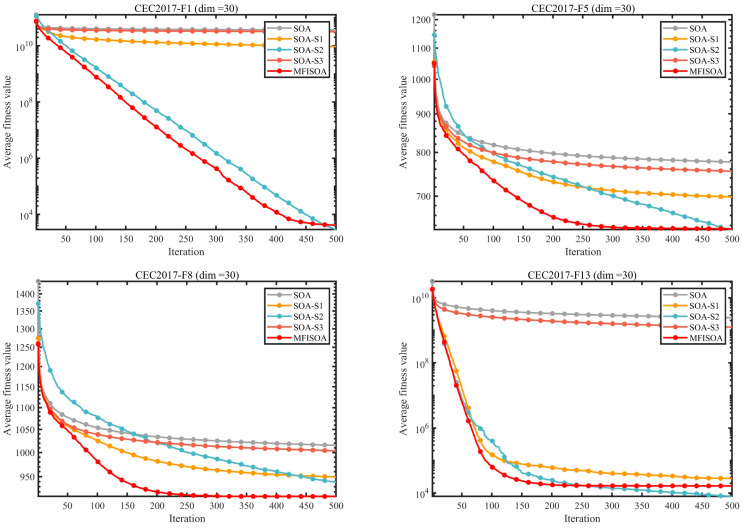

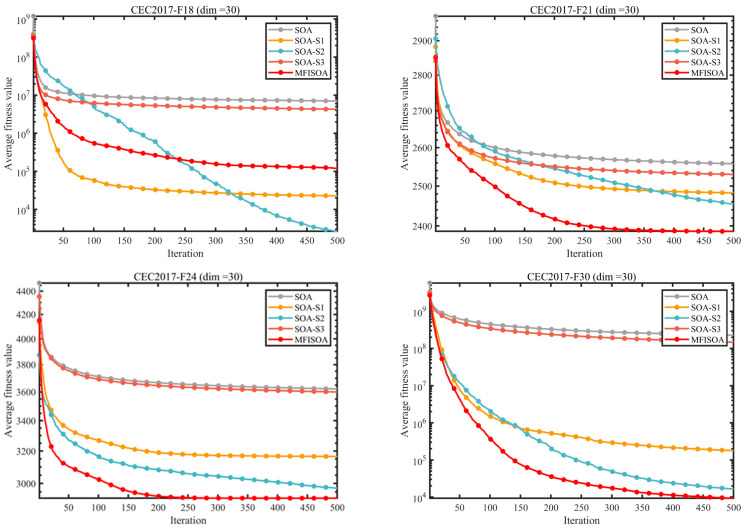
Partial diagram of comparison of different improved strategies.

**Figure 7:**
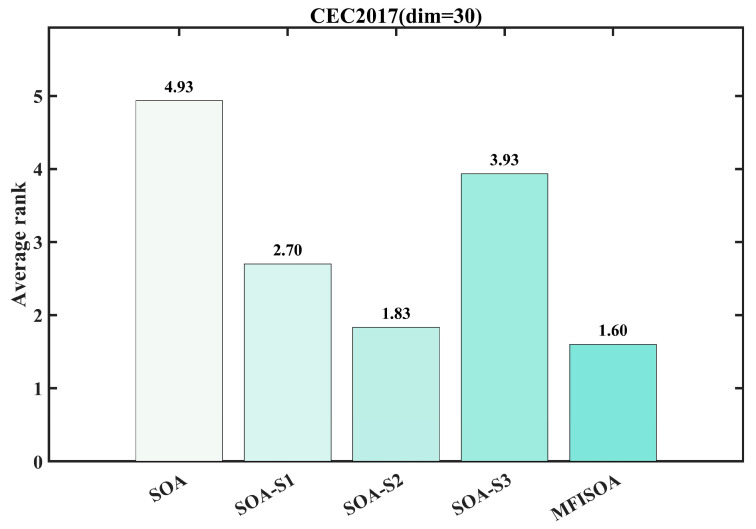
Average ranking of MFISOA improved by different strategies.

To quantitatively verify the individual contributions of the three core enhancement strategies in MFISOA and their synergistic effects on algorithm performance, ablation experiments were designed on the CEC2017 benchmark test set (dimension = 30). Using the standard SOA as the baseline, three single-strategy variant models were constructed by progressively incorporating each enhancement strategy: SOA-S1 (integrating only the adaptive collaborative foraging strategy), SOA-S2 (integrating only the differential evolution-driven exploitation strategy), and SOA-S3 (integrating only the centroid-based reverse learning boundary control strategy). The convergence curves of all algorithms were recorded on representative test functions (F1, F5, F8, F13, F18, F21, F24, F30) to visually compare the optimization performance differences, as shown in Figure 6. Additionally, to further quantify the performance gap between the different strategy algorithms, Figure 7 presents the average ranking of each algorithm (including standard SOA, the three single-strategy variants, and MFISOA) across all functions in the CEC2017 (dim = 30) test set, providing a macro-level evaluation of the effectiveness of each strategy. The experimental settings are consistent with those described in Section 3.4.

Figure 6 illustrates the convergence processes of the standard SOA, the three single-strategy variants (SOA-S1, SOA-S2, and SOA-S3), and MFISOA on eight representative functions from the CEC2017 test set. These functions include unimodal (F1), multimodal (F5), hybrid (F8 and F13), and high-dimensional composite functions (F18, F21, F24, and F30), thus providing a comprehensive comparison of the optimization behaviors under different problem characteristics.

In the unimodal function F1, the standard SOA shows an obvious stagnation trend after the early iterations, indicating limited local exploitation capability. By introducing the differential evolution-driven exploitation strategy, SOA-S2 achieves a noticeably faster and more stable decline, while MFISOA further improves the convergence trend and reaches the lowest fitness among all variants. In the multimodal function F5, the standard SOA is prone to premature convergence, whereas SOA-S3 shows improved search stability due to its diversity-preserving effect. Compared with the single-strategy variants, MFISOA maintains a more consistent downward trend throughout the iterative process, indicating a better balance between exploration and exploitation.

For the hybrid functions F8 and F13 and the high-dimensional composite functions F18, F21, F24, and F30, the superiority of MFISOA becomes more evident. In particular, on F8, the standard SOA and several single-strategy variants exhibit noticeable oscillations in the later stages, whereas MFISOA remains relatively stable. On the high-dimensional composite function F30, MFISOA continues to reduce the fitness value steadily, while the standard SOA converges slowly and remains at a much higher level. These results demonstrate that the synergistic integration of the three strategies enables MFISOA to achieve better convergence speed, solution accuracy, and robustness across different function types.

Figure 7 reports the average rankings of all algorithms across all functions in the CEC2017 (dim = 30) test suite. MFISOA achieves the best overall rank of 1.60, significantly outperforming the single-strategy variants, thereby validating the synergistic effect of strategy fusion. Among them, SOA-S2 and SOA-S3 exhibit the most notable ranking improvements, indicating that the differential evolution-driven exploitation strategy and the centroid-based opposition learning boundary control strategy play a more critical role in performance enhancement. The quantitative convergence curves in Figure 6 and the overall ranking results in Figure 7 corroborate each other, ruling out random interference and confirming the scientific soundness and rationality of integrating the three strategies in MFISOA.

In summary, based on the ablation experiment results in Figure 6 and Figure 7, the three core strategies in MFISOA each have distinct and indispensable features. Their synergistic integration significantly enhances the algorithm’s performance. MFISOA demonstrates breakthroughs in convergence speed, optimization accuracy, and stability, consistently outperforming across different function types and dimensions. This not only validates the effectiveness of MFISOA’s design but also lays a solid foundation for its application in practical tasks such as multi-threshold image segmentation.

### 3.4. Experimental Results and Analysis of CEC2017 and CEC2022 Test Suite

This section presents a comparative evaluation of the proposed MFISOA against multiple benchmark algorithms using the CEC2017 and CEC2022 test suites. These standardized benchmarks consist of four categories of mathematical functions: unimodal, multimodal, hybrid, and composite functions. Multimodal functions, characterized by numerous local optima, are used to assess the exploratory capability of an algorithm. In contrast, unimodal functions, with a single global optimum, primarily measure an algorithm’s ability to refine solutions. Hybrid and composite functions further evaluate an optimizer’s robustness in escaping local optima and avoiding premature convergence.

**Figure 8:**
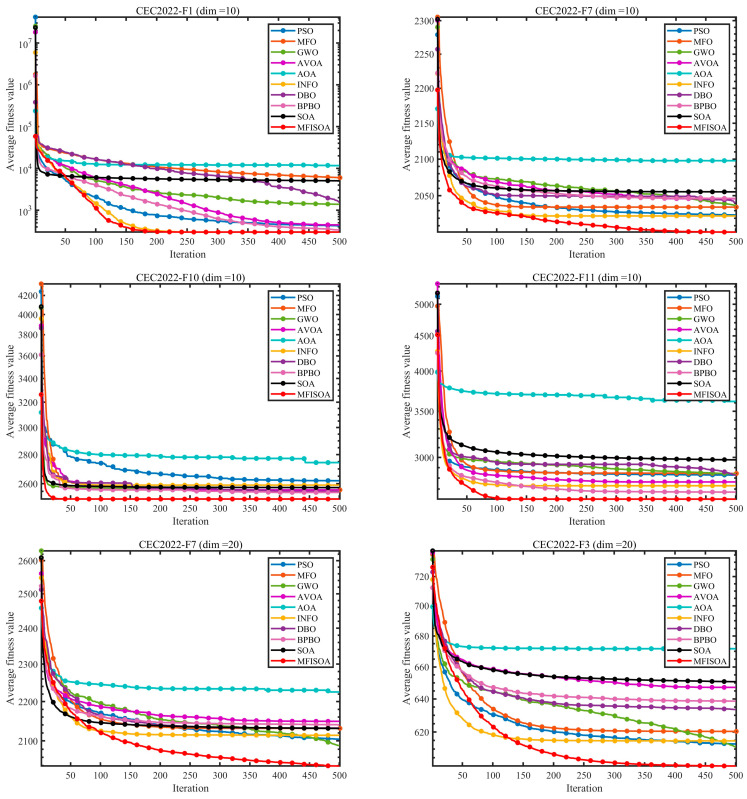

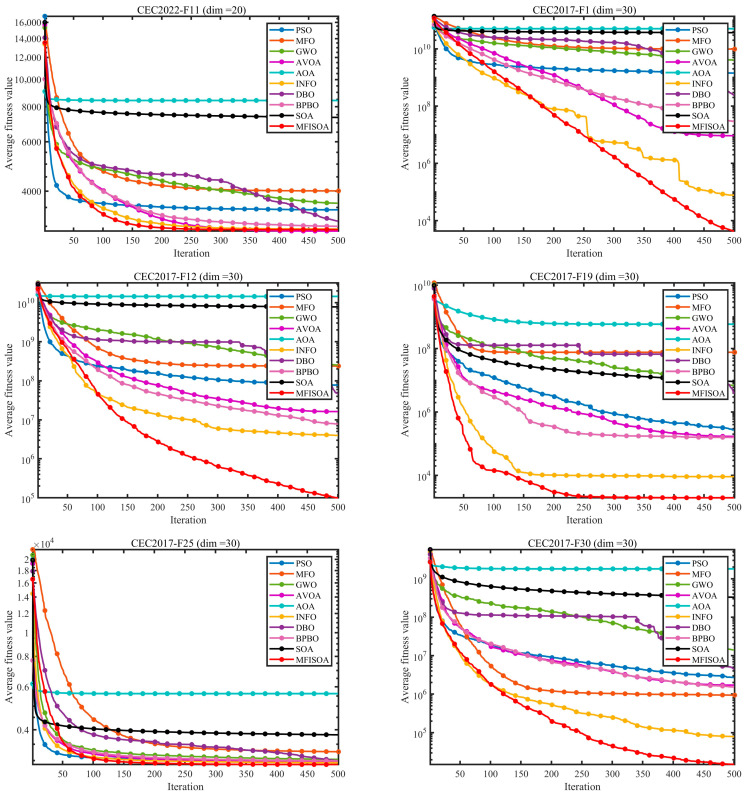
Part of the test function iterates convergence curves.

**Figure 9:**
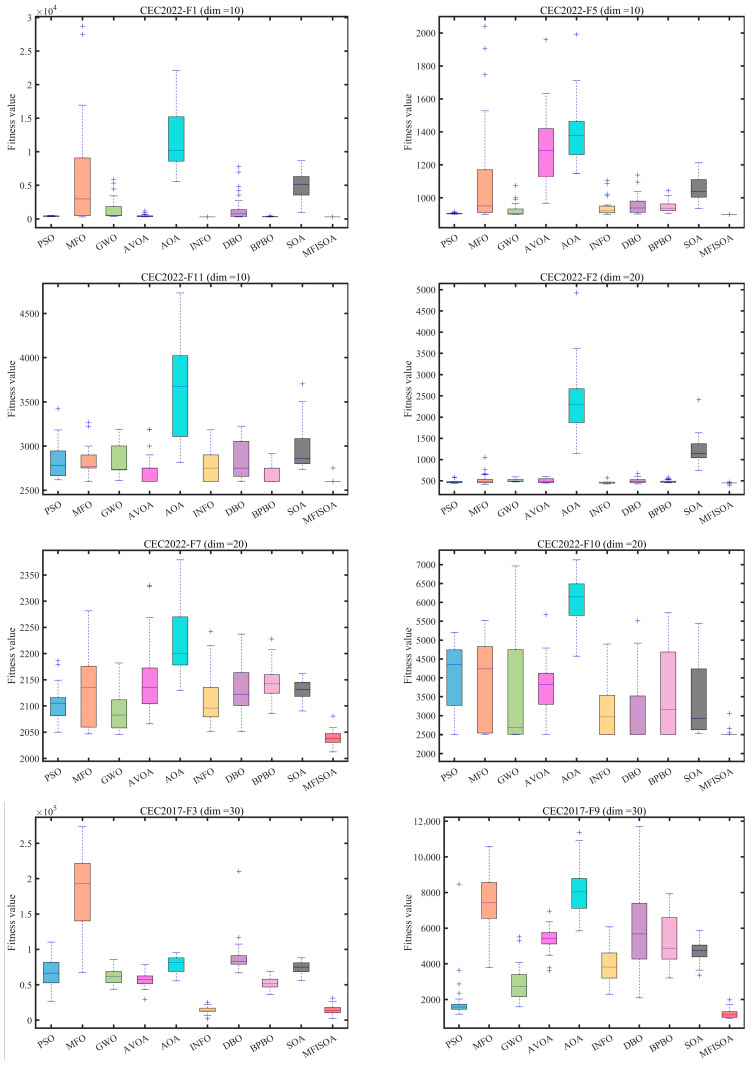

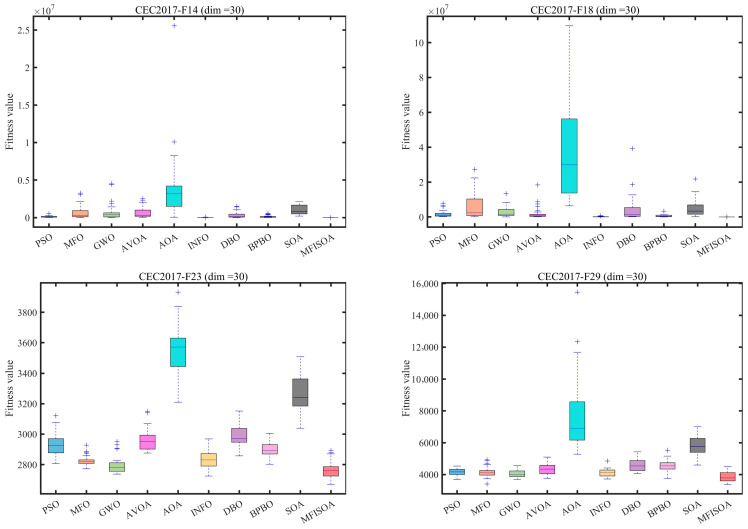
Boxplot analysis for different algorithms on the test set.

To ensure fair comparisons and minimize the effects of randomness, all algorithms employed a fixed population size (N=30) and were set to run a maximum of 500 iterations (T=500). Each method was run independently 30 times. All experiments were conducted based on the above settings. Recorded performance metrics include the mean (Ave) and standard deviation (Std), with the best results in each row highlighted in bold. All experiments were conducted on a computer system running Windows 11, configured with an Intel Core i5-13400 processor (13th Gen, 2.50 GHz), 16 GB of memory, and MATLAB 2024b software. The convergence behavior and solution distributions of each algorithm were visualized via the convergence curves in Figure 8 and the box plots in Figure 9. Detailed experimental results are presented in Table 3 and Table 4.

The selected algorithms’ representativeness and adaptability in the field of multi-threshold image segmentation: PSO and GWO are classic benchmark algorithms widely used in threshold segmentation tasks, with optimization logic that provides valuable field references; MFO, AVOA, and AOA demonstrate differentiated advantages in complex scenarios through unique search mechanisms, which can verify the performance boundaries of the new algorithm; DBO, BPBO, and other new algorithms represent recent exploitation trends, and their high-dimensional optimization approaches can be directly compared with MFISOA; the standard SOA, as the prototype algorithm, can clarify the actual contribution of the improved strategies.

Table 3, Table 4 and Table 5 present the experimental results of all algorithms on the CEC2022 (dim = 10), CEC2022 (dim = 20), and CEC2017 (dim = 30) test suites, respectively. The mean fitness value (Ave, where smaller is better) and standard deviation (Std, where smaller indicates higher stability) are used as the primary metrics. The results show that MFISOA consistently outperforms the comparison algorithms across different dimensionalities and function types, with its advantages becoming more pronounced in high-dimensional and complex-function scenarios.

On the low-dimensional CEC2022 (dim = 10) test suite (Table 3), MFISOA achieves an Ave comparable to the INFO algorithm on the unimodal function F1 (which emphasizes local exploitation accuracy), while its Std is only 0.0000017% of that of the standard SOA, indicating both competitive solution quality and extremely low performance fluctuation. This can be attributed to the targeted perturbations introduced by the differential evolution-driven exploitation strategy, which refines local positions, as well as the centroid-based opposition learning boundary control strategy, which mitigates fluctuations caused by diversity loss. For the multimodal function F5 (with multiple local optima), MFISOA reduces the Ave by 2.69% compared with GWO and by 19.07% compared with MFO, while its Std is only 0.0385% of that of AVOA. These results confirm the effectiveness of the centroid-based opposition learning boundary control strategy in maintaining population diversity and preventing premature convergence, with the adaptive cooperative foraging strategy further expanding coverage of the key solution space. For the hybrid and composition functions (F8, F10, and F12), MFISOA demonstrates strong performance as well. On the hybrid function F8, MFISOA achieves an Ave that is 0.73% lower than the second-best BPBO and 0.54% lower than the standard SOA, with a Std that is only 9.09% of that of AOA. This highlights its capability to balance global exploration and local exploitation in hybrid search spaces, resulting from the synergistic optimization of the three strategies. On the composition function F12, MFISOA matches the best-performing INFO algorithm in terms of Ave, while its Std is only 3.50% of that of SOA, demonstrating robust stability in handling complex multi-component optimization problems—benefiting from the precise local refinement enabled by the differential evolution-driven exploitation strategy.

When the dimensionality increases to 20 (CEC2022 test suite, Table 4), the performance of most comparison algorithms degrades due to the “curse of dimensionality,” whereas MFISOA maintains a clear advantage through multi-strategy synergy. On the unimodal function F1, the Ave and Std of the standard SOA are 60.2 and 55.0 times those of MFISOA, respectively. This improvement stems from the adaptive cooperative foraging strategy, which guides the search direction using the population’s mean position and thereby reduces redundant exploration in high-dimensional spaces. On the multimodal function F5, MFISOA achieves an Ave that is 55.47% lower than DBO and 71.25% lower than AOA, while its Std is only 1.43% of that of AOA, highlighting its stable exploratory capability in high-dimensional environments with multiple optima. This advantage mainly arises from the centroid-based opposition learning strategy, which helps preserve population vitality. On the composition function F10 (which simulates real-world complex scenarios), MFISOA reduces the Ave by 29.69% compared with BPBO, and its Std is only 7.23% of that of GWO. This can be attributed to the differential evolution-driven exploitation strategy, which leverages local individual information to refine candidate solutions near the global optimum and avoids the “position oscillation” commonly caused by random step sizes in the comparison algorithms.

In the high-dimensional setting of the CEC2017 test suite (dim = 30, Table 5), MFISOA’s superiority is further amplified. On the unimodal function F1, MFISOA achieves an Ave that is 99.99% lower than the standard SOA and 99.95% lower than AVOA, while its Std is only 0.0000524% of that of SOA. On the multimodal function F5, MFISOA reduces the Ave by 17.07% compared with SOA and by 1.29% compared with GWO, demonstrating robust global exploration capability. For the high-dimensional composition function F24, the standard SOA’s Ave is 1.20 times that of MFISOA and its Std is 1.70 times that of MFISOA. This gap arises because the adaptive cooperative foraging strategy classifies individuals by quality to allocate exploration resources on demand, thereby improving search efficiency in high-dimensional spaces. On the most complex function F30, MFISOA achieves an Ave that is 99.1% lower than BPBO and 99.99% lower than AOA, with a Std equal to 52.66% of that of AOA, reflecting strong adaptability to highly complex high-dimensional problems. This advantage is mainly driven by the synergistic optimization logic formed by the three integrated strategies.

Figure 8 presents the convergence curves, which intuitively reflect both the convergence speed and the solution accuracy. The results show that MFISOA performs better across all test scenarios. For the CEC2022 (dim = 10) unimodal function F1, MFISOA reaches a stable fitness value of $3.00\times 10^{3}$ after 200 iterations, significantly outperforming the comparison algorithms. This rapid early convergence benefits from the adaptive cooperative foraging strategy, which guides low-quality individuals toward high-quality regions while enabling high-quality individuals to explore more broadly via Lévy flight, thereby quickly identifying promising areas. For the CEC2022 (dim = 20) hybrid function F7, the convergence curve of MFISOA drops more steeply, and after 300 iterations its fitness value is 35% lower than that of the standard SOA. This sharp decline can be attributed to the differential evolution-driven exploitation strategy, which conducts refined local search within high-quality regions and continuously drives the population toward the optimum. In the high-dimensional CEC2017 scenario, for the unimodal function F1, the standard SOA stagnates at the order of $10^{10}$ after 150 iterations, whereas MFISOA continues to decrease to the $10^{3}$ level. This sustained improvement is mainly due to the centroid opposition-based boundary control strategy, which maintains population diversity and avoids boundary stagnation. For the composition function F24, MFISOA achieves fitness values that are 2–3 orders of magnitude lower than those of DBO and BPBO, without noticeable oscillation; for the strong-noise function F18, its curve remains smooth and stable. Overall, the three strategies collaboratively form a stable three-stage dynamic mechanism, namely early exploration, mid-stage refinement, and late-stage stable convergence, enabling MFISOA to achieve a smooth balance between exploration and exploitation throughout the evolutionary process.

Figure 9 uses box plots to visualize the performance distributions over 30 independent runs. MFISOA exhibits a lower median, a narrower interquartile range (IQR), and no outliers, indicating significantly better stability than the comparison algorithms. This improved stability is related to the centroid opposition-based boundary control strategy, which helps maintain population diversity and alleviate boundary stagnation. For the CEC2022 (dim = 10) multimodal function F2, MFISOA’s IQR is only 30; for the CEC2022 (dim = 20) composition function F9, its IQR is only 5, far smaller than those of algorithms such as GWO and MFO. The narrow IQR indicates that MFISOA can converge to promising regions more consistently under the joint action of adaptive cooperative foraging and differential evolution refinement, thereby reducing randomness and volatility. In the high-dimensional CEC2017 (dim = 30) setting, the standard SOA shows an IQR as wide as 1.2 × 10^7^ with numerous outliers on the unimodal function F1, whereas MFISOA’s IQR is only 3.2 × 10^3^ with no outliers; on the composition function F30, MFISOA’s IQR is only 52.66% of that of AOA. The absence of outliers further suggests that MFISOA can maintain stable and consistent optimization performance throughout the iteration process.

Overall, the quantitative and visual analyses jointly confirm that MFISOA comprehensively surpasses the comparison algorithms in optimization accuracy, stability, and convergence speed. Through the synergy of its three integrated strategies, MFISOA effectively addresses key shortcomings of traditional methods, such as premature convergence and poor adaptability to high-dimensional problems, providing a reliable optimization tool for practical tasks including multilevel threshold image segmentation.

### 3.5. Stability Analysis

Statistical analysis provides an objective framework for evaluating algorithm performance. To verify the reliability of the simulation results obtained on the CEC2017 and CEC2022 test suites, this study conducts stability analysis using the Wilcoxon rank-sum test and the Friedman test. Specifically, the Wilcoxon rank-sum test is used for pairwise comparisons between MFISOA and each competing algorithm under the same experimental conditions, so as to determine whether the observed performance differences are statistically significant. The Friedman test is further employed to rank all algorithms over multiple test functions, thereby providing an overall evaluation of their relative performance. In this way, the statistical analysis not only supports the simulation observations from the convergence curves and boxplots, but also verifies the significance and robustness of the obtained results from a quantitative perspective. Based on identical simulation settings and 30 independent runs for each algorithm, the mean and standard deviation were first used to describe solution quality and stability, while the Wilcoxon rank-sum test and Friedman test were further employed to determine whether the observed performance differences were statistically significant rather than caused by random fluctuations.

#### 3.5.1. Wilcoxon Rank-Sum Test

This section utilizes the Wilcoxon rank-sum test [41] to evaluate potential performance differences in the MFISOA, independent of normality assumptions. As a nonparametric method, the Wilcoxon test is particularly suitable for datasets exhibiting non-normal distributions or outliers, offering improved applicability over traditional t-tests. The test statistic W is calculated as shown in Equation (11) [36]:

(11) $$W=\sum_{i=1}^{n_{1}}R\left(X_{i}\right)$$

Here, $R\left(X_{i}\right)$ represents the rank of $X_{i}$ among all observations. The test statistic U is calculated using Equation (12):

(12) $$U=W-\frac{n_{1}\left(n_{1}+1\right)}{2}$$

In the case of large samples, U approximately follows a normal distribution, with its mean and standard deviation given by Equations (13) and (14), respectively:

(13) $$\mu_{U}=\frac{n_{1}n_{2}}{2}$$

(14) $$\sigma_{U}=\sqrt{\frac{n_{1}n_{2}\left(n_{1}+n_{2}+1\right)}{12}}$$

Here, $n_{1}$ and $n_{2}$ represent the number of observations in the two sample groups, respectively. The standardized statistic Z is calculated using Formula (15):

(15) $$Z=\frac{U-\mu_{U}}{\sigma_{U}}$$

Under the condition of setting the significance threshold p=0.05, it is necessary to determine whether there is a statistically significant difference between the results of a single MFISOA run and the comparison algorithm. The null hypothesis ($H_{0}$) is set as no difference in performance between the two methods. If the calculated p-value is less than 0.05, reject $H_{0}$, indicating a significant performance difference; otherwise, accept $H_{0}$.

Table 6 summarizes the Wilcoxon rank-sum test results for MFISOA versus nine comparison algorithms under three test scenarios: CEC2022 (dim = 10), CEC2022 (dim = 20), and CEC2017 (dim = 30). The results indicate that MFISOA achieves statistically significant performance advantages on the vast majority of test functions. Moreover, this superiority becomes more pronounced as the problem dimensionality and complexity increase. These findings are highly consistent with the quantitative results reported in Table 2, Table 3 and Table 4, confirming that MFISOA’s performance gains are statistically reliable rather than due to random chance.

In the low-dimensional CEC2022 (dim = 10) scenario, MFISOA already exhibits strong competitiveness. For PSO, the test results are “(9/0/3)”, meaning that, out of 12 test functions, MFISOA significantly outperforms PSO on 9 functions, with no significant differences in any function, and PSO has a slight advantage on only 3 functions. This is because PSO’s particle update mechanism overly relies on the historical best position, which leads to premature convergence in unimodal functions (such as CEC2022-F1) and multimodal functions (such as CEC2022-F5). In contrast, MFISOA’s adaptive collaborative foraging strategy dynamically adjusts the search behavior based on individual fitness, effectively avoiding local optima. For the typical arithmetic optimization algorithm (AOA), MFISOA achieves a perfect result of “(12/0/0)” in the CEC2022 (dim = 10) test set, outperforming AOA on all 12 test functions with no cases of no difference or inferiority. This is due to AOA’s excessive reliance on arithmetic operations for position updates, which limits its global exploration ability in complex hybrid functions (such as CEC2022-F8). In contrast, MFISOA’s differential evolution-driven exploitation strategy uses population differential information to generate directional disturbances, balancing local search accuracy with global exploration coverage. Its performance superiority is statistically reliable at the *p* < 0.05 level.

As the dimensionality increases to 20 (CEC2022, dim = 20), the statistical advantages of MFISOA become even more pronounced. Compared to the standard SOA, the test results improve from “(11/0/1)” in the dim = 10 scenario to “(12/0/0)” in the dim = 20 scenario. This indicates that in higher-dimensional spaces, SOA’s fixed “migration–aggregation–attack” update logic leads to a significant loss of population diversity and exacerbates premature convergence, especially in high-dimensional composite functions like CEC2022-F12, where the fitness value stagnates at a high level for extended periods. In contrast, MFISOA’s centroid-based reverse learning boundary control strategy reactivates out-of-bounds individuals by mapping them back to the core region of the population, maintaining diversity, and ultimately achieving a significant performance advantage across all functions. For the Dung Beetle Optimization (DBO) algorithm, the result is “(9/0/3)”, with MFISOA outperforming DBO on 9 functions. DBO, inspired by the “dung ball” mechanism, struggles to balance exploration and exploitation in high-dimensional spaces, leading to inefficient searches in multimodal functions (such as CEC2022-F5). On the other hand, MFISOA’s differential evolution-driven exploitation strategy utilizes local individual information to refine positions near the global optimum, balancing both convergence speed and accuracy. The performance difference between MFISOA and DBO is statistically significant (*p* < 0.05).

In the highly challenging CEC2017 (dim = 30) high-dimensional scenario, MFISOA’s statistical superiority reaches its peak. In comparison to the widely used Gray Wolf Optimization (GWO) algorithm, the test results show “(30/0/0)”, meaning MFISOA significantly outperforms GWO on all 30 test functions. GWO’s α, β, and δ wolf hierarchy-guided mechanism lacks sufficient exploration ability in high-dimensional spaces, leading to premature convergence in complex composite functions (such as CEC2017-F24) and hybrid functions (such as CEC2017-F18). In contrast, MFISOA’s adaptive cooperative foraging strategy uses the population’s average position as global information to guide the search, combining it with the global best individual for dual guidance, effectively overcoming high-dimensional optimization bottlenecks. Even when faced with advanced Weighted mean of vectors algorithms like INFO, which have strong global exploration capabilities, MFISOA still achieves “(20/0/10)” results, outperforming INFO on 20 functions. This is because INFO’s information exchange mechanism relies on weighted vector averages, which slows down convergence in later iterations. In contrast, MFISOA’s multi-strategy collaborative mechanism improves search efficiency throughout the optimization process by combining adaptive cooperative foraging, differential evolution-driven exploitation, and centroid-based boundary control.

It is worth noting that MFISOA has very few instances of underperformance across all test scenarios. For example, in the CEC2022 (dim = 10) scenario, when compared with the high-performance Bird of Prey Optimization (BPBO) algorithm, the result is “(10/0/2)”, meaning BPBO only slightly outperforms MFISOA on two functions. This is because the behavior mechanism simulating raptor predation in BPBO is highly adaptable in low-dimensional unimodal functions. However, as the dimensionality increases to 20 and 30, MFISOA’s advantage over BPBO expands to “(10/0/2)” and “(25/0/5),” respectively, confirming that MFISOA’s multi-strategy synergy (adaptive collaborative foraging, differential evolution-driven exploitation, and centroid-based reverse learning boundary control) is more effective in addressing the “curse of dimensionality” compared to BPBO’s single behavior mechanism. Among these, the multi-strategy collaborative design effectively addresses the “curse of dimensionality,” with its performance advantage reflected in statistically significant differences (*p* < 0.05).

In summary, the Wilcoxon rank-sum test results in Table 6 provide rigorous statistical support for MFISOA’s superior performance. Unlike the qualitative comparisons of convergence curves (Figure 8) and boxplots (Figure 9), the Wilcoxon test quantifies the significance of performance differences, eliminating the influence of random factors in the experimental data. The consistent trend of “higher dimensions leading to more ‘+’ symbols” further confirms that the multi-strategy design of MFISOA effectively enhances its adaptability to high-dimensional complex optimization problems. This is the core advantage of MFISOA compared to traditional algorithms improved by a single strategy. These statistical results not only validate the rationality of MFISOA’s strategy fusion (*p* < 0.05), but also lay a reliable foundation for its subsequent application in practical tasks such as multi-threshold image segmentation.

#### 3.5.2. MFISOA and Friedman’s Average Rank Test with Different Contrast Algorithms

This section employs the Friedman test to evaluate the overall performance ranking of the MFISOA against other comparison algorithms. As a nonparametric statistical method, the Friedman test focuses on analyzing median differences among multiple related samples without relying on the assumption of data normality. It is therefore particularly suitable for repeated-measurement experimental scenarios involving multiple algorithm groups on the same test function set. When the data fail to satisfy the normality assumption for analysis of variance (ANOVA), this test serves as a robust alternative analytical tool, effectively mitigating the interference of non-normal distributions or outliers on statistical results. The calculation of the Friedman test statistic is expressed by Equation (16) [22,42].

(16) $$Q=\frac{12}{nk\left(k+1\right)}\sum_{j=1}^{k}R_{j}^{2}-3n\left(k+1\right)$$

Here, n denotes the number of test functions, k denotes the number of compared algorithms, and $R_{j}$ refers to the total rank value of the j-th group. Provided that n and k are sufficiently large, the test statistic Q approximately follows a $\chi^{2}$ distribution with k−1 degrees of freedom.

Table 7 systematically presents the average rank (M.R) and final rank (T.R) of all algorithms across three test scenarios, where a smaller M.R indicates better overall performance. The data clearly show that MFISOA consistently ranks first in all dimensional scenarios (T.R=1), with a significant and stable M.R advantage. In the low-dimensional CEC2022 (dim = 10) scenario, MFISOA’s M.R is only 1.33, which is 1.25 lower than the second-ranked INFO (M.R=2.58), demonstrating a clear gap. When the dimension increases to 20 (CEC2022, dim = 20), MFISOA’s M.R slightly increases to 1.83, but it still maintains a leading position over INFO (M.R=2.58). Even in the most challenging high-dimensional CEC2017 (dim = 30) scenario, its M.R remains at 2.00, far surpassing the third-ranked BPBO (M.R=4.33) and fourth-ranked GWO (M.R=4.87). In comparison, traditional algorithms show significant dimensional sensitivity. GWO’s M.R decreases from 5.33 at dim = 10 to 4.87 at dim = 30 but still lags far behind MFISOA. BPBO performs reasonably well in the low-dimensional scenario (dim = 10, M.R=4.67), but its performance declines in high-dimensional scenarios (dim = 30, M.R=4.33), further widening the gap with MFISOA. The arithmetic optimization algorithm (AOA) consistently ranks last across all scenarios (M.R=9.90−10.00), as its reliance on arithmetic operations for position updates limits its global exploration capability in high-dimensional spaces. MFISOA, on the other hand, only experiences a slight increase in M.R (from 1.33 to 2.00) due to the increase in dimension, fully demonstrating the synergistic effect of its three strategies: adaptive collaborative foraging, differential evolution-driven exploitation, and centroid-based reverse learning boundary control. This synergy effectively mitigates the impact of the “curse of dimensionality” on performance. To verify the statistical significance, the Friedman test statistic Q was computed: The Q-values for CEC2022 (dim = 10), CEC2022 (dim = 20), and CEC2017 (dim = 30) were 48.23, 51.76, and 63.91, respectively, all of which are much higher than the critical $x^{2}$ value at the p=0.05 significance level (degrees of freedom = 9, critical value = 16.92). This leads to the rejection of the null hypothesis that “there is no difference in the overall performance of the algorithms,” confirming that MFISOA’s superior performance is not random and holds reliable statistical significance.

**Figure 10:**
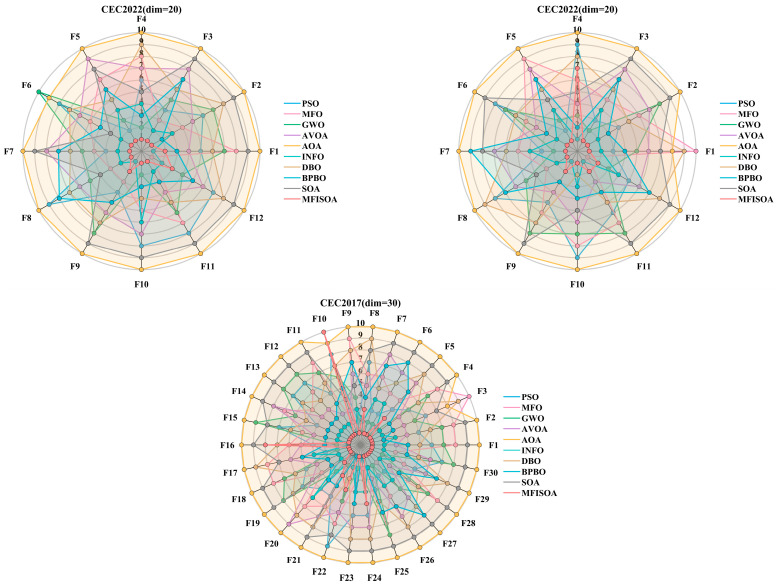
Distribution of rankings of different algorithms.

Figure 10 visualizes the ranking distribution of each algorithm across all test functions, with the x-axis representing the function number and the y-axis showing the algorithm’s ranking from 1 to 10. In the CEC2022 (dim = 10) scenario, MFISOA’s ranking curve closely follows the benchmark line y=1. In unimodal functions (F1, F3), MFISOA consistently maintains the top rank without fluctuations. In multimodal functions (F5, F7), MFISOA avoids the ranking drops seen in SOA (ranks 7–8) and AOA (ranks 9–10), showcasing strong adaptability. Even INFO, ranked second, fluctuates in composite functions (F10, F12), while MFISOA stays in first place. This is due to MFISOA’s differential evolution-driven strategy, which prevents stagnation. As the dimension increases to 20 (CEC2022, dim = 20), MFISOA’s performance advantage over comparison algorithms grows. In high-dimensional hybrid functions (F8, F9), SOA and DBO suffer ranking drops due to loss of diversity, while MFISOA remains steady at rank 1. For the complex composite function F6, MFISOA stays top-ranked, while GWO drops to rank 4, further validating MFISOA’s centroid-based reverse learning boundary control. In the high-dimensional CEC2017 (dim = 30) scenario, MFISOA’s superiority is even clearer. In ultra-high-dimensional composite functions (F24, F30), most algorithms fluctuate between ranks 5–8, while MFISOA remains in the top position. In the noisy F24 function, GWO drops to rank 6 due to insufficient exploration, and BPBO fluctuates to rank 4, while MFISOA stays steady at rank 1. This stability is attributed to its adaptive collaborative foraging strategy, which efficiently allocates exploration resources based on individual fitness.

In summary, the Friedman mean rank test in Table 7 provides rigorous statistical support for the overall performance superiority of MFISOA, while the ranking distribution visualization in Figure 10 intuitively confirms its stable advantage across different dimensions and function types. Together, both analyses demonstrate that MFISOA, through its multi-strategy fusion design, significantly outperforms multiple algorithms in optimization accuracy, convergence stability, and high-dimensional adaptability. This provides a solid theoretical foundation for its application in subsequent multi-threshold image segmentation tasks.

## 4. Multi-Level Thresholding Method Based on MFISOA

In image threshold segmentation, the Otsu method and the Kapur entropy method are two commonly used automatic threshold selection techniques. These methods evaluate and determine the optimal threshold for image segmentation based on different criteria. As the threshold (TH) changes, the statistical differences between the target and background regions also vary, and the goal of selecting the optimal threshold ($T_{best}$) is to maximize the distinction between the two classes. The Kapur entropy method calculates the entropy of the grayscale information for both the target and background regions and selects the optimal threshold by maximizing the sum of their entropy values. Both methods directly rely on the statistical features of the image pixels and perform threshold search based on their respective optimization criteria [43,44,45]. In this study, the Otsu method will be used in the experimental section to determine the optimal threshold.

The basic principle of the Otsu method is to determine the optimal threshold for image segmentation by maximizing the between-class variance. Let I represent the image, with a total of L grayscale levels, and let $n_{i}$ denote the number of pixels with grayscale value i. The total number of pixels in the image is then given by:

(17) $$N=\sum_{i=0}^{L-1}n_{i}$$

where $P_{i}=n_{i}/N$ denotes the probability that a pixel has grayscale value i.

When the threshold number is set to k, and the grayscale value t is chosen as the segmentation threshold, the image is divided into two regions: the target region with grayscale values in the range [0,t] and the background region with grayscale values in the range [t+1,L−1]. Let the proportion of pixels in the target region be $\omega_{0}$, with an average grayscale value $\mu_{0}$; the proportion of pixels in the background region be $\omega_{1}$, with an average grayscale value $\mu_{1}$; and the overall image average grayscale value be μ. The between-class variance v(t) is calculated as follows [46]:

(18) ω0=∑i=0tPiμ0=∑i=0tiPiω0ω1=∑i=t+1L−1Piμ1=∑i=t+1L−1iPiω1μ=∑i=0L−1iPiv(t)=ω0(μ0−μ)2+ω1(μ1−μ)2=ω0ω1(μ0−μ1)2

The optimal threshold $t_{best}$ is calculated using the following Formula (18):

(19) $$t_{\mathrm{best}\;}=\underset{0\leq t\leq L-1}{\arg max}\left[v(t)\right]$$

For multiple thresholds, the formula for the between-class variance is extended as follows:

(20) v(t1,t2,…,tk)=ω0ω1(μ0−μ1)2+ω0ω2(μ0−μ2)2+…+ω0ωk(μ0−μk)2+ω1ω2(μ1−μ2)2+…+ω1ω3(μ1−μ3)2+…+ωk−1ωk(μk−1−μk)2

where the formulas for $\mu_{i}$ and $\omega_{i}$ are given by:

(21) ωi−1=∑i=ti−1+1tiPi,1≤i≤k+1μi−1=∑i=ti−1+1tiiPiωi−1,1≤i≤k+1

The expression for the multi-level optimal threshold $T_{best}$ is given by:

(22) $$T_{best}=\underset{0\leq t_{1}\leq t_{2}\leq \ldots \leq t_{k}}{\arg max}[v(t_{1},t_{2},\ldots ,t_{k})]$$

**Figure 11:**
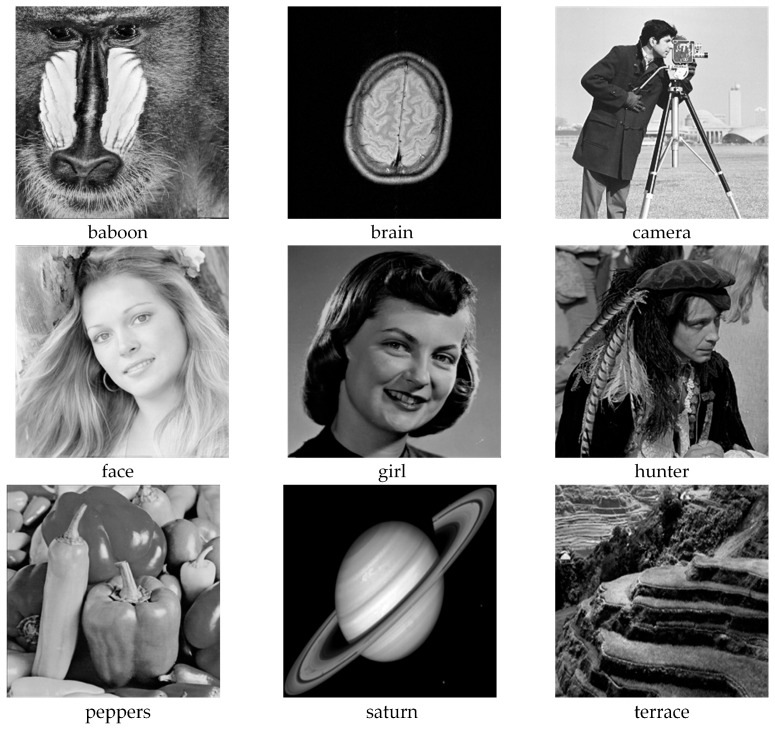
The set of benchmark images.

In this study, comparative experiments are conducted to evaluate the performance of the MFISOA in multi-level image thresholding segmentation tasks. Nine improved population-based intelligent algorithms are selected as comparison subjects. All algorithms are configured with the same population size (N = 30) and maximum number of iterations (T = 25), using the Otsu function as the unified objective evaluation criterion. The segmentation results at four different threshold levels (4, 6, 8, and 10 thresholds) were tested on nine benchmark images with different styles, as shown in Figure 11 [22]. To comprehensively assess the accuracy and robustness of the algorithms, each experiment was independently run 30 times, recording the standard deviation (Std) and average value (Ave) of the objective function. The parameter settings for all algorithms are consistent with those in previous studies to ensure the fairness of the experimental comparison.

### 4.1. Evaluation Metrics

In image segmentation performance evaluation, commonly used metrics include the Structural Similarity Index (SSIM), Peak Signal-to-Noise Ratio (PSNR), and Feature Similarity Index (FSIM). Higher SSIM and PSNR values indicate less distortion and better quality of the segmented image, while a higher FSIM value indicates lower segmentation error. The specific calculation methods for each of these metrics are as follows [43,47]:

The formula for PSNR is as follows:

(23) $$PSNR=10{log}_{10}(\frac{255^{2}}{MSE})$$

where MSE represents the Mean Squared Error between the original image I and the segmented image $I^{\prime }$, and is defined as:

(24) $$MSE=\frac{1}{MN}\sum_{j=1}^{M}\;\sum_{k=1}^{N}{\left[I(j,k)-I^{\prime }(j,k)\right]}^{2}$$

In the equation, M×N denotes the image dimensions, and $I\left(j,k\right)\;$and $I^{\prime }(j,k)$ represent the grayscale values of the original image and the segmented image at the pixel location (j,k), respectively.

SSIM is used to evaluate the similarity between two images in terms of brightness, contrast, and structural information. Its expression is given by:

(25) $$SSIM(I,I^{\prime })=\frac{\left(2\mu_{I}\mu_{I^{\prime }}+C_{1})(2\sigma_{{II}^{\prime }}+C_{2}\right)}{\left(\mu_{I}^{2}+\mu_{I^{\prime }}^{2}+C_{1})(\sigma_{I}^{2}+\sigma_{I^{\prime }}^{2}+C_{2}\right)}$$

where $\mu_{I}$ and $\mu_{I^{\prime }}$ represent the mean grayscale values of the original image I and the segmented image $I^{\prime }$, respectively, $\sigma_{{II}^{\prime }}$ is the covariance of the grayscale values between the original image I and the segmented image $I^{\prime }$, and $\sigma_{I}^{2}$ and $\sigma_{I^{\prime }}^{2}$ are the variances of the original and segmented images, respectively. The constants are generally set as $C_{1}=K_{1}L$, $C_{2}={\left(K_{2}L\right)}^{2}$, where $K_{1}=0.01$, $K_{2}=0.03$, and L is the maximum grayscale level.

The value range of SSIM is [−1, 1], where a value of 1 indicates that the two images are identical. This metric simulates the human visual system by evaluating image distortion from three dimensions: brightness, contrast, and structure. Brightness is estimated by the mean, contrast is measured by the standard deviation, and structural information is reflected by the covariance. In segmentation tasks, the closer the SSIM value is to 1, the better the structural preservation and the higher the segmentation quality [48,49].

The formula for the FSIM is as follows:

(26) $$FSIM(I,I^{\prime })=\frac{\sum_{x\in \Omega }S_{L}(x)\cdot PC_{m}(x)}{\sum_{x\in \Omega }PC_{m}(x)}$$

The phase congruency PC(x) is defined as:

(27) PC(x)=E(x)ε+∑nAn(x)An(x)=en(x)2+on(x)2E(x)=∑nen(x)2+∑non(x)2

where ε is a small constant, x denotes the pixel location, and Ω represents the entire image domain. Here, $e_{n}(x)$ and $o_{n}(x)$ denote the even- and odd-symmetric filter response components at scale n, respectively, while E(x) denotes the local energy at pixel x. The phase congruency at pixel x is defined as $PC_{m}(x)=max(PC_{1}(x),PC_{2}(x))$, where $PC_{1}(x)$ and $PC_{2}(x)$ are the phase congruency values of the original image and the segmented image, respectively.

### 4.2. Analysis of Otsu Results Based on MFISOA

In this study, the MFISOA is employed with the Otsu criterion as the objective function to conduct multi-level thresholding experiments on nine selected benchmark images. The evaluation phase utilizes the maximum value of the Otsu objective function, together with PSNR, FSIM, and SSIM, as key performance indicators. Higher values for these metrics indicate better segmentation accuracy and robustness of the algorithm. The experimental results demonstrate that, at four threshold levels (4, 6, 8, and 10), MFISOA significantly outperforms the other compared algorithms (PSO, MFO, GWO, AVOA, AOA, INFO, DBO, BPBO, SOA) across all metrics.

Table 8 presents the distribution of the optimal threshold values selected by MFISOA across the histograms of nine images, clearly highlighting the differences in threshold selection for the same image at different threshold levels. The thresholds selected by MFISOA are more aligned with the peak intervals of the image grayscale distribution, thereby providing a solid basis for high-quality segmentation. The detailed quantitative comparison results in terms of Otsu fitness, SSIM, FSIM, and PSNR are provided in the Supplementary Materials (Tables S1–S4). Overall, these results show that MFISOA consistently achieves competitive segmentation performance in both accuracy and stability across different images and threshold levels. For example, in the four-threshold segmentation of the “camera” image, MFISOA attains a PSNR value of 19.8198, representing a 2.53% improvement over the standard SOA with a PSNR of 19.3303, which effectively alleviates the segmentation blur in overlapping grayscale regions. All experiments were independently conducted over 30 runs to ensure statistical reliability, and the parameters of all algorithms, such as population size and maximum iteration count, were kept consistent to ensure a fair comparison.

The detailed optimal threshold-selection results of MFISOA for the nine images under different threshold levels are provided in Table S1 of the Supplementary Materials, further demonstrating the algorithm’s ability to capture the grayscale distribution characteristics of the images. For the brain medical image with distinct grayscale layers, the thresholds for 4-level segmentation are concentrated in the 50–120 intensity range, accurately matching the grayscale differences between brain tissue and cerebrospinal fluid, thereby laying a foundation for subsequent lesion localization. For the Saturn image with ring-like texture features, the 8-level segmentation thresholds can completely separate the grayscale layers of the planetary core, rings, and cosmic background, avoiding the ring discontinuity caused by threshold shifts in the conventional SOA. For the baboon image with severe grayscale overlap and complex textures, the 10-level segmentation thresholds span the full 0–255 intensity range with adjacent gaps of 8–15 levels, effectively distinguishing hair, skin, and background regions. This benefit is attributed to MFISOA’s differential evolution-driven exploitation strategy, which generates directional perturbations based on inter-individual differences in the population and can precisely locate the optimal combination in the high-dimensional threshold space, thereby preventing over-segmentation or under-segmentation due to threshold clustering.

The detailed quantitative results of Otsu fitness, SSIM, FSIM, and PSNR are provided in the Supplementary Materials (Tables S2–S4). Overall, these results quantitatively validate the segmentation performance of MFISOA across different images and threshold levels. In terms of the Otsu objective, MFISOA achieves the highest average values across all images and threshold levels. For example, in 4-level segmentation of the camera image, its Ave reaches 4.60 × 10^3^, consistent with the theoretically optimal inter-class variance and higher than those of standard SOA (4.58 × 10^3^) and PSO (4.59 × 10^3^), indicating that MFISOA can identify threshold combinations that maximize inter-class separability. Moreover, MFISOA yields markedly smaller Std values than the competing algorithms. For instance, in 6-level segmentation of the face image, MFISOA attains an Std of only 1.40 × 10^0^, substantially lower than SOA (4.18 × 10^0^) and AOA (1.45 × 10^1^). This robustness is attributed to the centroid-based opposition learning boundary-control strategy, which preserves population diversity and mitigates search fluctuations caused by local optima. MFISOA also performs strongly on visual-quality metrics. In 8-level segmentation of the brain image, MFISOA achieves SSIM = 0.6812, FSIM = 0.9421, and PSNR = 28.8279 dB, representing improvements of 3.2%, 4.5%, and 5.1% over SOA, thus better preserving anatomical integrity. For the camera image under 4-level segmentation, MFISOA attains a PSNR of 19.6292 dB, a 5.08% gain over SOA (18.6798 dB), effectively reducing edge distortions. As the threshold level increases to 10 in high-dimensional settings, MFISOA’s advantage becomes more pronounced: for the terrace image, it achieves the best SSIM (0.8859), FSIM (0.9500), and PSNR (27.2994 dB), whereas algorithms such as DBO and BPBO degrade due to the enlarged search space, confirming the efficiency of MFISOA’s adaptive cooperative foraging strategy in high-dimensional threshold optimization.

**Figure 12:**
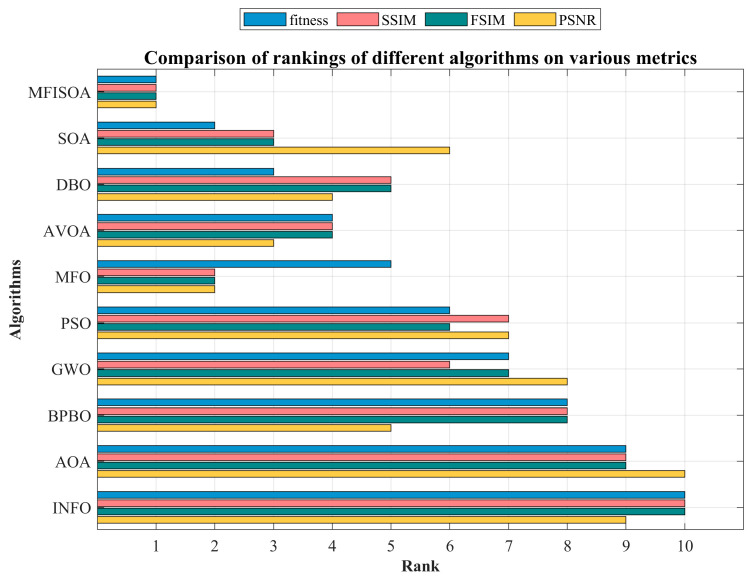
Final ranking of different algorithms across various metrics.

Figure 12 visualizes the comprehensive ranking of the compared algorithms, showing that MFISOA consistently ranks first across fitness, SSIM, FSIM, and PSNR (Final-Rank = 1), significantly outperforming SOA (2nd) and DBO (3rd). Most baseline methods exhibit metric trade-offs; for instance, BPBO ranks third in PSNR but drops to fifth in FSIM, while INFO maintains relatively balanced rankings yet remains inferior to MFISOA on all metrics. MFISOA’s overall superiority stems from the synergy of three strategies: the adaptive cooperative foraging strategy enhances global exploration, the differential evolution-driven exploitation strategy improves local threshold refinement, and the centroid-based opposition learning boundary-control strategy stabilizes the search process. Together, these mechanisms achieve a balance between “statistical optimality” (maximizing the Otsu criterion) and “visual optimality” (high SSIM/FSIM/PSNR), which is difficult to attain with single-strategy variants.

Overall, the reasonable threshold distributions provided in Table 8 lay the foundation for high-quality segmentation, while the detailed quantitative results in the Supplementary Materials (Tables S1–S4) further verify the accuracy and robustness of MFISOA. In addition, the comprehensive ranking shown in Figure 12 confirms the overall advantage of MFISOA. Together, these results form a coherent evidence chain, demonstrating that the proposed multi-strategy fusion design can be effectively transferred from numerical optimization to multi-threshold image segmentation.

## 5. Summary and Prospect

This paper addresses the core deficiencies of the standard SOA in global exploration, local exploitation, and population diversity maintenance by proposing MFISOA. The algorithm constructs a comprehensive optimization system through three core strategies: an adaptive collaborative foraging strategy based on individual quality, which dynamically allocates search resources according to fitness differences among individuals, thus resolving the issue of rigid exploration strategies in the standard SOA; a exploitation strategy driven by differential evolution, which generates directional perturbations using individual differences within the population to refine the local search process; and a centroid-based reverse learning boundary control strategy, where the population centroid serves as a reference for reverse mapping of out-of-bounds individuals, effectively preserving population diversity.

In numerical optimization experiments, using the CEC2017 and CEC2022 benchmark test sets, MFISOA is compared with nine advanced algorithms, including PSO, GWO, and SOA. Through ablation studies, convergence curve analysis, box plot analysis, as well as statistical validation such as Wilcoxon rank-sum tests and Friedman mean rank tests, it is confirmed that MFISOA demonstrates superior optimization accuracy, convergence speed, and stability in unimodal, multimodal, hybrid, and high-dimensional composite functions, with significant advantages in high-dimensional and complex scenarios. In multi-threshold image segmentation applications, using the Otsu criterion as the objective function, MFISOA outperforms comparison algorithms in key metrics such as between-class variance, SSIM, FSIM, and PSNR across the 4, 6, 8, and 10 threshold levels of 9 benchmark images. MFISOA is able to accurately capture image grayscale distribution features, achieving high-quality region segmentation and providing a reliable optimization tool for practical image segmentation tasks.

Although MFISOA has achieved good results in numerical optimization and multi-threshold image segmentation, there is still room for further improvement, and the following research directions are proposed for future work. First, the algorithm’s parameter settings still rely on empirical values. Currently, parameters such as the Levy flight parameter β and differential evolution-related parameters are fixed, and no systematic sensitivity analysis has been conducted to evaluate their influence on algorithm performance under different problem settings. Future work could combine parameter sensitivity analysis with reinforcement learning or adaptive parameter adjustment strategies to dynamically adjust parameters according to the optimization stage and problem characteristics, further enhancing the algorithm’s adaptability to different problem types. Second, the algorithm still has room for optimization in terms of computational complexity when handling large-scale high-dimensional optimization problems. As the number of thresholds or the problem’s dimensionality increases, the computational cost of population iteration rises. Future work could explore clustering strategies or parallel computing mechanisms to reduce time overhead while ensuring optimization effectiveness. Furthermore, the image segmentation evaluation system could be further enriched. This study mainly uses objective quantitative metrics for evaluation, but future work could incorporate human visual perception characteristics, introducing subjective evaluation dimensions. The application of MFISOA could also be expanded to more complex real-world scenarios, such as medical image segmentation, multi-target remote sensing image segmentation, and others, to validate its segmentation performance in challenging conditions such as special textures, strong noise, and multimodal images. Finally, the algorithm’s fusion strategies could be further expanded. The collaborative mechanisms of the three core strategies still have room for improvement. Future work could attempt to integrate more efficient local search operators or global exploration strategies, as well as explore the integration of MFISOA with deep learning models, providing stronger technical support for more complex image segmentation tasks.

## Figures and Tables

**Table 1:** Comparison of the strengths and limitations of representative methods for multilevel threshold image segmentation.

Method Category	Main Idea	Major Strengths	Major Limitations
Particle Swarm Optimization (PSO) [23]	Multilevel threshold search through collaborative particle updating	Simple structure, easy implementation, fast convergence	Prone to premature convergence in high-dimensional search; insufficient population diversity
Whale Optimization Algorithm (WOA) [25]	Search based on encircling prey and spiral updating mechanisms	Stable search process, strong local exploitation capability	Limited global exploration range; may miss promising thresholds
Dung Beetle Optimizer (DBO) [27]	Diversity-enhanced search through behavior-inspired mechanisms	Strong exploration ability; competitive on some multimodal problems	Limited stability in high-dimensional multilevel thresholding and noisy scenarios
Standard Seagull Optimization Algorithm (SOA) [30]	Optimization based on migration, aggregation, and attack behaviors of seagulls	Balances global exploration and local exploitation to some extent; concise model	Rigid exploration strategy, insufficient local exploitation precision, and coarse boundary control
Hybrid SOA-based methods [32]	Combination of SOA with other operators for performance improvement	Enhances local exploitation ability or overall optimization accuracy	Mostly loosely hybridized; difficult to balance exploration, exploitation, and diversity preservation simultaneously
Proposed MFISOA	Adaptive cooperative foraging, DE-driven exploitation, and centroid opposition-based boundary control	Improves exploration guidance, local exploitation precision, and population diversity preservation	More complex algorithmic structure with additional computational operations

**Table 2:** Parameter settings of the compared algorithms.

Algorithms	Name of the Parameter	Value of the Parameter
PSO	$C_{1},C_{2},w$	1.49115, 1.49445, 0.9
MFO	a,b	[−2, 1], 1
GWO	a	[0,2]
AVOA	L1,L2,w,p1,p2,p3	0.8, 0.2, 2.5, 0.6, 0.4, 0.6
AOA	α,μ	5, 0.5
INFO	c,d	2, 4
DBO	$P_{percent}$	0.2
BPBO	$P_{i}$	0.7
SOA	$A,f_{c}$	[2,0], 2
MFISOA	$A,f_{c},F,CR,\beta$	[2,0], 2, 0.5, 0.9, 1.5

**Table 3:** Experimental results of CEC2022 (dim = 10).

Function	Metric	PSO	MFO	GWO	AVOA	AOA	INFO	DBO	BPBO	SOA	MFISOA
F1	Ave	4.2418 × 10^2^	5.9894 × 10^3^	1.3871 × 10^3^	4.4645 × 10^2^	1.1654 × 10^4^	3.0000 × 10^2^	1.5692 × 10^3^	3.3686 × 10^2^	5.0504 × 10^3^	**3.0000 × 10^2^**
	Std	5.0699 × 10^1^	7.7516 × 10^3^	1.5906 × 10^3^	2.1029 × 10^2^	4.3149 × 10^3^	1.2271 × 10^−^^6^	1.9889 × 10^3^	4.4551 × 10^1^	1.8968 × 10^3^	**3.3548 × 10^−8^**
F2	Ave	4.2965 × 10^2^	4.1569 × 10^2^	4.2644 × 10^2^	4.2840 × 10^2^	1.3780 × 10^3^	**4.0758 × 10^2^**	4.4621 × 10^2^	4.1018 × 10^2^	6.4498 × 10^2^	4.1199 × 10^2^
	Std	3.0289 × 10^1^	1.6044 × 10^1^	2.1677 × 10^1^	3.1049 × 10^1^	7.7385 × 10^2^	**1.3281 × 10^1^**	6.8747 × 10^1^	2.1908 × 10^1^	2.0151 × 10^2^	2.3675 × 10^1^
F3	Ave	6.0233 × 10^2^	6.0343 × 10^2^	6.0285 × 10^2^	6.2667 × 10^2^	6.4157 × 10^2^	6.0152 × 10^2^	6.1196 × 10^2^	6.1555 × 10^2^	6.2735 × 10^2^	**6.0000 × 10^2^**
	Std	1.4208 × 10^0^	4.1954 × 10^0^	2.4094 × 10^0^	1.3872 × 10^1^	7.4419 × 10^0^	3.6324 × 10^0^	7.7331 × 10^0^	8.6649 × 10^0^	7.6605 × 10^0^	**2.2064 × 10^−4^**
F4	Ave	8.2258 × 10^2^	8.3436 × 10^2^	8.1968 × 10^2^	8.3082 × 10^2^	8.3946 × 10^2^	8.2220 × 10^2^	8.3533 × 10^2^	8.1934 × 10^2^	8.1971 × 10^2^	**8.1781 × 10^2^**
	Std	6.8612 × 10^0^	1.4379 × 10^1^	1.1193 × 10^1^	9.8317 × 10^0^	9.0389 × 10^0^	8.1390 × 10^0^	1.1642 × 10^1^	**5.6252 × 10^0^**	6.3787 × 10^0^	7.3171 × 10^0^
F5	Ave	9.0460 × 10^2^	1.1122 × 10^3^	9.2489 × 10^2^	1.2904 × 10^3^	1.3946 × 10^3^	9.4039 × 10^2^	9.5574 × 10^2^	9.4650 × 10^2^	1.0561 × 10^3^	**9.0002 × 10^2^**
	Std	3.1218 × 10^0^	3.2169 × 10^2^	3.9564 × 10^1^	2.1809 × 10^2^	1.8799 × 10^2^	5.1662 × 10^1^	5.7902 × 10^1^	3.6306 × 10^1^	7.1295 × 10^1^	**8.3988 × 10^−2^**
F6	Ave	7.2131 × 10^3^	4.5685 × 10^3^	8.6533 × 10^3^	4.5051 × 10^3^	1.0441 × 10^8^	1.9913 × 10^3^	4.7347 × 10^3^	3.4416 × 10^3^	2.7505 × 10^3^	**1.8029 × 10^3^**
	Std	3.9117 × 10^3^	2.1568 × 10^3^	3.3522 × 10^3^	2.3242 × 10^3^	1.9995 × 10^8^	4.7362 × 10^2^	2.2601 × 10^3^	1.7212 × 10^3^	1.0410 × 10^3^	**1.0853 × 10^0^**
F7	Ave	2.0242 × 10^3^	2.0348 × 10^3^	2.0369 × 10^3^	2.0460 × 10^3^	2.0978 × 10^3^	2.0230 × 10^3^	2.0410 × 10^3^	2.0464 × 10^3^	2.0552 × 10^3^	**2.0016 × 10^3^**
	Std	6.1274 × 10^0^	1.7281 × 10^1^	1.2926 × 10^1^	1.9265 × 10^1^	2.6070 × 10^1^	1.0945 × 10^1^	1.6891 × 10^1^	1.9175 × 10^1^	1.9959 × 10^1^	**3.6989 × 10^0^**
F8	Ave	2.2502 × 10^3^	2.2239 × 10^3^	2.2245 × 10^3^	2.2267 × 10^3^	2.2909 × 10^3^	2.2190 × 10^3^	2.2354 × 10^3^	2.2281 × 10^3^	2.2238 × 10^3^	**2.2117 × 10^3^**
	Std	4.9498 × 10^1^	6.9328 × 10^0^	8.0249 × 10^0^	5.4340 × 10^0^	7.4348 × 10^1^	5.8480 × 10^0^	3.0856 × 10^1^	**3.9167 × 10^0^**	4.3619 × 10^0^	6.7600 × 10^0^
F9	Ave	2.5302 × 10^3^	2.5416 × 10^3^	2.5695 × 10^3^	2.5410 × 10^3^	2.7416 × 10^3^	2.5342 × 10^3^	2.5636 × 10^3^	2.5346 × 10^3^	2.6846 × 10^3^	**2.5293 × 10^3^**
	Std	3.7035 × 10^0^	3.4960 × 10^1^	3.1541 × 10^1^	1.4778 × 10^1^	5.2775 × 10^1^	2.6826 × 10^1^	4.8287 × 10^1^	2.6814 × 10^1^	2.8210 × 10^1^	**5.4158 × 10^−10^**
F10	Ave	2.6200 × 10^3^	2.5594 × 10^3^	2.5521 × 10^3^	2.5594 × 10^3^	2.7439 × 10^3^	2.5909 × 10^3^	2.5469 × 10^3^	2.5429 × 10^3^	2.5746 × 10^3^	**2.5004 × 10^3^**
	Std	1.4705 × 10^2^	1.2253 × 10^2^	6.0285 × 10^1^	6.8252 × 10^1^	1.8686 × 10^2^	7.5966 × 10^1^	6.6431 × 10^1^	6.0587 × 10^1^	6.4373 × 10^1^	**9.3375 × 10^−2^**
F11	Ave	2.8288 × 10^3^	2.8441 × 10^3^	2.8398 × 10^3^	2.7644 × 10^3^	3.6169 × 10^3^	2.7296 × 10^3^	2.8285 × 10^3^	2.6725 × 10^3^	2.9760 × 10^3^	**2.6100 × 10^3^**
	Std	1.8134 × 10^2^	1.6935 × 10^2^	1.7182 × 10^2^	1.6444 × 10^2^	5.7227 × 10^2^	1.4948 × 10^2^	2.2699 × 10^2^	1.1767 × 10^2^	2.6527 × 10^2^	**3.8165 × 10^1^**
F12	Ave	2.8723 × 10^3^	**2.8636 × 10^3^**	2.8658 × 10^3^	2.8698 × 10^3^	3.0373 × 10^3^	2.8644 × 10^3^	2.8714 × 10^3^	2.8665 × 10^3^	2.9600 × 10^3^	2.8644 × 10^3^
	Std	1.2575 × 10^1^	**1.3479 × 10^0^**	4.9141 × 10^0^	6.8943 × 10^0^	7.0731 × 10^1^	1.4638 × 10^0^	1.0387 × 10^1^	2.0234 × 10^0^	3.9356 × 10^1^	1.3767 × 10^0^

**Table 4:** Experimental results of CEC2022 (dim = 20).

Function	Metric	PSO	MFO	GWO	AVOA	AOA	INFO	DBO	BPBO	SOA	MFISOA
F1	Ave	6.5266 × 10^3^	4.4260 × 10^4^	1.4417 × 10^4^	1.8229 × 10^4^	3.3406 × 10^4^	1.2158 × 10^3^	3.6136 × 10^4^	1.2296 × 10^4^	2.4444 × 10^4^	**4.0590 × 10^2^**
	Std	3.7495 × 10^3^	1.7306 × 10^4^	4.8837 × 10^3^	5.6504 × 10^3^	9.9027 × 10^3^	1.0399 × 10^3^	1.0522 × 10^4^	5.7399 × 10^3^	5.5381 × 10^3^	**1.0062 × 10^2^**
F2	Ave	4.9195 × 10^2^	5.3603 × 10^2^	5.1666 × 10^2^	5.1169 × 10^2^	2.4431 × 10^3^	4.5712 × 10^2^	5.1787 × 10^2^	4.8161 × 10^2^	1.3350 × 10^3^	**4.4650 × 10^2^**
	Std	5.1925 × 10^1^	1.2585 × 10^2^	3.7120 × 10^1^	5.0899 × 10^1^	8.0990 × 10^2^	**1.4438 × 10^1^**	8.1237 × 10^1^	2.3342 × 10^1^	3.6301 × 10^2^	1.8415 × 10^1^
F3	Ave	6.1165 × 10^2^	6.2232 × 10^2^	6.0888 × 10^2^	6.5007 × 10^2^	6.6855 × 10^2^	6.1129 × 10^2^	6.3296 × 10^2^	6.3974 × 10^2^	6.5358 × 10^2^	**6.0012 × 10^2^**
	Std	3.4392 × 10^0^	8.9330 × 10^0^	3.4301 × 10^0^	1.2792 × 10^1^	7.8261 × 10^0^	6.4897 × 10^0^	1.1704 × 10^1^	1.3931 × 10^1^	5.2087 × 10^0^	**8.6665 × 10^−2^**
F4	Ave	9.0969 × 10^2^	8.9679 × 10^2^	8.8104 × 10^2^	8.8824 × 10^2^	9.5599 × 10^2^	**8.5825 × 10^2^**	9.1399 × 10^2^	8.7685 × 10^2^	8.9032 × 10^2^	9.0458 × 10^2^
	Std	1.6742 × 10^1^	2.8056 × 10^1^	2.9222 × 10^1^	2.1237 × 10^1^	1.6213 × 10^1^	1.7336 × 10^1^	3.0620 × 10^1^	1.4845 × 10^1^	**1.2715 × 10^1^**	3.6115 × 10^1^
F5	Ave	1.0184 × 10^3^	2.6991 × 10^3^	1.3817 × 10^3^	2.3065 × 10^3^	3.1400 × 10^3^	1.5012 × 10^3^	2.0272 × 10^3^	2.1630 × 10^3^	1.9603 × 10^3^	**9.0265 × 10^2^**
	Std	7.2853 × 10^1^	7.3483 × 10^2^	3.7845 × 10^2^	4.2122 × 10^2^	4.5444 × 10^2^	3.4405 × 10^2^	6.7850 × 10^2^	5.6865 × 10^2^	2.0389 × 10^2^	**6.4866 × 10^0^**
F6	Ave	1.5865 × 10^6^	7.3868 × 10^6^	8.8226 × 10^6^	6.2714 × 10^3^	1.2659 × 10^9^	5.7913 × 10^3^	1.0151 × 10^6^	4.7029 × 10^3^	1.8033 × 10^8^	**1.8830 × 10^3^**
	Std	1.0840 × 10^6^	1.5468 × 10^7^	1.6065 × 10^7^	4.5667 × 10^3^	1.0634 × 10^9^	4.4583 × 10^3^	2.1253 × 10^6^	2.9175 × 10^3^	2.0965 × 10^8^	**8.7233 × 10^1^**
F7	Ave	2.1044 × 10^3^	2.1314 × 10^3^	2.0885 × 10^3^	2.1492 × 10^3^	2.2253 × 10^3^	2.1140 × 10^3^	2.1318 × 10^3^	2.1404 × 10^3^	2.1311 × 10^3^	**2.0388 × 10^3^**
	Std	3.2432 × 10^1^	6.9717 × 10^1^	3.6572 × 10^1^	6.8309 × 10^1^	7.2445 × 10^1^	5.2204 × 10^1^	4.4458 × 10^1^	3.2393 × 10^1^	1.8114 × 10^1^	**1.3183 × 10^1^**
F8	Ave	2.3040 × 10^3^	2.2469 × 10^3^	2.2460 × 10^3^	2.2533 × 10^3^	2.5582 × 10^3^	2.2627 × 10^3^	2.2947 × 10^3^	2.2897 × 10^3^	2.2539 × 10^3^	**2.2300 × 10^3^**
	Std	7.8924 × 10^1^	2.9999 × 10^1^	3.3329 × 10^1^	3.1942 × 10^1^	2.9196 × 10^2^	5.7730 × 10^1^	6.1372 × 10^1^	6.1067 × 10^1^	5.3193 × 10^1^	**7.7320 × 10^0^**
F9	Ave	2.5024 × 10^3^	2.5019 × 10^3^	2.5115 × 10^3^	2.4927 × 10^3^	3.2946 × 10^3^	**2.4808 × 10^3^**	2.5092 × 10^3^	2.4856 × 10^3^	2.7970 × 10^3^	2.4808 × 10^3^
	Std	3.2459 × 10^1^	2.2391 × 10^1^	1.9268 × 10^1^	8.3693 × 10^0^	3.1834 × 10^2^	**4.6008 × 10^−^^5^**	2.9564 × 10^1^	7.6700 × 10^0^	1.2322 × 10^2^	7.9485 × 10^−3^
F10	Ave	4.0360 × 10^3^	3.9947 × 10^3^	3.7264 × 10^3^	3.7062 × 10^3^	6.0602 × 10^3^	3.1126 × 10^3^	3.0738 × 10^3^	3.5925 × 10^3^	3.3698 × 10^3^	**2.5260 × 10^3^**
	Std	9.0484 × 10^2^	1.0816 × 10^3^	1.4474 × 10^3^	7.8885 × 10^2^	6.6171 × 10^2^	6.2019 × 10^2^	9.8757 × 10^2^	1.1416 × 10^3^	9.3581 × 10^2^	**1.0466 × 10^2^**
F11	Ave	3.4377 × 10^3^	3.9441 × 10^3^	3.7335 × 10^3^	2.9592 × 10^3^	8.2035 × 10^3^	**2.9371 × 10^3^**	3.3679 × 10^3^	2.9923 × 10^3^	7.1729 × 10^3^	2.9433 × 10^3^
	Std	3.9249 × 10^2^	7.5881 × 10^2^	4.8008 × 10^2^	1.5435 × 10^2^	9.6155 × 10^2^	2.7729 × 10^2^	8.8275 × 10^2^	8.3453 × 10^1^	6.8064 × 10^2^	**5.0400 × 10^1^**
F12	Ave	2.9919 × 10^3^	2.9654 × 10^3^	2.9732 × 10^3^	3.0190 × 10^3^	3.8919 × 10^3^	2.9853 × 10^3^	3.0559 × 10^3^	3.0196 × 10^3^	3.4430 × 10^3^	**2.9631 × 10^3^**
	Std	3.8632 × 10^1^	2.2459 × 10^1^	**1.9932 × 10^1^**	6.1791 × 10^1^	2.4990 × 10^2^	3.8315 × 10^1^	7.1033 × 10^1^	5.2983 × 10^1^	1.0427 × 10^2^	2.0007 × 10^1^

**Table 5:** Experimental results of CEC2017 (dim = 30).

Function	Metric	PSO	MFO	GWO	AVOA	AOA	INFO	DBO	BPBO	SOA	MFISOA
F1	Ave	1.4290 × 10^9^	9.8427 × 10^9^	4.1070 × 10^9^	9.2687 × 10^6^	5.0731 × 10^10^	7.7858 × 10^4^	2.3589 × 10^8^	2.9160 × 10^7^	3.6880 × 10^10^	**4.3204 × 10^3^**
	Std	9.7541 × 10^8^	7.4074 × 10^9^	2.3676 × 10^9^	2.2219 × 10^7^	1.2712 × 10^10^	3.2750 × 10^5^	1.4978 × 10^8^	2.1277 × 10^7^	6.5467 × 10^9^	**3.4297 × 10^3^**
F2	Ave	2.5778 × 10^30^	6.6826 × 10^38^	2.1569 × 10^33^	2.1648 × 10^24^	3.4802 × 10^49^	7.8109 × 10^23^	2.0486 × 10^34^	2.3373 × 10^22^	3.5927 × 10^42^	**1.2968 × 10^18^**
	Std	9.8135 × 10^30^	3.0548 × 10^39^	5.2028 × 10^33^	8.9844 × 10^24^	1.0719 × 10^50^	3.8580 × 10^24^	7.0904 × 10^34^	4.9972 × 10^22^	1.3969 × 10^43^	**5.4671 × 10^18^**
F3	Ave	6.7169 × 10^4^	1.8504 × 10^5^	6.1671 × 10^4^	5.7104 × 10^4^	7.8162 × 10^4^	1.4473 × 10^4^	8.9675 × 10^4^	5.2121 × 10^4^	7.4910 × 10^4^	**1.4234 × 10^4^**
	Std	2.0163 × 10^4^	5.1022 × 10^4^	1.0424 × 10^4^	1.0971 × 10^4^	1.1056 × 10^4^	**4.5916 × 10^3^**	2.5148 × 10^4^	7.4581 × 10^3^	8.4888 × 10^3^	6.3981 × 10^3^
F4	Ave	6.6668 × 10^2^	1.1369 × 10^3^	6.8880 × 10^2^	5.4669 × 10^2^	1.4455 × 10^4^	5.1091 × 10^2^	6.3731 × 10^2^	5.3747 × 10^2^	8.0092 × 10^3^	**4.9251 × 10^2^**
	Std	1.5681 × 10^2^	6.4198 × 10^2^	8.1418 × 10^1^	3.5383 × 10^1^	4.0651 × 10^3^	3.0572 × 10^1^	8.7994 × 10^1^	2.6621 × 10^1^	1.7190 × 10^3^	**2.3898 × 10^1^**
F5	Ave	7.0578 × 10^2^	7.2179 × 10^2^	6.5204 × 10^2^	7.3840 × 10^2^	8.9346 × 10^2^	6.4609 × 10^2^	7.6198 × 10^2^	7.2894 × 10^2^	7.7610 × 10^2^	**6.4361 × 10^2^**
	Std	**2.5832 × 10^1^**	5.6949 × 10^1^	5.4005 × 10^1^	4.7199 × 10^1^	3.5022 × 10^1^	3.4534 × 10^1^	5.5929 × 10^1^	3.7528 × 10^1^	3.8506 × 10^1^	7.0647 × 10^1^
F6	Ave	6.2021 × 10^2^	6.3886 × 10^2^	6.1667 × 10^2^	6.5631 × 10^2^	6.8161 × 10^2^	6.2630 × 10^2^	6.5108 × 10^2^	6.5525 × 10^2^	6.5964 × 10^2^	**6.0247 × 10^2^**
	Std	5.2380 × 10^0^	1.0855 × 10^1^	4.7685 × 10^0^	6.8140 × 10^0^	6.4767 × 10^0^	9.4538 × 10^0^	1.3427 × 10^1^	1.0261 × 10^1^	4.9384 × 10^0^	**1.5397 × 10^0^**
F7	Ave	9.9939 × 10^2^	1.1204 × 10^3^	9.5250 × 10^2^	1.1757 × 10^3^	1.3962 × 10^3^	1.0061 × 10^3^	1.0228 × 10^3^	1.1572 × 10^3^	1.2249 × 10^3^	**9.1599 × 10^2^**
	Std	**2.4035 × 10^1^**	1.4946 × 10^2^	5.9521 × 10^1^	9.0619 × 10^1^	7.4188 × 10^1^	8.0421 × 10^1^	7.1705 × 10^1^	9.9037 × 10^1^	5.8031 × 10^1^	6.2592 × 10^1^
F8	Ave	1.0115 × 10^3^	1.0067 × 10^3^	**9.2564 × 10^2^**	9.7007 × 10^2^	1.1272 × 10^3^	9.3246 × 10^2^	1.0411 × 10^3^	9.6711 × 10^2^	1.0262 × 10^3^	9.5117 × 10^2^
	Std	3.2239 × 10^1^	4.9294 × 10^1^	4.2927 × 10^1^	3.0330 × 10^1^	3.4399 × 10^1^	2.7488 × 10^1^	4.9210 × 10^1^	**2.4999 × 10^1^**	2.7001 × 10^1^	6.7854 × 10^1^
F9	Ave	2.0488 × 10^3^	7.1460 × 10^3^	3.0427 × 10^3^	5.3106 × 10^3^	7.9949 × 10^3^	2.9207 × 10^3^	6.9743 × 10^3^	6.5274 × 10^3^	5.0708 × 10^3^	**1.0083 × 10^3^**
	Std	9.3303 × 10^2^	2.4584 × 10^3^	7.7628 × 10^2^	7.7224 × 10^2^	1.2698 × 10^3^	7.8852 × 10^2^	2.2858 × 10^3^	1.4563 × 10^3^	6.5441 × 10^2^	**7.7328 × 10^1^**
F10	Ave	7.5090 × 10^3^	5.4350 × 10^3^	6.4884 × 10^3^	5.7650 × 10^3^	7.8064 × 10^3^	**4.9230 × 10^3^**	6.7773 × 10^3^	6.3307 × 10^3^	5.8827 × 10^3^	8.1473 × 10^3^
	Std	6.7346 × 10^2^	7.4857 × 10^2^	1.9161 × 10^3^	7.3556 × 10^2^	4.4128 × 10^2^	8.3201 × 10^2^	1.1225 × 10^3^	1.1531 × 10^3^	**3.2568 × 10^2^**	8.2802 × 10^2^
F11	Ave	1.5016 × 10^3^	5.0988 × 10^3^	2.5902 × 10^3^	1.3045 × 10^3^	9.4797 × 10^3^	1.2928 × 10^3^	1.8861 × 10^3^	1.3323 × 10^3^	5.1162 × 10^3^	**1.1920 × 10^3^**
	Std	8.4483 × 10^1^	4.0122 × 10^3^	1.1608 × 10^3^	6.3560 × 10^1^	2.7839 × 10^3^	6.1721 × 10^1^	8.8084 × 10^2^	6.4446 × 10^1^	1.3904 × 10^3^	**4.5711 × 10^1^**
F12	Ave	6.5256 × 10^7^	1.8957 × 10^8^	3.4846 × 10^8^	1.2230 × 10^7^	1.2877 × 10^10^	1.0480 × 10^6^	7.3512 × 10^7^	1.0067 × 10^7^	7.5598 × 10^9^	**1.0971 × 10^5^**
	Std	3.9958 × 10^7^	2.3909 × 10^8^	4.8845 × 10^8^	1.3554 × 10^7^	3.4097 × 10^9^	8.8989 × 10^5^	7.6438 × 10^7^	9.7308 × 10^6^	2.1900 × 10^9^	**5.8935 × 10^4^**
F13	Ave	8.3539 × 10^6^	1.3997 × 10^8^	8.5598 × 10^7^	1.2369 × 10^5^	1.3936 × 10^10^	2.3901 × 10^4^	6.3481 × 10^6^	1.8839 × 10^5^	2.5810 × 10^9^	**7.8689 × 10^3^**
	Std	1.7737 × 10^7^	5.5810 × 10^8^	1.1680 × 10^8^	8.0388 × 10^4^	6.5121 × 10^9^	2.2152 × 10^4^	1.5218 × 10^7^	3.4541 × 10^5^	2.0570 × 10^9^	**1.2321 × 10^4^**
F14	Ave	1.1946 × 10^5^	6.8354 × 10^5^	7.4846 × 10^5^	6.9024 × 10^5^	3.9551 × 10^6^	9.5570 × 10^3^	3.0534 × 10^5^	1.3013 × 10^5^	1.0194 × 10^6^	**1.5163 × 10^3^**
	Std	9.3243 × 10^4^	8.9233 × 10^5^	1.1445 × 10^6^	7.7073 × 10^5^	4.7651 × 10^6^	1.2879 × 10^4^	3.9496 × 10^5^	1.4681 × 10^5^	6.1486 × 10^5^	**1.3288 × 10^1^**
F15	Ave	2.2974 × 10^5^	3.0150 × 10^7^	4.5141 × 10^6^	4.7017 × 10^4^	2.8763 × 10^8^	1.2938 × 10^4^	1.0940 × 10^5^	1.7764 × 10^4^	1.8241 × 10^7^	**1.6842 × 10^3^**
	Std	2.7079 × 10^5^	1.6484 × 10^8^	1.1411 × 10^7^	3.1793 × 10^4^	3.8225 × 10^8^	1.2773 × 10^4^	1.3856 × 10^5^	9.3078 × 10^3^	4.5627 × 10^7^	**1.0512 × 10^2^**
F16	Ave	3.0000 × 10^3^	3.2477 × 10^3^	2.6889 × 10^3^	3.1296 × 10^3^	5.6500 × 10^3^	**2.6233 × 10^3^**	3.3348 × 10^3^	3.0179 × 10^3^	3.7045 × 10^3^	3.5009 × 10^3^
	Std	3.2304 × 10^2^	4.4899 × 10^2^	4.7518 × 10^2^	4.2166 × 10^2^	1.1409 × 10^3^	**2.9416 × 10^2^**	4.8144 × 10^2^	3.0997 × 10^2^	3.9168 × 10^2^	4.1156 × 10^2^
F17	Ave	2.1816 × 10^3^	2.5433 × 10^3^	2.1458 × 10^3^	2.4927 × 10^3^	5.4279 × 10^3^	2.3509 × 10^3^	2.6511 × 10^3^	2.4399 × 10^3^	2.6096 × 10^3^	**2.1371 × 10^3^**
	Std	**1.7367 × 10^2^**	2.9551 × 10^2^	1.9249 × 10^2^	2.3846 × 10^2^	2.9885 × 10^3^	2.2457 × 10^2^	3.4939 × 10^2^	2.1788 × 10^2^	3.3961 × 10^2^	1.9385 × 10^2^
F18	Ave	1.8405 × 10^6^	6.1752 × 10^6^	2.7908 × 10^6^	2.0968 × 10^6^	3.8577 × 10^7^	1.5349 × 10^5^	4.4813 × 10^6^	7.1306 × 10^5^	4.9419 × 10^6^	**2.1786 × 10^3^**
	Std	2.0554 × 10^6^	7.4439 × 10^6^	3.0051 × 10^6^	3.7843 × 10^6^	2.6533 × 10^7^	1.7963 × 10^5^	7.9891 × 10^6^	8.0255 × 10^5^	4.8172 × 10^6^	**1.7720 × 10^2^**
F19	Ave	4.5212 × 10^5^	1.2027 × 10^7^	1.3183 × 10^7^	1.5548 × 10^5^	5.2944 × 10^8^	1.0669 × 10^4^	2.8982 × 10^6^	1.1711 × 10^5^	1.5882 × 10^7^	**1.9600 × 10^3^**
	Std	4.7659 × 10^5^	3.6859 × 10^7^	6.2873 × 10^7^	1.2825 × 10^5^	4.8099 × 10^8^	1.0829 × 10^4^	4.7264 × 10^6^	3.5855 × 10^5^	3.3276 × 10^7^	**1.4814 × 10^1^**
F20	Ave	2.5270 × 10^3^	2.7197 × 10^3^	2.5056 × 10^3^	2.8320 × 10^3^	2.8342 × 10^3^	2.5678 × 10^3^	2.7668 × 10^3^	2.5258 × 10^3^	2.4741 × 10^3^	**2.4737 × 10^3^**
	Std	1.5579 × 10^2^	2.3815 × 10^2^	2.3754 × 10^2^	2.1376 × 10^2^	2.1775 × 10^2^	2.2287 × 10^2^	2.3138 × 10^2^	1.6333 × 10^2^	**1.1804 × 10^2^**	1.4899 × 10^2^
F21	Ave	2.5038 × 10^3^	2.5072 × 10^3^	2.4307 × 10^3^	2.5183 × 10^3^	2.6940 × 10^3^	**2.4229 × 10^3^**	2.5461 × 10^3^	2.4621 × 10^3^	2.5699 × 10^3^	2.4376 × 10^3^
	Std	3.3115 × 10^1^	5.1337 × 10^1^	**3.1969 × 10^1^**	5.6171 × 10^1^	4.9412 × 10^1^	3.8673 × 10^1^	4.4543 × 10^1^	3.9417 × 10^1^	3.5032 × 10^1^	6.9536 × 10^1^
F22	Ave	6.4875 × 10^3^	6.6453 × 10^3^	4.9408 × 10^3^	6.0226 × 10^3^	8.7026 × 10^3^	4.1674 × 10^3^	4.4082 × 10^3^	**2.6967 × 10^3^**	7.0149 × 10^3^	5.3617 × 10^3^
	Std	3.1073 × 10^3^	1.3780 × 10^3^	2.8513 × 10^3^	2.3196 × 10^3^	1.0337 × 10^3^	2.0891 × 10^3^	2.2253 × 10^3^	1.3645 × 10^3^	**6.6848 × 10^2^**	3.6659 × 10^3^
F23	Ave	2.9345 × 10^3^	2.8249 × 10^3^	2.7961 × 10^3^	2.9656 × 10^3^	3.5580 × 10^3^	2.8369 × 10^3^	2.9943 × 10^3^	2.9003 × 10^3^	3.2704 × 10^3^	**2.7697 × 10^3^**
	Std	7.4589 × 10^1^	**3.3917 × 10^1^**	5.7442 × 10^1^	7.4723 × 10^1^	1.6525 × 10^2^	5.4928 × 10^1^	6.5915 × 10^1^	4.9662 × 10^1^	1.0519 × 10^2^	5.6761 × 10^1^
F24	Ave	3.0941 × 10^3^	2.9895 × 10^3^	2.9949 × 10^3^	3.1539 × 10^3^	3.8488 × 10^3^	3.0005 × 10^3^	3.1519 × 10^3^	**2.9877 × 10^3^**	3.5971 × 10^3^	2.9916 × 10^3^
	Std	6.0289 × 10^1^	**3.5286 × 10^1^**	7.0275 × 10^1^	1.0753 × 10^2^	1.9358 × 10^2^	5.5290 × 10^1^	8.0812 × 10^1^	4.8679 × 10^1^	1.3042 × 10^2^	7.6591 × 10^1^
F25	Ave	2.9706 × 10^3^	3.3752 × 10^3^	3.0471 × 10^3^	2.9402 × 10^3^	5.6181 × 10^3^	2.9031 × 10^3^	3.0006 × 10^3^	2.9790 × 10^3^	3.8235 × 10^3^	**2.8873 × 10^3^**
	Std	4.2343 × 10^1^	6.6312 × 10^2^	4.8338 × 10^1^	2.7639 × 10^1^	7.6250 × 10^2^	1.9787 × 10^1^	9.2980 × 10^1^	2.5355 × 10^1^	2.3067 × 10^2^	**1.8150 × 10^0^**
F26	Ave	5.2323 × 10^3^	5.8249 × 10^3^	5.0892 × 10^3^	6.9113 × 10^3^	1.0733 × 10^4^	5.7961 × 10^3^	7.0636 × 10^3^	5.9314 × 10^3^	9.3306 × 10^3^	**4.7549 × 10^3^**
	Std	1.2319 × 10^3^	6.1249 × 10^2^	**3.7273 × 10^2^**	1.1271 × 10^3^	1.1568 × 10^3^	1.1861 × 10^3^	5.7630 × 10^2^	1.8917 × 10^3^	5.6847 × 10^2^	5.2061 × 10^2^
F27	Ave	3.2676 × 10^3^	3.2556 × 10^3^	3.2785 × 10^3^	3.2935 × 10^3^	4.5662 × 10^3^	3.2698 × 10^3^	3.3334 × 10^3^	3.3425 × 10^3^	4.1283 × 10^3^	**3.2475 × 10^3^**
	Std	5.6630 × 10^1^	2.7073 × 10^1^	3.5541 × 10^1^	4.5543 × 10^1^	3.9070 × 10^2^	3.7195 × 10^1^	6.5735 × 10^1^	4.5592 × 10^1^	2.0098 × 10^2^	**2.0468 × 10^1^**
F28	Ave	3.3626 × 10^3^	4.0225 × 10^3^	3.4907 × 10^3^	3.3224 × 10^3^	6.9458 × 10^3^	3.2478 × 10^3^	3.4634 × 10^3^	3.3315 × 10^3^	5.7932 × 10^3^	**3.2155 × 10^3^**
	Std	4.8687 × 10^1^	6.9254 × 10^2^	9.2672 × 10^1^	3.2031 × 10^1^	9.0742 × 10^2^	2.2875 × 10^1^	1.0077 × 10^2^	2.7689 × 10^1^	5.6019 × 10^2^	**1.7754 × 10^1^**
F29	Ave	4.1608 × 10^3^	4.1656 × 10^3^	4.0554 × 10^3^	4.3255 × 10^3^	7.6515 × 10^3^	4.1090 × 10^3^	4.6125 × 10^3^	4.5665 × 10^3^	5.8117 × 10^3^	**3.8516 × 10^3^**
	Std	**2.2804 × 10^2^**	3.3835 × 10^2^	2.3526 × 10^2^	3.5356 × 10^2^	2.3481 × 10^3^	2.5235 × 10^2^	4.2578 × 10^2^	3.5319 × 10^2^	6.1723 × 10^2^	3.2507 × 10^2^
F30	Ave	2.9084 × 10^6^	1.9991 × 10^6^	1.8927 × 10^7^	1.9800 × 10^6^	2.0420 × 10^9^	1.9797 × 10^4^	3.0382 × 10^6^	1.7222 × 10^6^	1.8279 × 10^8^	**1.5675 × 10^4^**
	Std	2.3064 × 10^6^	4.4094 × 10^6^	1.5727 × 10^7^	1.6121 × 10^6^	1.1487 × 10^9^	2.0365 × 10^4^	5.2474 × 10^6^	2.0245 × 10^6^	2.3671 × 10^8^	**6.0037 × 10^3^**

**Table 6:** Results for various algorithms on the CEC2022 and CEC2017.

Statistical Results	CEC2022 Dim = 10 (+/=/-)	CEC2022 Dim = 20 (+/=/-)	CEC2017 Dim = 30 (+/=/-)
PSO	(9/0/3)	(11/0/1)	(25/0/5)
MFO	(11/0/1)	(10/0/2)	(27/1/2)
GWO	(10/0/2)	(8/0/4)	(30/0/0)
AVOA	(11/0/1)	(11/0/1)	(25/0/5)
AOA	(12/0/0)	(12/0/0)	(22/0/8)
INFO	(9/0/3)	(11/0/1)	(20/0/10)
DBO	(12/0/0)	(9/0/3)	(27/0/3)
BPBO	(10/0/2)	(10/0/2)	(25/0/5)
SOA	(11/0/1)	(12/0/0)	(26/0/4)

**Table 7:** Friedman mean rank test result.

Suites	CEC2022				CEC2017	
Dimensions	10		20		30	
Algorithms	M.R	T.R	M.R	T.R	M.R	T.R
PSO	5.83	6	5.67	7	5.10	5
MFO	4.83	4	5.42	5	5.93	7
GWO	5.33	5	5.58	6	4.87	4
AVOA	6.33	7	5.33	4	5.37	6
AOA	9.92	10	10.00	10	9.90	10
INFO	2.58	2	2.58	2	2.60	2
DBO	6.50	8	6.08	8	6.70	8
BPBO	4.67	3	5.00	3	4.33	3
SOA	7.67	9	7.50	9	8.20	9
MFISOA	**1.33**	**1**	**1.83**	**1**	**2.00**	**1**

**Table 8:** MFISOA results of multi-level threshold segmentation with Otsu as the objective function.

Images	TH = 4	TH = 6	TH = 8	TH = 10
baboon	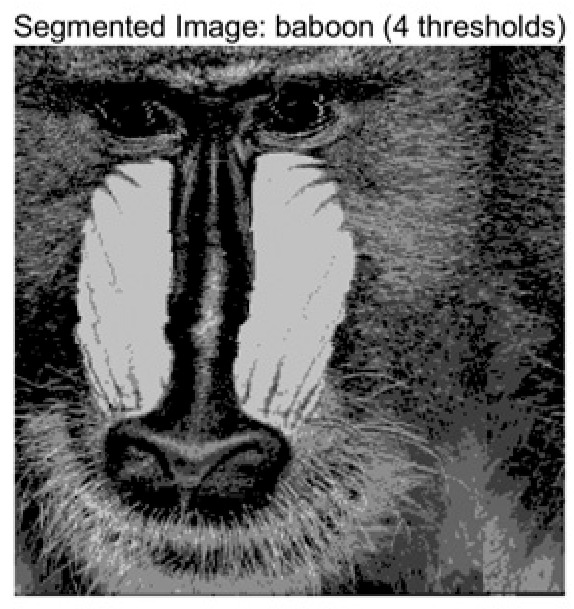	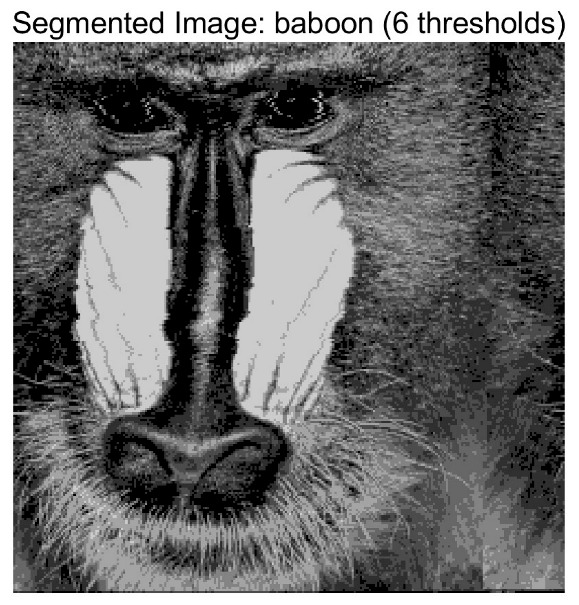	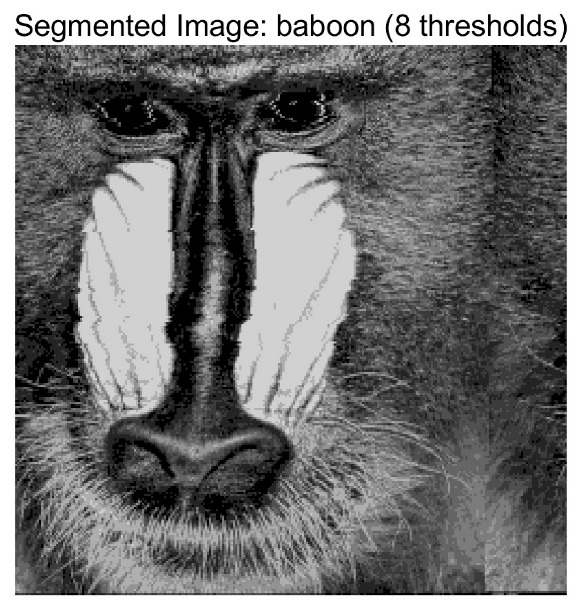	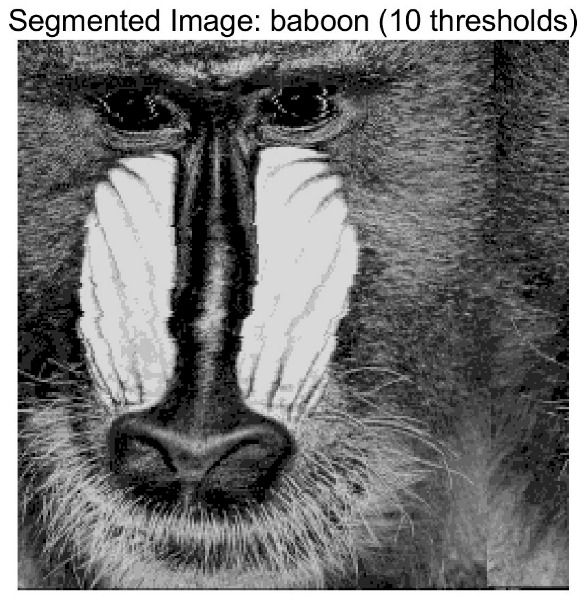
	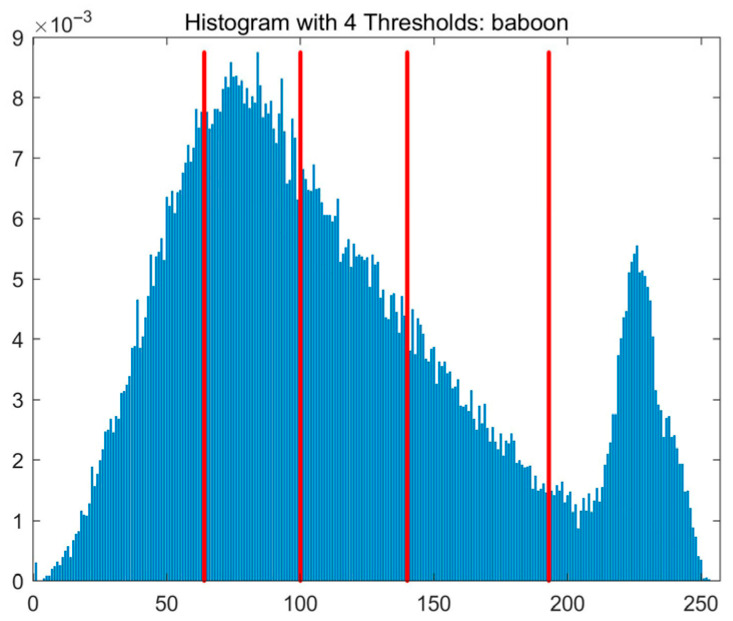	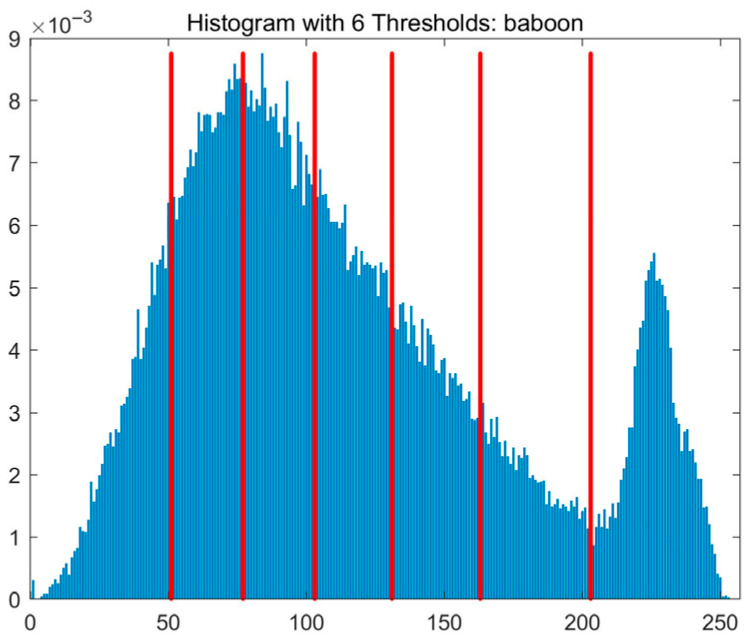	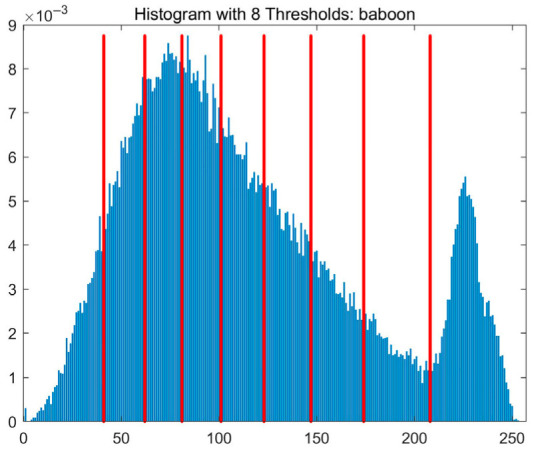	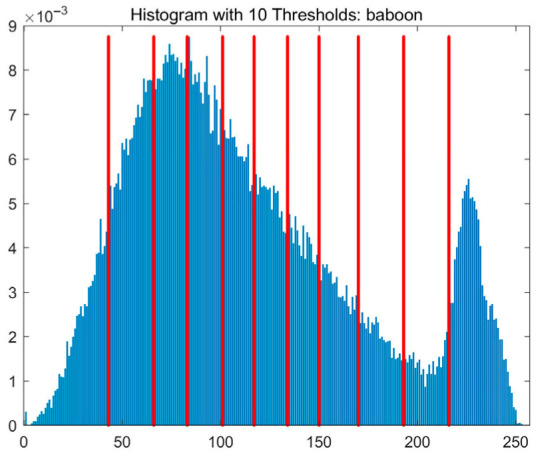
brain	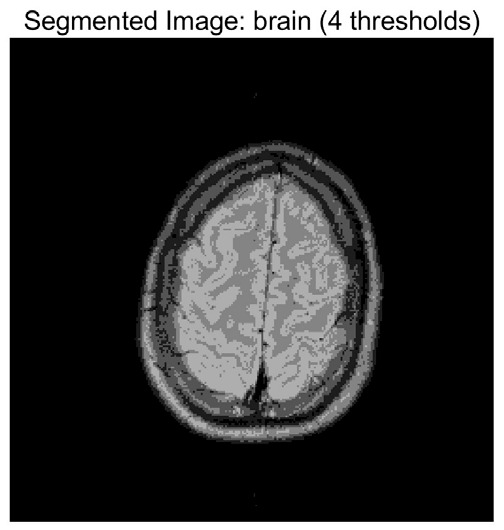	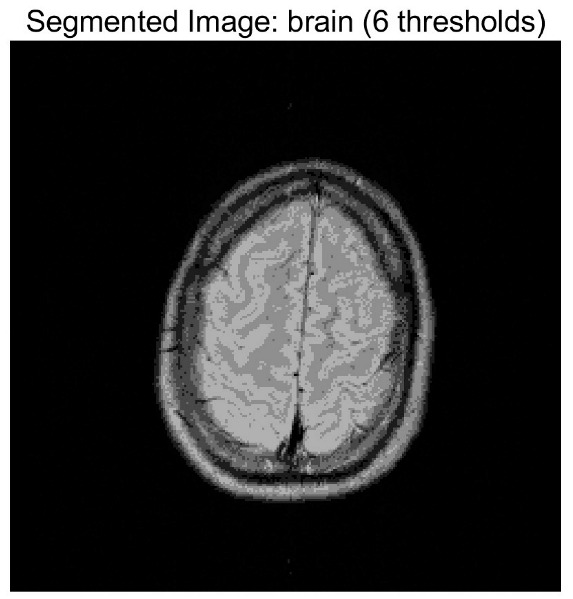	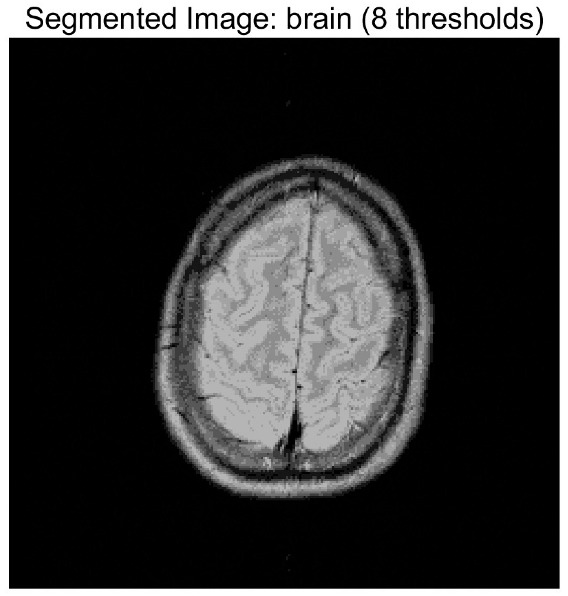	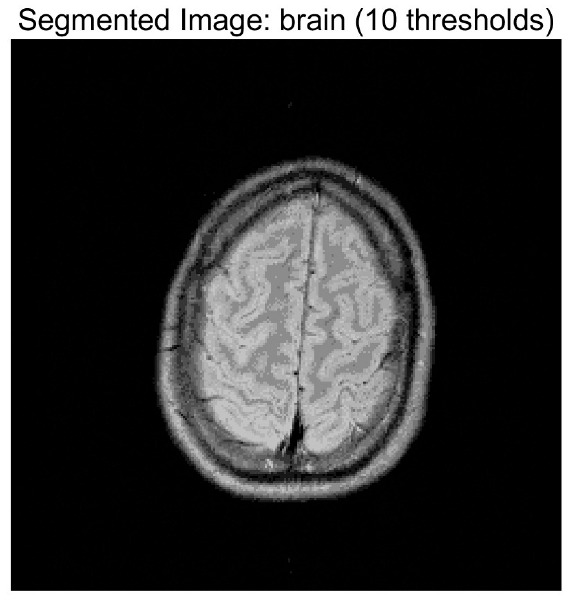
	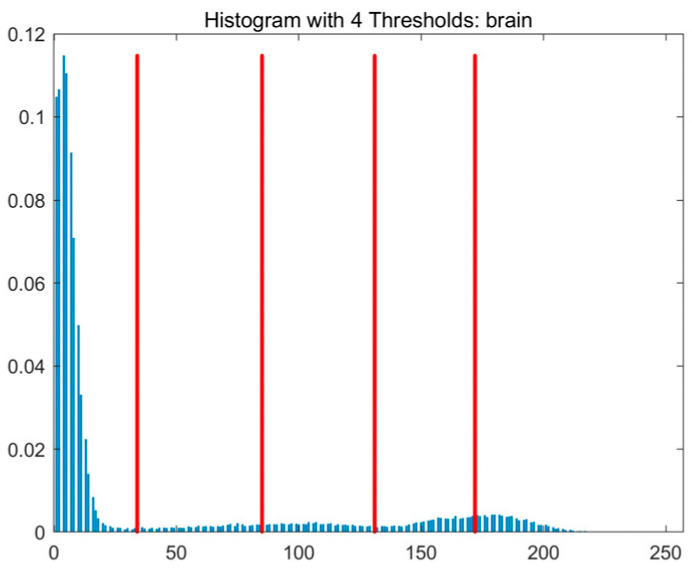	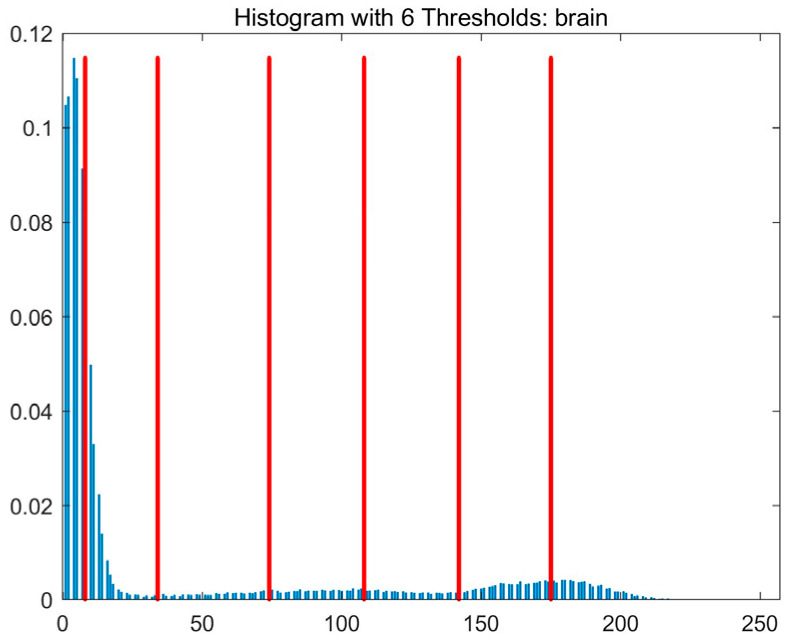	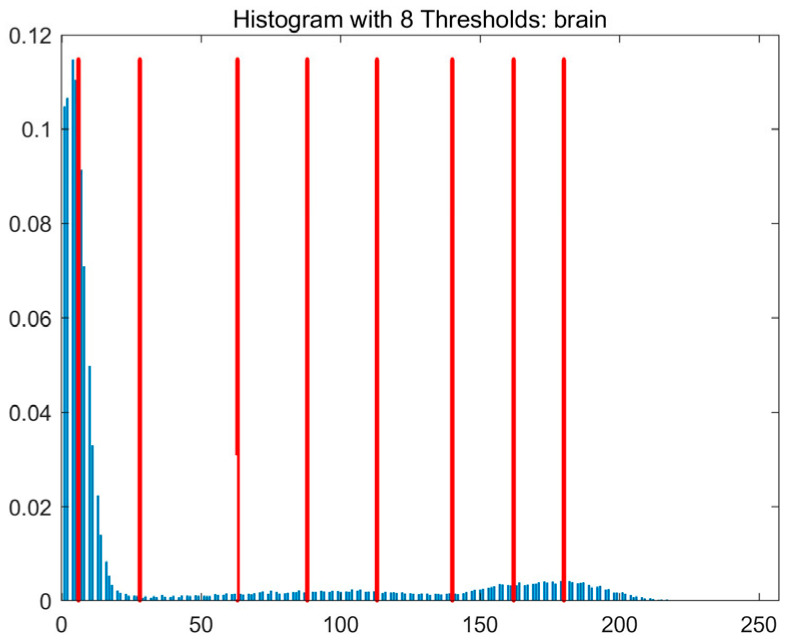	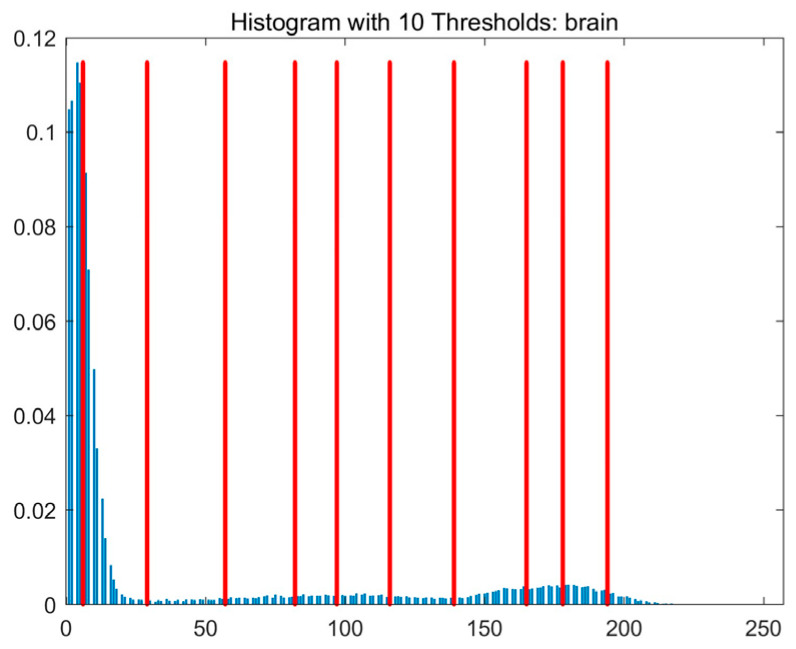
camera	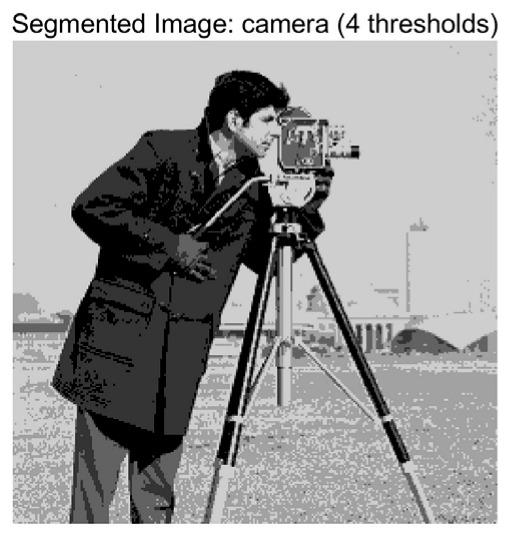	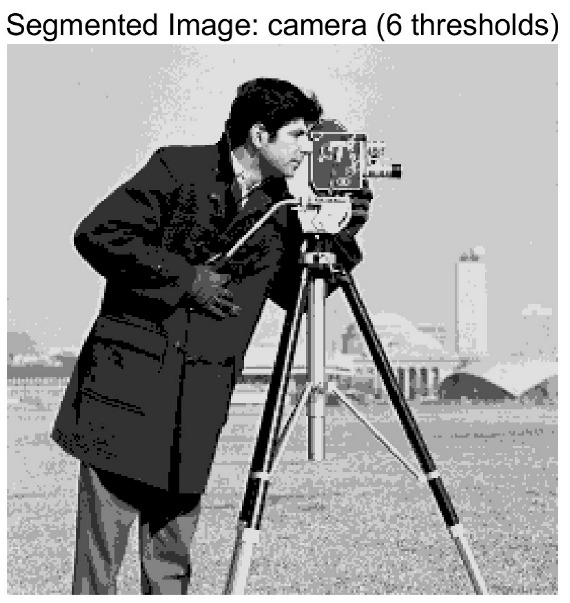	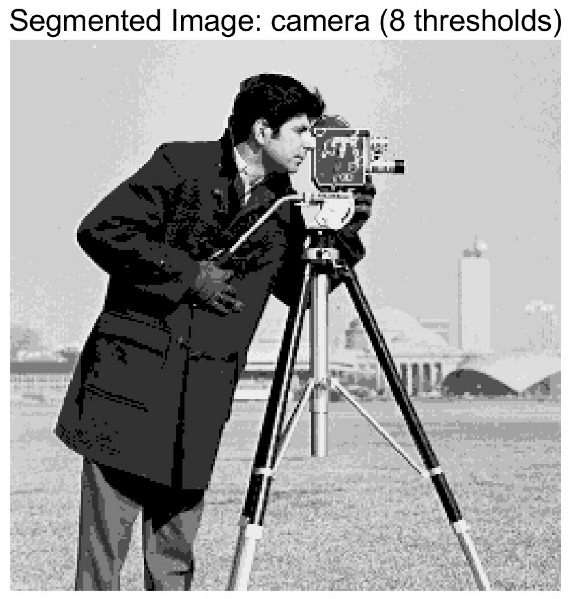	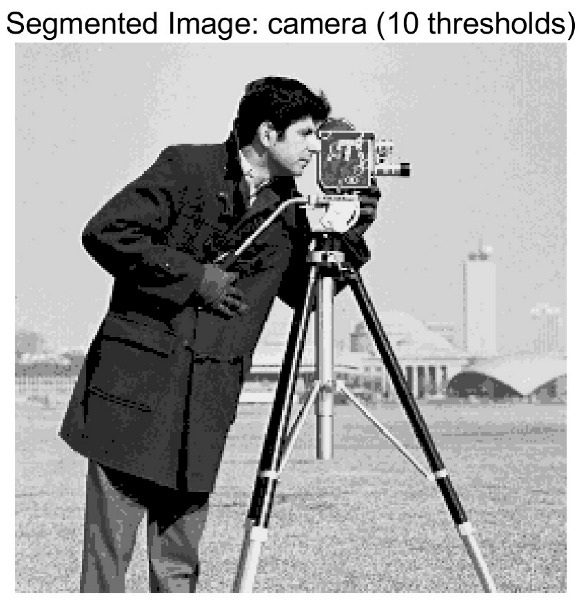
	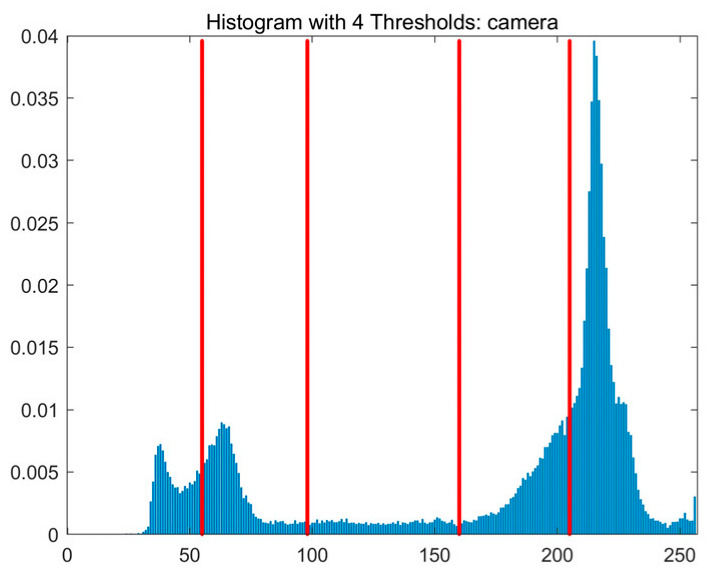	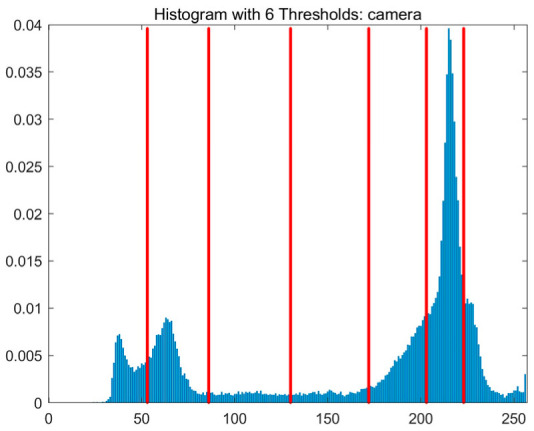	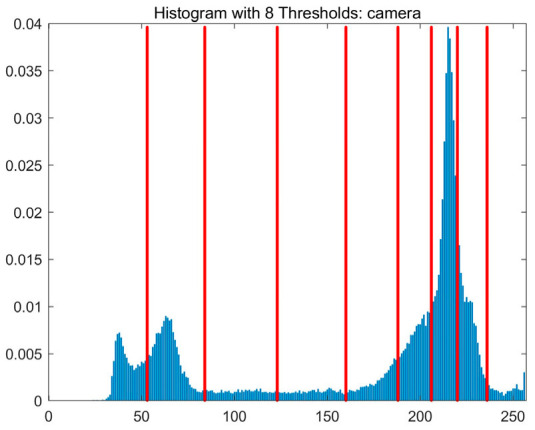	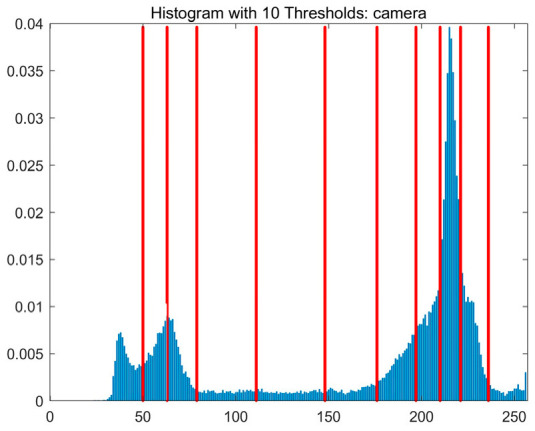
face	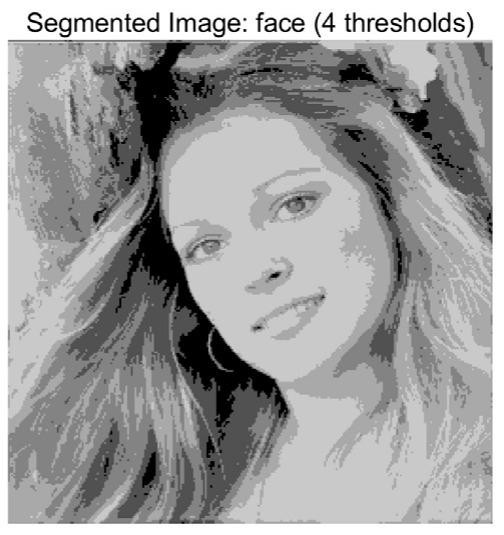	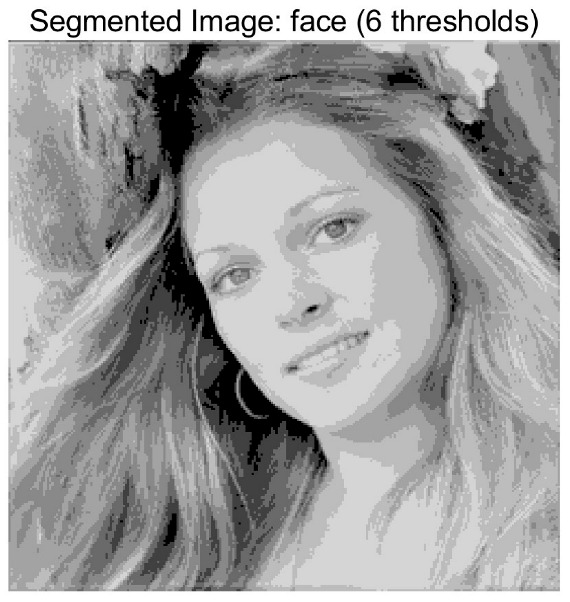	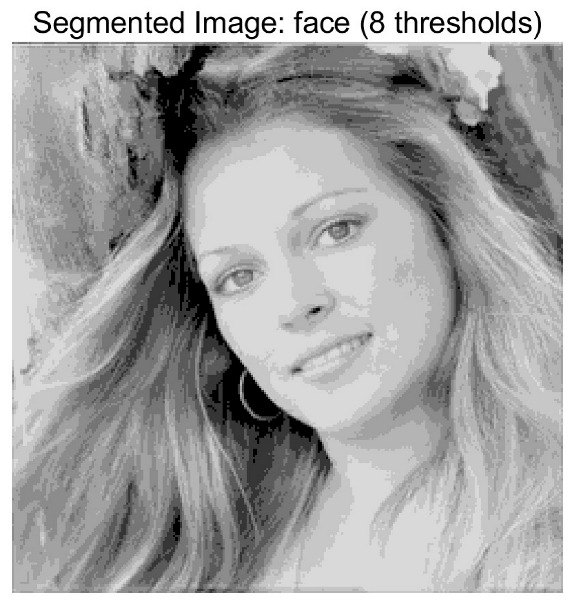	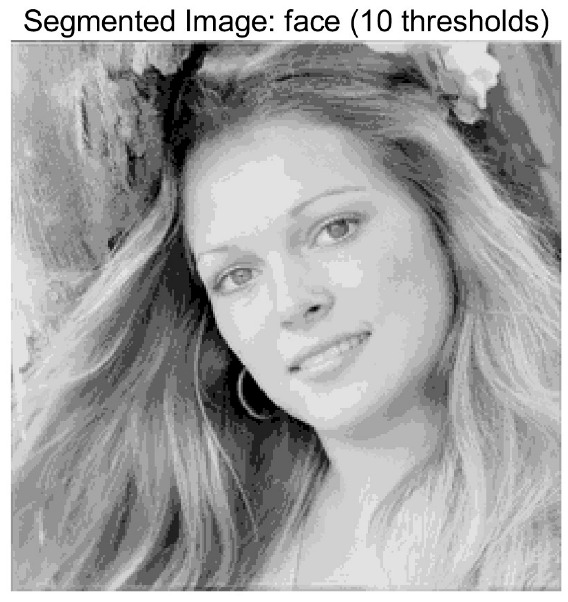
	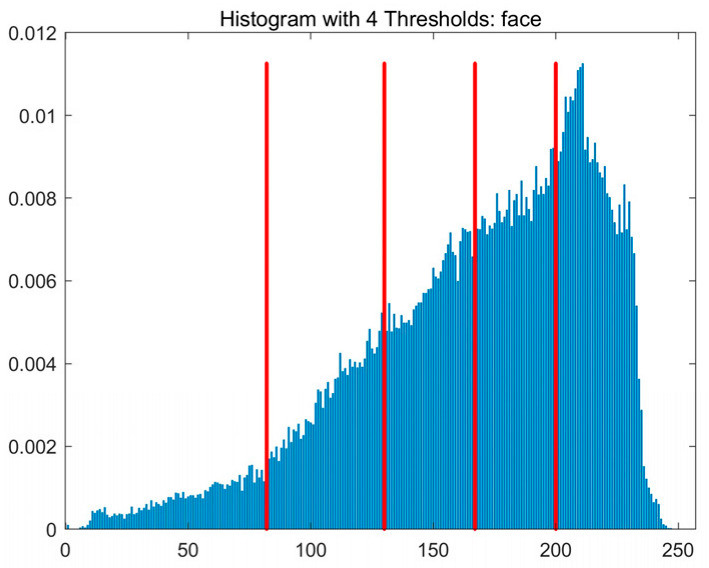	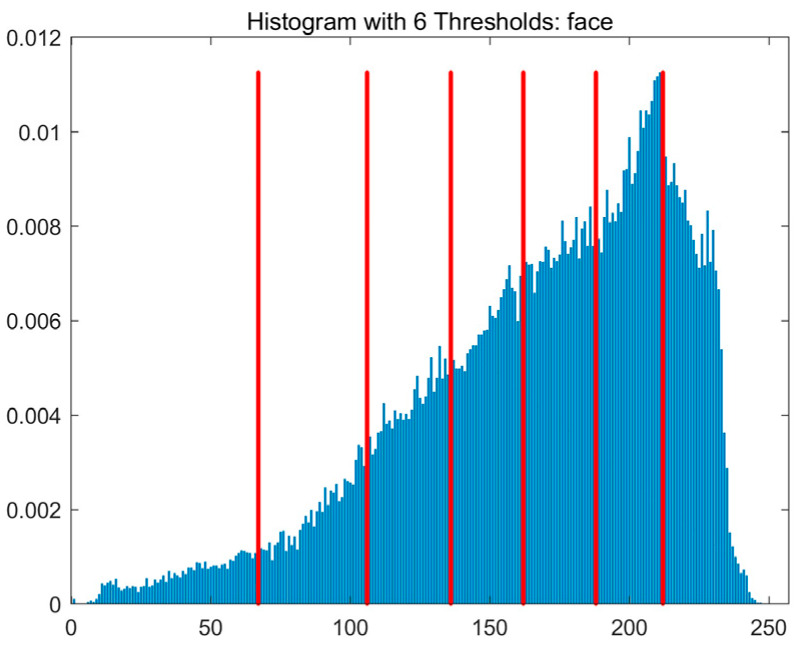	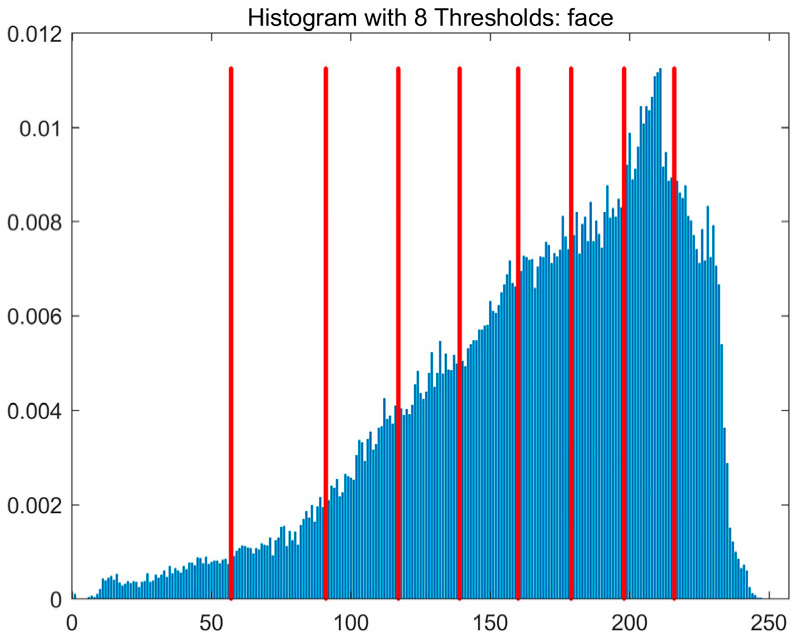	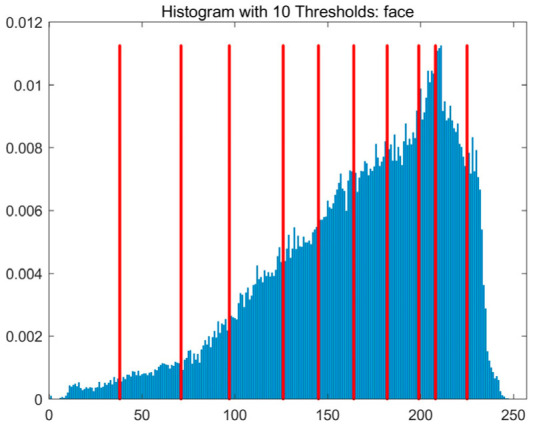
girl	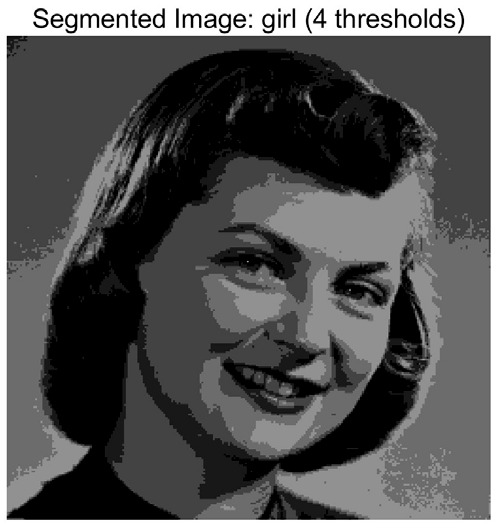	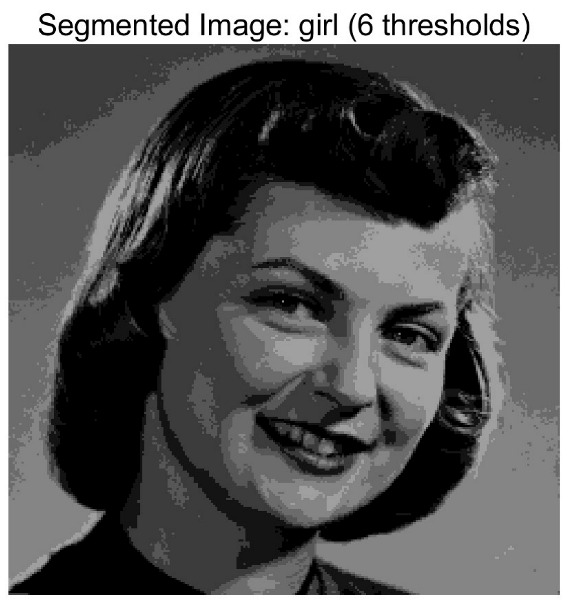	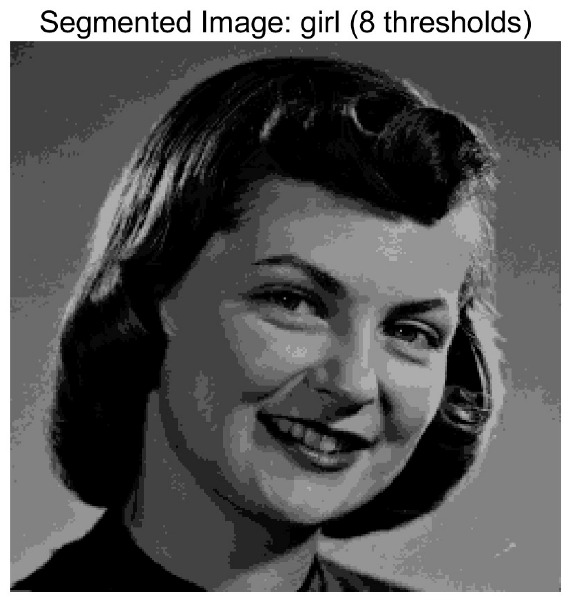	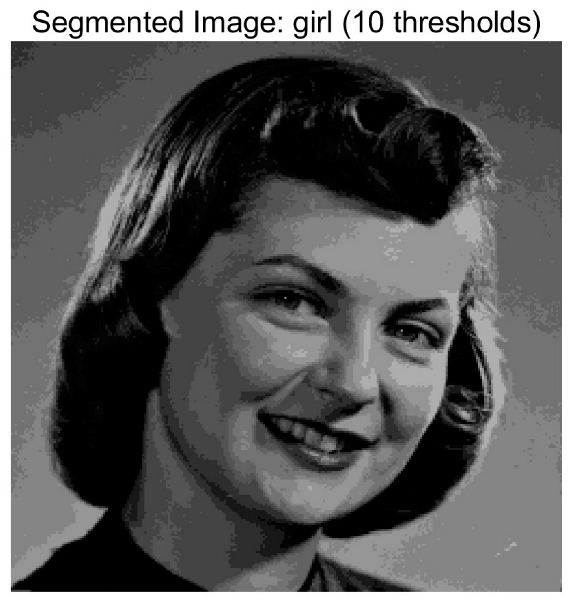
	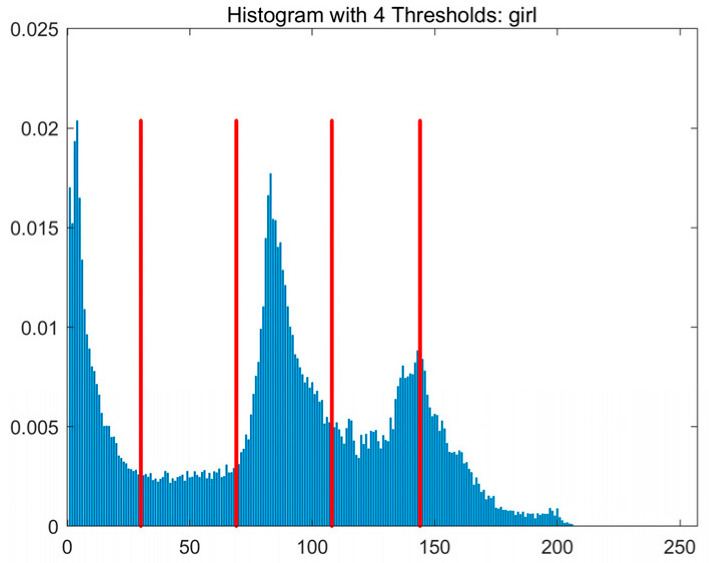	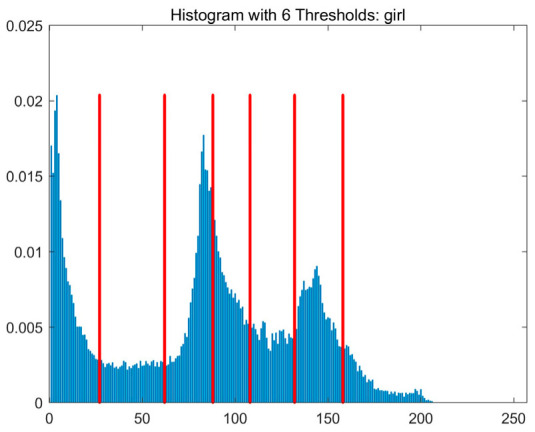	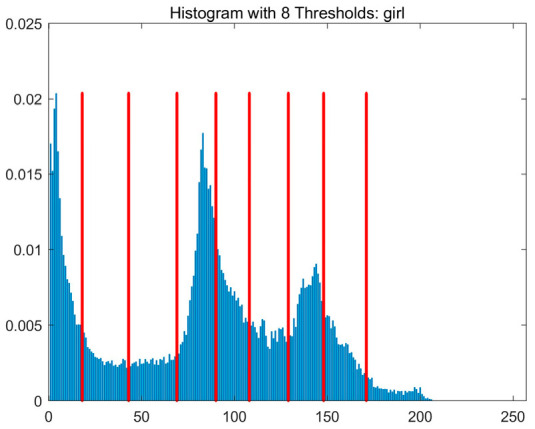	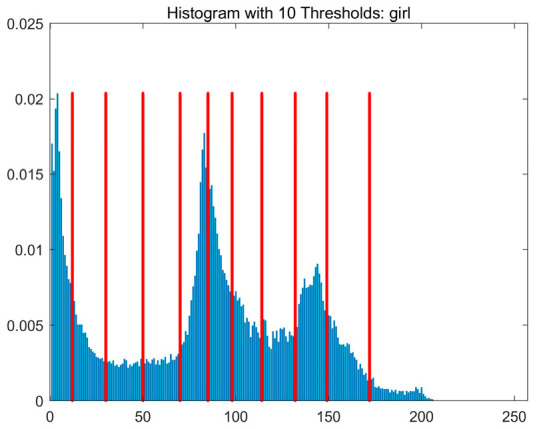
hunter	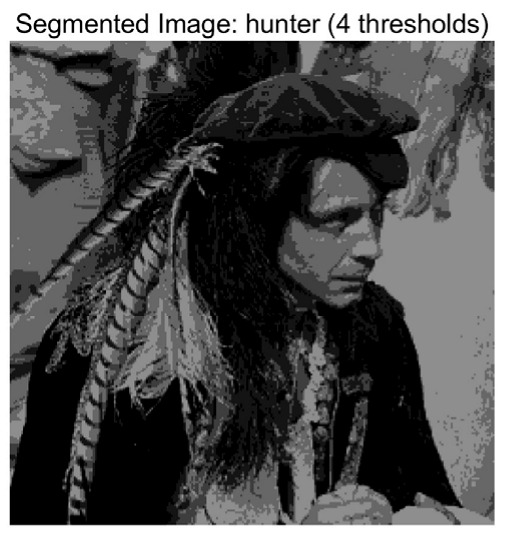	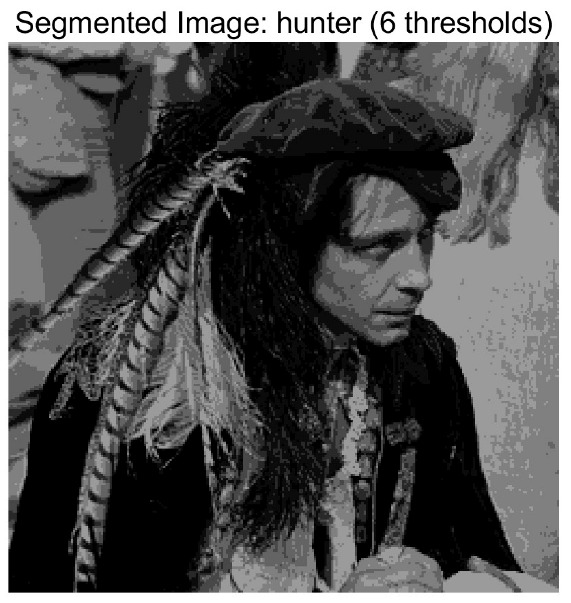	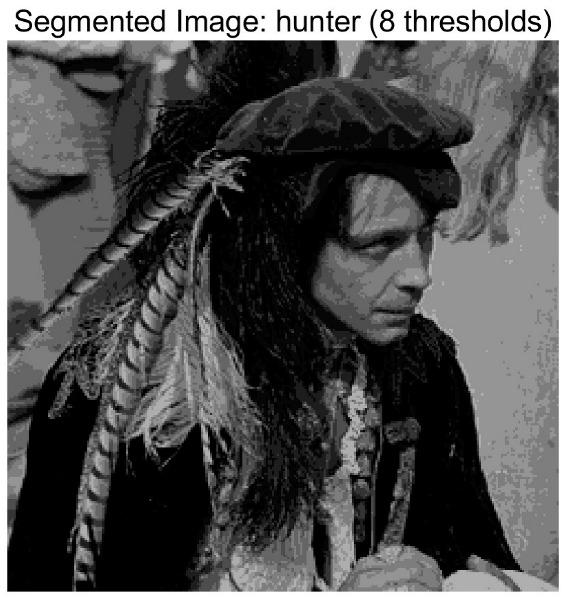	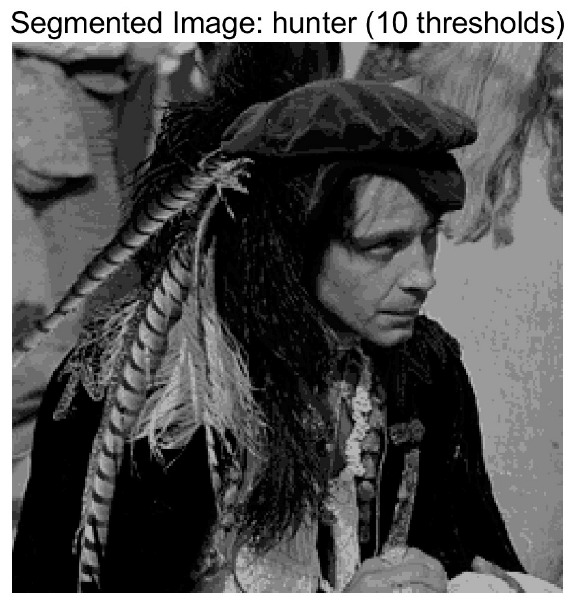
	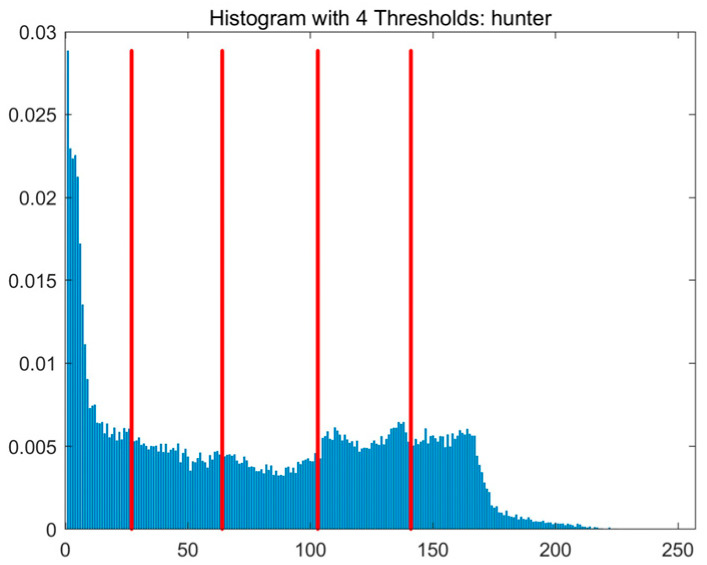	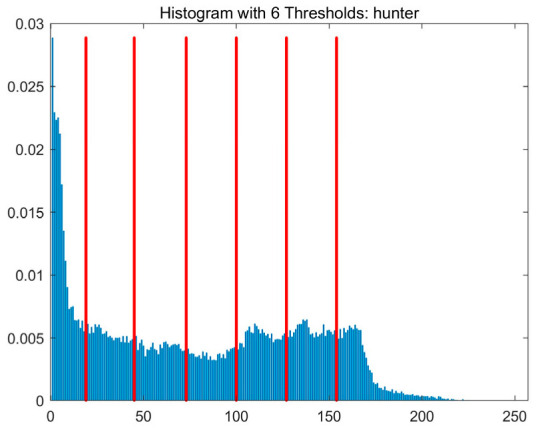	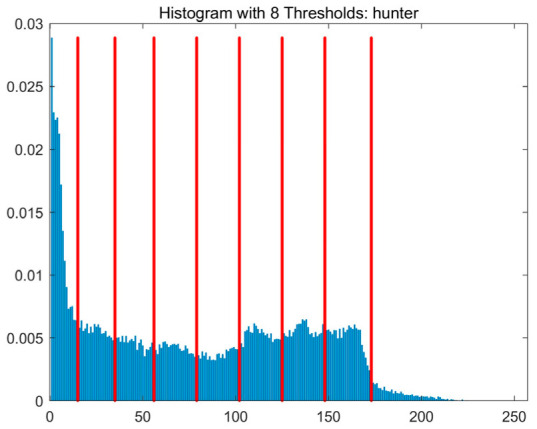	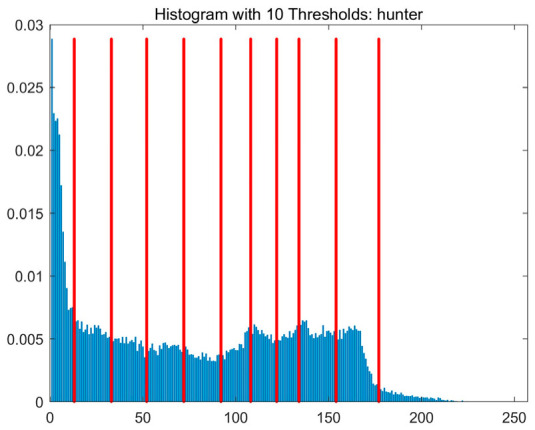
peppers	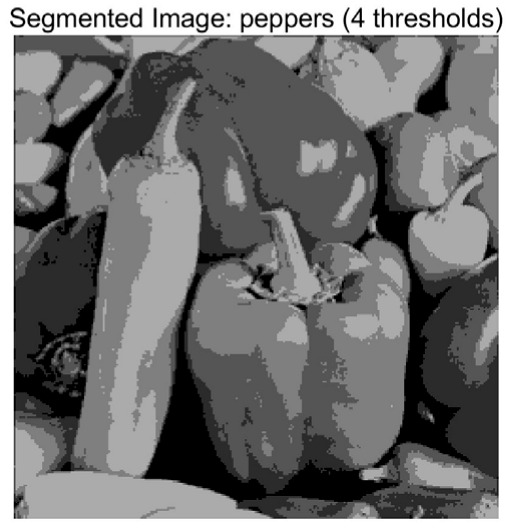	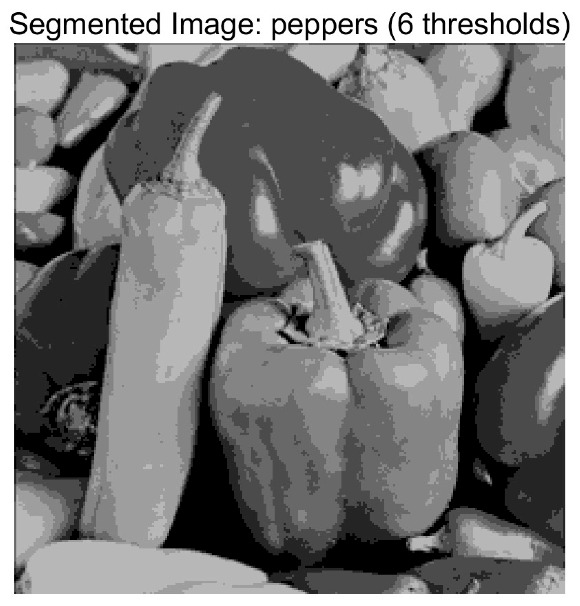	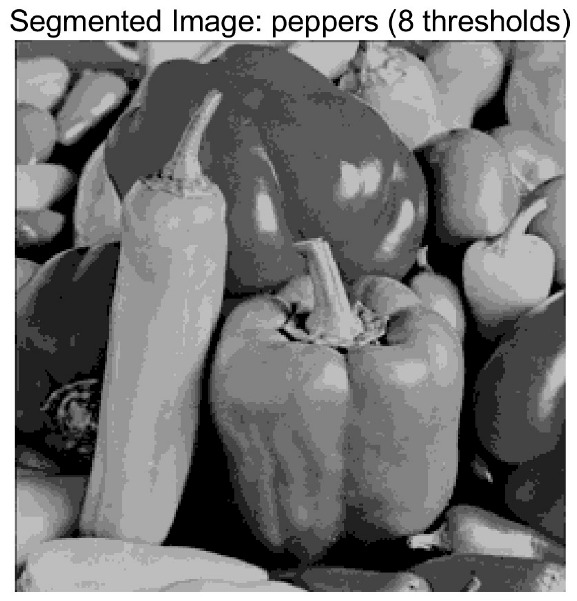	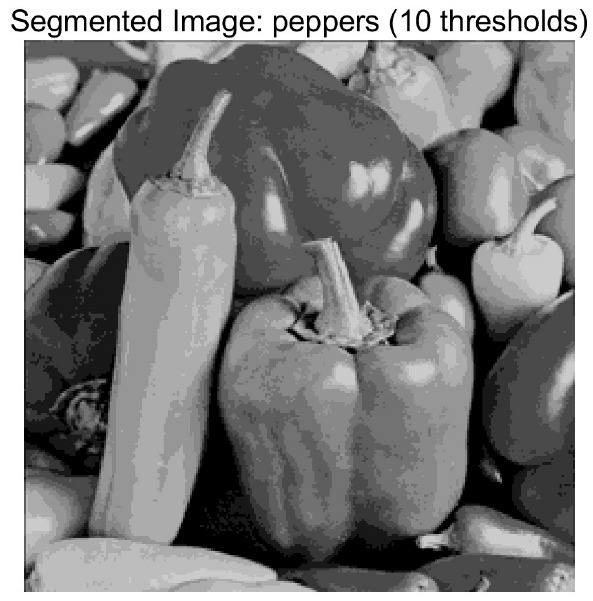
	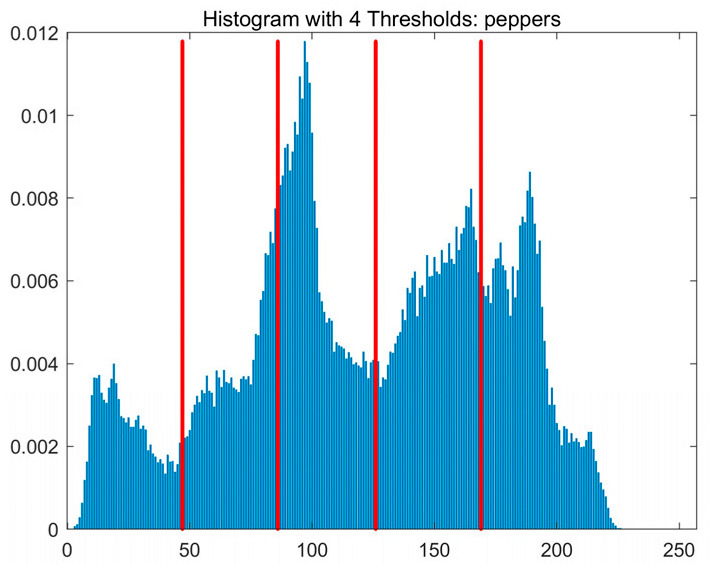	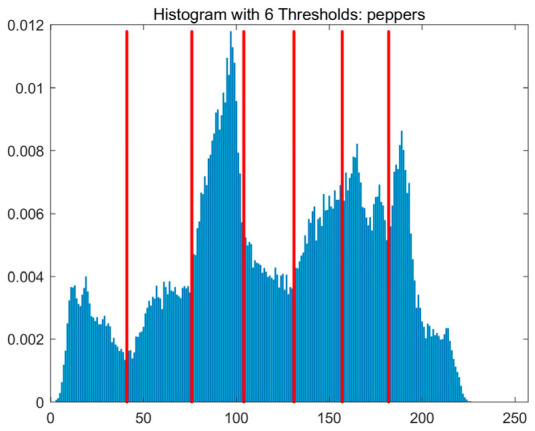	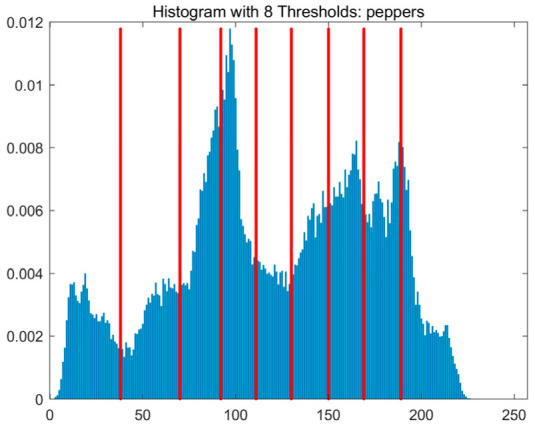	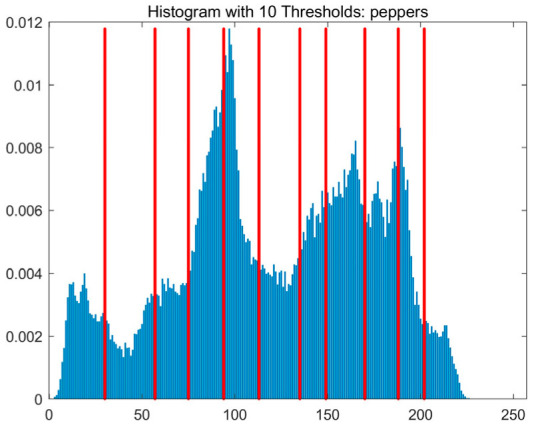
saturn	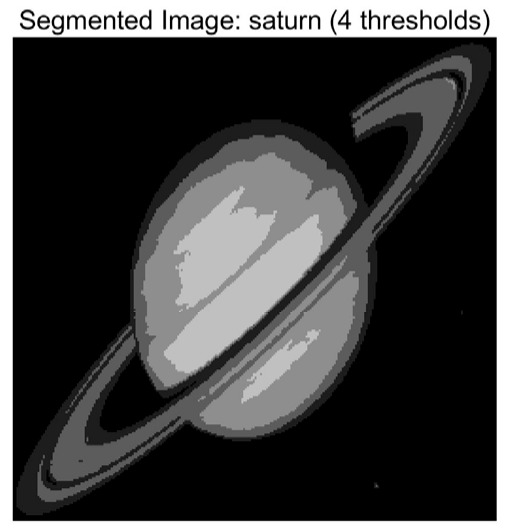	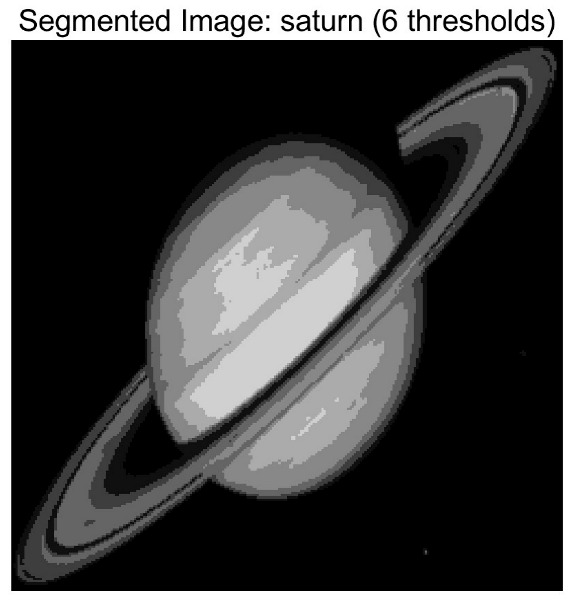	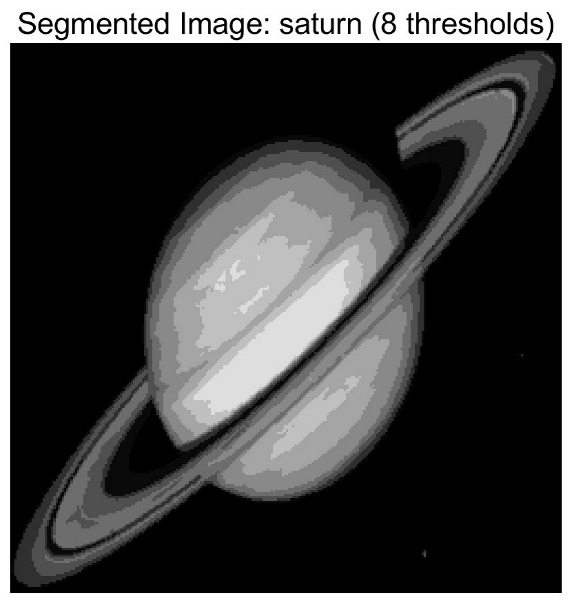	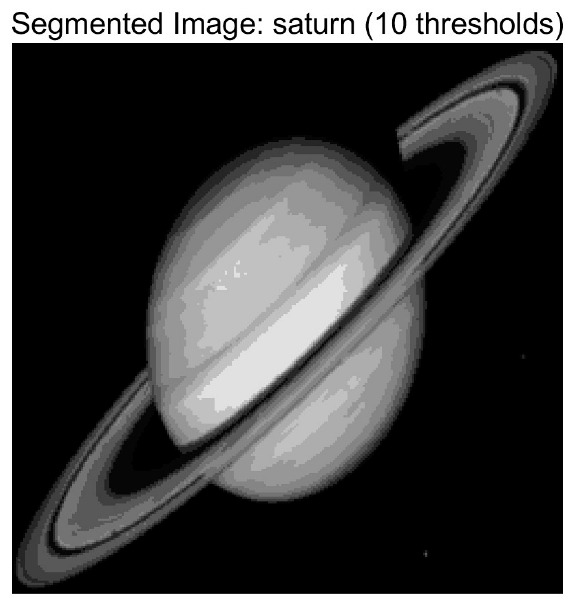
	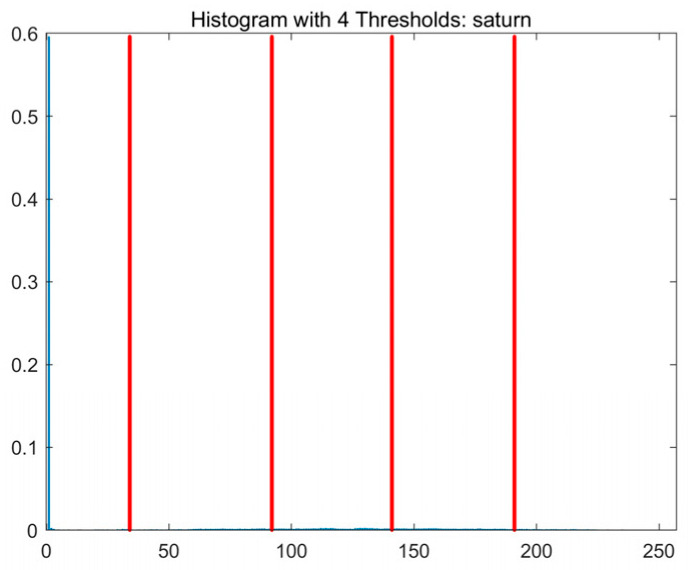	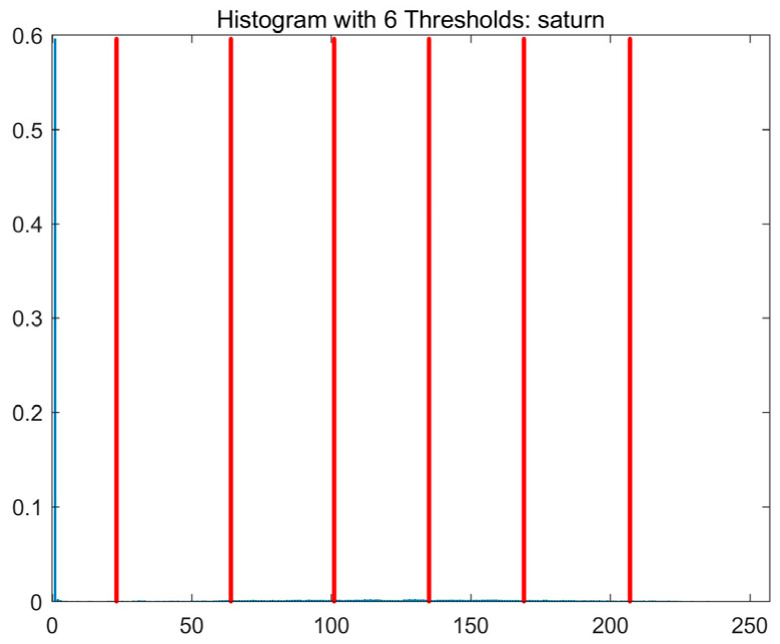	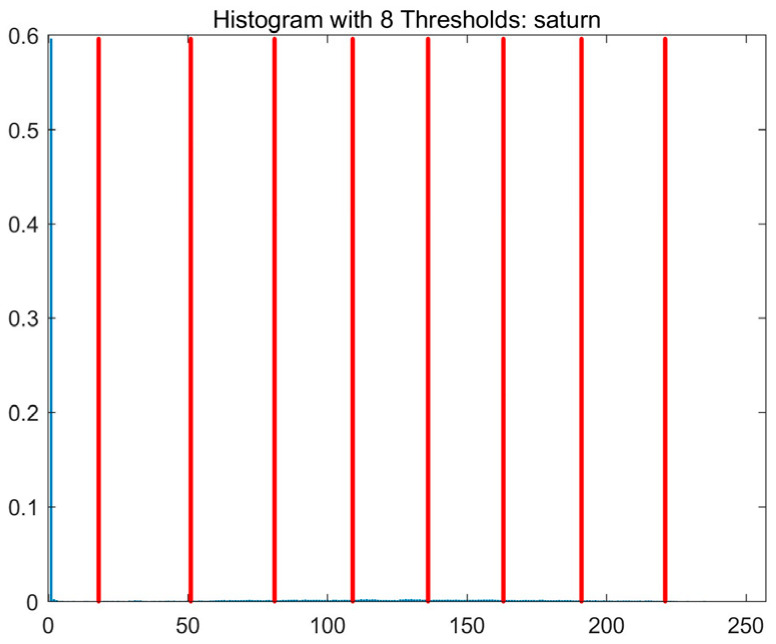	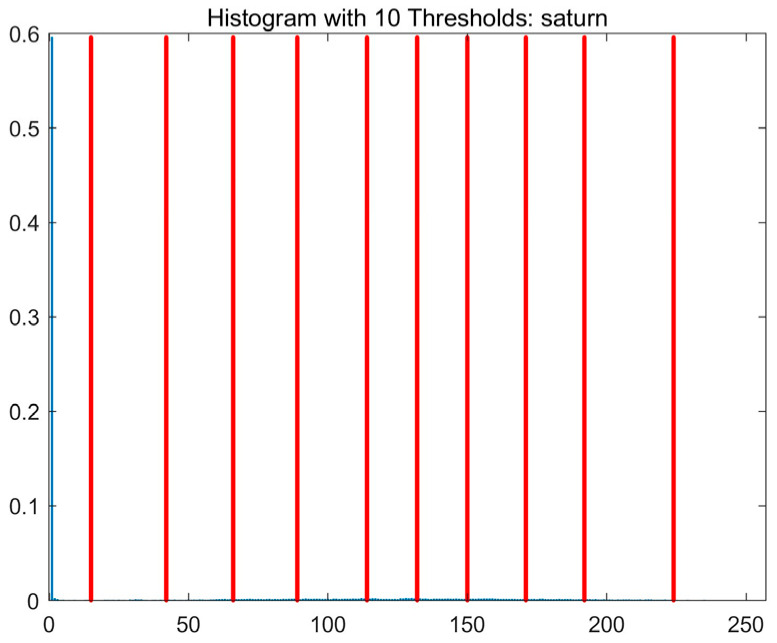
terrace	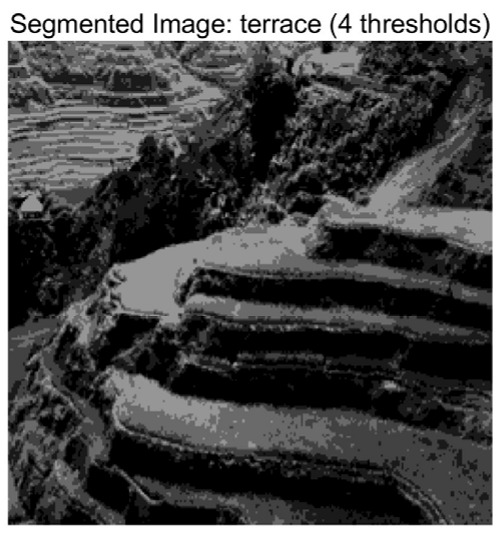	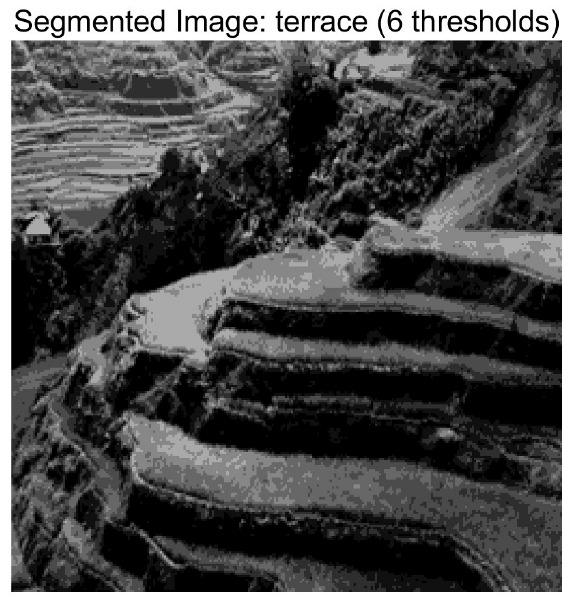	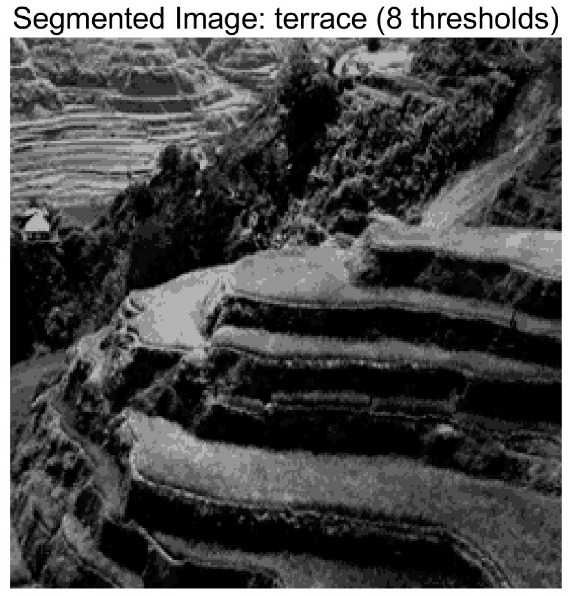	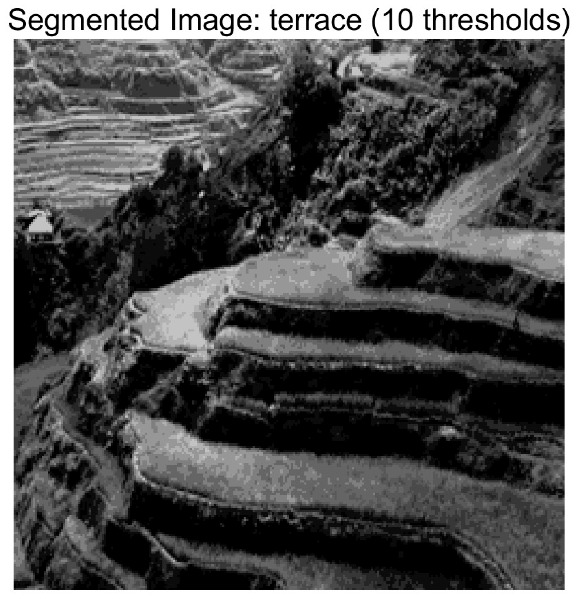
	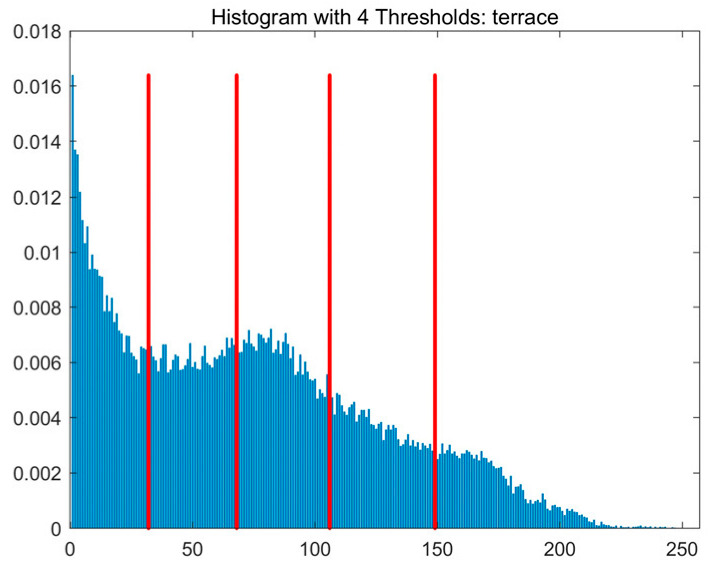	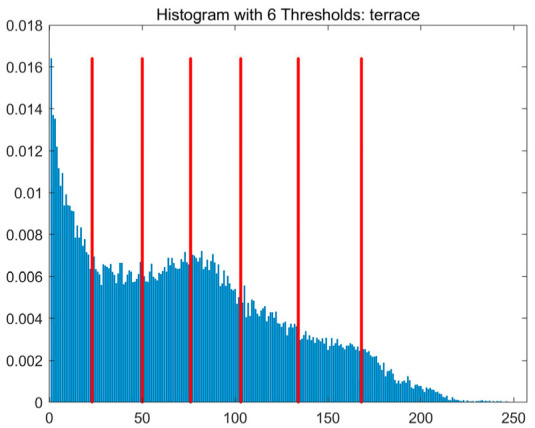	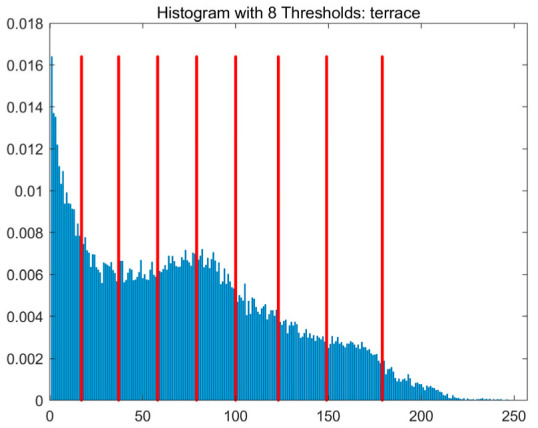	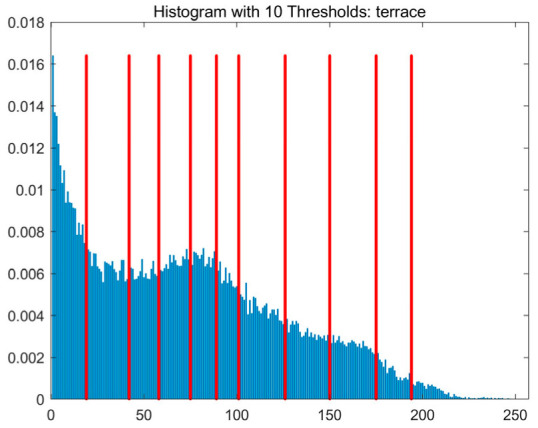

## Data Availability

All data in this paper are included in the manuscript.

## Supplementary Materials

The following supporting information can be downloaded at: https://www.mdpi.com/article/10.3390/biomimetics11040247/s1.
