# Supplementary material for: A Multi-Strategy Improved Seagull Optimization Algorithm for Global Optimization and Artistic Image Segmentation
Source: Biomimetics (Basel). 2026 Apr 3;11(4):247. doi: 10.3390/biomimetics11040247 (PMC13113111; doi:10.3390/biomimetics11040247)
Supplement: Supplementary file 1 [file biomimetics-11-00247-s001.zip › biomimetics-4181418-supplementary.pdf]

Table S1. Ave and Std of the optimal fitness values with Otsu as the objective function.

Images	TH	Metrics	PSO	MFO	GWO	AVOA	AOA	INFO	DBO	BPBO	SOA	MFISOA
baboon	4	Ave	3.3005E+03	3.3007E+03	3.2988E+03	3.2975E+03	3.2704E+03	3.2233E+03	3.3007E+03	3.2997E+03	3.3007E+03	**3.3008E+03**
		Std	2.3818E-01	2.5809E-01	2.9103E+00	1.5501E+01	1.6145E+01	3.8813E+01	1.1284E-01	1.3402E+00	9.5401E-02	**5.2435E-02**
	6	Ave	3.3700E+03	3.3707E+03	3.3646E+03	3.3696E+03	3.3320E+03	3.3140E+03	3.3697E+03	3.3688E+03	**3.3715E+03**	3.3702E+03
		Std	1.0305E+00	1.7992E+00	8.4415E+00	2.4413E+00	2.1902E+01	1.9337E+01	5.2451E+00	3.0703E+00	**7.2470E-01**	1.9330E+00
	8	Ave	3.3958E+03	3.3977E+03	3.3935E+03	3.3974E+03	3.3692E+03	3.3509E+03	3.3972E+03	3.3953E+03	3.3949E+03	**3.3990E+03**
		Std	**1.7484E+00**	2.2066E+00	4.7571E+00	3.0795E+00	1.3627E+01	1.8783E+01	3.0695E+00	2.8897E+00	3.1012E+00	**1.2823E+00**
	10	Ave	3.4097E+03	3.4122E+03	3.4080E+03	3.4116E+03	3.3804E+03	3.3791E+03	3.4102E+03	3.4104E+03	**3.4124E+03**	3.4085E+03
		Std	**1.5366E+00**	2.1696E+00	3.3286E+00	4.0414E+00	8.7315E+00	1.4175E+01	3.5691E+00	2.7949E+00	1.5598E+00	3.2805E+00
brain	4	Ave	3.7305E+03	3.7305E+03	3.7298E+03	3.7301E+03	3.7226E+03	3.7059E+03	3.7305E+03	3.7302E+03	3.7304E+03	**3.7305E+03**
		Std	8.1265E-02	6.8214E-02	1.1274E+00	5.9817E-01	5.7970E+00	1.1018E+01	9.8730E-02	8.7574E-01	4.9637E-01	**5.7191E-02**
	6	Ave	3.7500E+03	3.7504E+03	3.7491E+03	3.7498E+03	3.7388E+03	3.7340E+03	3.7507E+03	3.7492E+03	**3.7508E+03**	3.7503E+03
		Std	6.1609E-01	1.0769E+00	1.4365E+00	1.4541E+00	5.8795E+00	6.3981E+00	8.4842E-01	1.0483E+00	**5.1904E-01**	7.4561E-01
	8	Ave	3.7616E+03	**3.7619E+03**	3.7604E+03	3.7604E+03	3.7494E+03	3.7477E+03	3.7616E+03	3.7586E+03	3.7609E+03	**3.7620E+03**
		Std	**1.1631E+00**	2.0852E+00	2.5449E+00	2.6521E+00	5.1859E+00	3.3123E+00	1.9335E+00	2.1853E+00	1.6633E+00	**9.1518E-01**
	10	Ave	3.7669E+03	**3.7678E+03**	3.7668E+03	3.7670E+03	3.7569E+03	3.7543E+03	3.7666E+03	3.7647E+03	3.7672E+03	3.7665E+03
		Std	**7.0742E-01**	1.0404E+00	2.0705E+00	2.0166E+00	3.2827E+00	3.7910E+00	1.7758E+00	2.5356E+00	1.1126E+00	1.1944E+00
camera	4	Ave	4.5997E+03	4.6001E+03	4.5982E+03	4.5992E+03	4.5898E+03	4.5762E+03	4.5993E+03	4.5986E+03	**4.6006E+03**	4.6003E+03
		Std	1.0332E+00	1.2983E+00	2.0305E+00	1.3807E+00	7.5749E+00	1.3030E+01	1.2687E+00	1.1663E+00	**7.3275E-01**	9.4657E-01
	6	Ave	4.6498E+03	4.6500E+03	4.6451E+03	4.6488E+03	4.6154E+03	4.6125E+03	4.6491E+03	4.6447E+03	4.6497E+03	**4.6500E+03**
		Std	2.0277E+00	3.6408E+00	5.6514E+00	2.3107E+00	1.2322E+01	1.2849E+01	3.3795E+00	6.3966E+00	2.5169E+00	**9.6199E-01**
	8	Ave	4.6660E+03	**4.6678E+03**	4.6647E+03	4.6652E+03	4.6422E+03	4.6341E+03	4.6670E+03	4.6652E+03	4.6660E+03	4.6666E+03
		Std	2.5309E+00	1.5630E+00	3.3959E+00	6.1301E+00	1.1335E+01	1.2423E+01	2.5157E+00	2.8582E+00	2.7523E+00	**1.4728E+00**
	10	Ave	4.6750E+03	**4.6780E+03**	4.6739E+03	4.6752E+03	4.6542E+03	4.6515E+03	4.6750E+03	4.6743E+03	4.6750E+03	4.6753E+03
		Std	1.8861E+00	2.0678E+00	3.2740E+00	2.9587E+00	9.2936E+00	9.5565E+00	3.6342E+00	2.5231E+00	2.9319E+00	**1.7976E+00**
face	4	Ave	2.1221E+03	2.1221E+03	2.1196E+03	2.1197E+03	2.0983E+03	2.0555E+03	**2.1224E+03**	2.1213E+03	2.1220E+03	2.1223E+03
		Std	4.2441E-01	5.9709E-01	2.8781E+00	1.2284E+01	1.5645E+01	2.3465E+01	**2.8313E-01**	1.3015E+00	9.2608E-01	2.9876E-01
	6	Ave	2.1829E+03	2.1821E+03	2.1782E+03	2.1818E+03	2.1496E+03	2.1312E+03	2.1821E+03	2.1826E+03	2.1819E+03	**2.1833E+03**
		Std	1.4813E+00	2.5633E+00	5.9093E+00	5.0750E+00	1.4477E+01	1.5541E+01	3.8950E+00	2.6500E+00	4.1825E+00	**1.3974E+00**
	8	Ave	2.2068E+03	2.2049E+03	2.2011E+03	2.2075E+03	2.1762E+03	2.1646E+03	2.2060E+03	2.2070E+03	2.2076E+03	**2.2078E+03**
		Std	**1.4254E+00**	2.8497E+00	5.9629E+00	2.8919E+00	1.5453E+01	1.5813E+01	4.1110E+00	3.0586E+00	2.5070E+00	3.4315E+00
	10	Ave	2.2193E+03	**2.2210E+03**	2.2143E+03	2.2184E+03	2.1939E+03	2.1906E+03	2.2179E+03	2.2191E+03	2.2198E+03	2.2171E+03
		Std	**1.7279E+00**	2.1516E+00	3.6623E+00	4.6301E+00	9.0448E+00	1.2445E+01	3.7018E+00	2.8880E+00	2.2281E+00	3.3359E+00
girl	4	Ave	2.5337E+03	2.5339E+03	2.5333E+03	2.5332E+03	2.5161E+03	2.4730E+03	2.5338E+03	2.5326E+03	2.5339E+03	**2.5339E+03**
		Std	3.1664E-01	1.9115E-01	1.1332E+00	1.1775E+00	1.2333E+01	3.5167E+01	2.0178E-01	2.2233E+00	9.3919E-02	**3.2119E-02**
	6	Ave	2.5831E+03	**2.5840E+03**	2.5818E+03	2.5809E+03	2.5553E+03	2.5332E+03	2.5825E+03	2.5827E+03	2.5840E+03	2.5836E+03
		Std	1.1755E+00	6.1909E-01	2.7428E+00	4.2608E+00	1.2849E+01	2.2362E+01	4.1287E+00	1.8284E+00	1.0718E+00	**5.2401E-01**
	8	Ave	2.6032E+03	**2.6044E+03**	2.6018E+03	2.6031E+03	2.5702E+03	2.5585E+03	2.6038E+03	2.6026E+03	2.6035E+03	2.6040E+03

 https://doi.org/10.3390/biomimetics11040247

		Std	1.8261E+00	1.8025E+00	3.5376E+00	2.8755E+00	2.8933E+01	2.3588E+01	3.2541E+00	2.0614E+00	2.4344E+00	**1.5241E+00**
	10	Ave	2.6128E+03	2.6148E+03	2.6124E+03	2.6138E+03	2.5821E+03	2.4865E+03	2.6129E+03	2.6119E+03	2.6130E+03	**2.6150E+03**
		Std	**1.3174E+00**	2.0728E+00	3.2262E+00	1.9769E+00	1.5210E+01	4.7008E+02	3.4610E+00	3.2549E+00	2.1873E+00	1.8567E+00
hunter	4	Ave	3.1899E+03	3.1901E+03	3.1893E+03	3.1893E+03	3.1677E+03	3.1290E+03	3.1902E+03	3.1891E+03	3.1902E+03	**3.1902E+03**
		Std	3.4192E-01	3.9255E-01	1.4396E+00	2.1472E+00	1.6625E+01	3.3058E+01	2.1862E-01	1.7684E+00	2.5425E-01	**1.9324E-01**
	6	Ave	3.2449E+03	3.2463E+03	3.2440E+03	3.2447E+03	3.2162E+03	3.1922E+03	3.2457E+03	3.2447E+03	**3.2467E+03**	3.2457E+03
		Std	1.1025E+00	9.4602E-01	3.7034E+00	2.3820E+00	1.3372E+01	2.3327E+01	1.6657E+00	1.5728E+00	**4.8318E-01**	1.7487E+00
	8	Ave	3.2682E+03	3.2699E+03	3.2681E+03	3.2687E+03	3.2359E+03	3.2308E+03	3.2687E+03	3.2656E+03	3.2685E+03	**3.2704E+03**
		Std	1.6069E+00	2.3529E+00	4.4476E+00	2.5927E+00	1.1877E+01	1.3968E+01	3.4261E+00	3.8655E+00	**1.5239E+00**	2.5532E+00
	10	Ave	3.2793E+03	3.2814E+03	3.2807E+03	3.2801E+03	3.2556E+03	3.2431E+03	3.2801E+03	3.2793E+03	3.2783E+03	**3.2823E+03**
		Std	1.7301E+00	2.5393E+00	2.8379E+00	3.0230E+00	9.3417E+00	1.5308E+01	4.2606E+00	2.8111E+00	**1.5197E+00**	2.5626E+00
peppers	4	Ave	2.7008E+03	2.7003E+03	2.6990E+03	2.6998E+03	2.6738E+03	2.6320E+03	2.6996E+03	2.6989E+03	2.7010E+03	**2.7011E+03**
		Std	3.5518E-01	1.8168E+00	3.3905E+00	1.7421E+00	1.6666E+01	3.7542E+01	2.4015E+00	2.5148E+00	2.2930E-01	**1.1154E-01**
	6	Ave	2.7663E+03	2.7672E+03	2.7643E+03	2.7639E+03	2.7303E+03	2.7118E+03	2.7666E+03	2.7646E+03	2.7674E+03	**2.7675E+03**
		Std	1.6711E+00	1.6741E+00	4.2501E+00	5.2990E+00	1.3376E+01	2.2070E+01	2.2122E+00	2.8792E+00	**1.2877E+00**	1.8727E+00
	8	Ave	2.7913E+03	2.7918E+03	2.7881E+03	2.7910E+03	2.7550E+03	2.7466E+03	2.7902E+03	2.7901E+03	2.7901E+03	**2.7918E+03**
		Std	**2.1048E+00**	2.6571E+00	3.6512E+00	3.6234E+00	1.6894E+01	1.7735E+01	4.2232E+00	3.5763E+00	3.8415E+00	2.6160E+00
	10	Ave	2.8034E+03	2.8051E+03	2.8003E+03	2.8036E+03	2.7697E+03	2.7648E+03	2.8007E+03	2.8025E+03	2.8021E+03	**2.8052E+03**
		Std	**1.7421E+00**	2.1506E+00	2.7976E+00	2.7373E+00	1.7005E+01	2.0893E+01	3.4739E+00	2.7022E+00	1.8558E+00	2.3329E+00
saturn	4	Ave	5.2218E+03	5.2219E+03	5.2207E+03	5.2213E+03	5.2065E+03	5.1861E+03	5.2219E+03	5.2217E+03	5.2219E+03	**5.2220E+03**
		Std	1.6238E-01	6.7313E-02	2.9673E+00	1.5699E+00	9.8304E+00	2.0551E+01	2.0015E-01	5.2009E-01	2.1713E-01	**6.2896E-02**
	6	Ave	5.2720E+03	**5.2721E+03**	5.2694E+03	5.2714E+03	5.2549E+03	5.2492E+03	5.2708E+03	5.2702E+03	5.2720E+03	5.2715E+03
		Std	**7.6571E-01**	1.7333E+00	4.0683E+00	2.1571E+00	7.8017E+00	1.0118E+01	2.9416E+00	1.9980E+00	8.7286E-01	1.0528E+00
	8	Ave	5.2912E+03	5.2923E+03	5.2896E+03	5.2920E+03	5.2742E+03	5.2716E+03	5.2911E+03	5.2894E+03	5.2898E+03	**5.2925E+03**
		Std	1.3265E+00	1.6373E+00	3.1471E+00	2.2246E+00	6.4516E+00	7.0597E+00	2.2850E+00	2.5989E+00	1.9745E+00	**8.2271E-01**
	10	Ave	5.3007E+03	5.3005E+03	5.2994E+03	5.3015E+03	5.2866E+03	5.2862E+03	**5.3020E+03**	5.3002E+03	5.3014E+03	5.2983E+03
		Std	1.0959E+00	1.8813E+00	2.7384E+00	1.3799E+00	4.9299E+00	6.1979E+00	**1.0872E+00**	1.8086E+00	1.1579E+00	1.6014E+00
terrace	4	Ave	2.6399E+03	2.6401E+03	2.6392E+03	2.6389E+03	2.6150E+03	2.5728E+03	2.6401E+03	2.6393E+03	**2.6402E+03**	2.6402E+03
		Std	2.8435E-01	2.0268E-01	1.9830E+00	1.5997E+00	1.9814E+01	3.0628E+01	2.5010E-01	1.4943E+00	5.2466E-02	**4.7967E-02**
	6	Ave	2.6997E+03	2.7007E+03	2.6987E+03	2.6996E+03	2.6654E+03	2.6504E+03	2.7002E+03	2.6982E+03	**2.7016E+03**	2.7002E+03
		Std	1.6094E+00	2.2843E+00	3.1063E+00	4.5570E+00	1.7344E+01	2.1714E+01	3.6114E+00	2.9035E+00	**6.4397E-01**	2.2568E+00
	8	Ave	2.7248E+03	2.7272E+03	2.7236E+03	2.7259E+03	2.6910E+03	2.6864E+03	2.7248E+03	2.7249E+03	2.7241E+03	**2.7277E+03**
		Std	1.8350E+00	2.2434E+00	5.2620E+00	3.2933E+00	1.3842E+01	1.4058E+01	3.4848E+00	3.1590E+00	3.4025E+00	**1.1306E+00**
	10	Ave	2.7381E+03	2.7397E+03	2.7376E+03	2.7397E+03	2.7145E+03	2.7084E+03	2.7372E+03	2.7376E+03	**2.7411E+03**	2.7356E+03
		Std	2.0173E+00	2.5613E+00	3.9136E+00	2.1557E+00	9.9444E+00	1.2008E+01	3.3524E+00	2.9062E+00	2.1198E+00	**1.5398E+00**
Friedman-Rank			5.43	4.45	5.49	4.88	9.71	9.34	4.42	5.52	3.13	**2.70**
Final-Rank			6	4	7	5	10	9	3	8	2	**1**

Table S2. Ave and Std of all test images for SSIM in Otsu.

**Images**	**TH**	**Metrics**	PSO	MFO	GWO	AVOA	AOA	INFO	DBO	BPBO	SOA	MFISOA
baboon	4	Ave	**0.7254**	0.7243	0.7220	0.7216	0.6934	0.6510	0.7242	0.7152	0.7249	0.7240
		Std	4.81E-03	4.32E-03	1.09E-02	1.79E-02	3.68E-02	7.14E-02	3.76E-03	1.13E-02	2.96E-03	**2.46E-03**
	6	Ave	0.8365	0.8313	0.8223	**0.8409**	0.7708	0.7647	0.8295	0.8181	0.8320	0.8364

		Std	1.16E-02	1.18E-02	2.15E-02	1.31E-02	4.19E-02	4.79E-02	1.90E-02	1.41E-02	**1.04E-02**	1.16E-02
	8	Ave	0.8788	0.8811	0.8754	0.8861	0.8408	0.7972	0.8771	0.8603	**0.8863**	0.8837
		Std	1.61E-02	1.60E-02	1.82E-02	1.45E-02	3.55E-02	5.02E-02	1.87E-02	1.79E-02	1.43E-02	**1.25E-02**
	10	Ave	0.9009	0.9061	0.9026	0.9089	0.8567	0.8563	0.8979	0.8920	**0.9133**	0.9010
		Std	1.72E-02	1.39E-02	1.77E-02	**1.03E-02**	3.11E-02	4.29E-02	1.71E-02	1.28E-02	1.24E-02	1.72E-02
brain	4	Ave	0.3767	0.3768	0.3763	0.3765	0.3681	0.3499	**0.3769**	0.3768	0.3766	0.3768
		Std	6.54E-04	4.24E-04	1.42E-03	1.13E-03	6.46E-03	1.70E-02	5.15E-04	1.22E-03	7.38E-04	**4.03E-04**
	6	Ave	0.4768	0.5294	0.4838	0.5178	0.4378	0.4457	0.5422	0.4142	**0.5676**	0.5474
		Std	1.14E-01	1.44E-01	1.20E-01	1.41E-01	8.96E-02	1.02E-01	1.29E-01	**4.96E-02**	1.20E-01	1.47E-01
	8	Ave	0.6656	0.6813	0.6174	0.6174	0.4990	0.4703	**0.6888**	0.4908	0.6854	0.6812
		Std	**7.34E-02**	1.27E-01	1.14E-01	1.34E-01	1.25E-01	1.19E-01	1.26E-01	1.13E-01	7.85E-02	9.25E-02
	10	Ave	0.7196	0.7268	0.7333	0.7162	0.6196	0.5537	0.7390	0.6037	0.7320	**0.7650**
		Std	7.34E-02	6.88E-02	8.07E-02	9.89E-02	1.36E-01	1.44E-01	9.39E-02	1.25E-01	8.93E-02	**6.70E-02**
camera	4	Ave	0.7205	0.7283	0.7084	0.7077	0.6765	0.6742	0.7040	0.6914	**0.7441**	0.7365
		Std	3.52E-02	3.95E-02	4.19E-02	3.58E-02	4.01E-02	5.75E-02	3.68E-02	2.48E-02	**2.47E-02**	3.11E-02
	6	Ave	**0.8002**	0.7950	0.7837	0.7954	0.7563	0.7415	0.7908	0.7672	0.7932	0.7971
		Std	1.40E-02	2.18E-02	2.54E-02	2.31E-02	6.30E-02	6.11E-02	2.15E-02	3.34E-02	1.97E-02	**1.21E-02**
	8	Ave	0.8295	**0.8313**	0.8200	0.8228	0.7974	0.7841	0.8239	0.8165	0.8280	0.8241
		Std	2.03E-02	2.28E-02	2.22E-02	1.83E-02	4.39E-02	5.76E-02	2.05E-02	1.53E-02	1.88E-02	**9.03E-03**
	10	Ave	0.8458	0.8571	0.8409	0.8483	0.8127	0.8051	0.8452	0.8353	**0.8586**	0.8438
		Std	1.65E-02	1.38E-02	2.18E-02	2.19E-02	4.80E-02	6.01E-02	2.62E-02	**1.16E-02**	1.81E-02	2.40E-02
face	4	Ave	0.7068	0.7064	0.7035	0.7052	0.6810	0.6397	0.7071	0.7058	**0.7077**	0.7071
		Std	3.82E-03	4.63E-03	1.04E-02	1.02E-02	2.38E-02	3.00E-02	3.12E-03	4.65E-03	3.50E-03	**2.57E-03**
	6	Ave	0.7971	0.7971	0.7905	0.7956	0.7419	0.7294	0.7976	**0.7983**	0.7969	0.7964
		Std	4.64E-03	**4.40E-03**	1.24E-02	9.17E-03	2.53E-02	2.82E-02	4.47E-03	5.15E-03	5.71E-03	5.97E-03
	8	Ave	0.8473	0.8512	0.8370	0.8491	0.7868	0.7620	0.8476	0.8494	**0.8517**	0.8428
		Std	6.64E-03	7.93E-03	1.36E-02	7.30E-03	2.83E-02	2.40E-02	9.81E-03	9.81E-03	**5.87E-03**	1.08E-02
	10	Ave	0.8806	0.8734	0.8667	0.8785	0.8120	0.8086	0.8779	0.8810	0.8815	**0.8839**
		Std	7.52E-03	1.10E-02	1.21E-02	1.19E-02	2.50E-02	2.94E-02	9.80E-03	7.96E-03	6.98E-03	**6.83E-03**
girl	4	Ave	0.7144	0.7145	0.7133	0.7138	0.6963	0.6511	**0.7149**	0.7127	0.7144	0.7144
		Std	2.20E-03	1.13E-03	5.24E-03	2.61E-03	2.68E-02	3.90E-02	2.24E-03	6.18E-03	1.21E-03	**1.08E-03**
	6	Ave	0.7520	0.7577	0.7603	0.7652	0.7374	0.7132	0.7647	0.7523	**0.7659**	0.7562
		Std	**1.18E-02**	1.64E-02	2.01E-02	1.81E-02	2.83E-02	3.19E-02	1.72E-02	1.25E-02	1.58E-02	1.65E-02
	8	Ave	0.8003	0.8023	0.8017	0.7988	0.7607	0.7467	**0.8111**	0.7891	0.8009	0.7957
		Std	1.27E-02	**8.07E-03**	1.77E-02	1.44E-02	3.62E-02	4.21E-02	1.33E-02	1.39E-02	1.20E-02	9.10E-03
	10	Ave	0.8214	0.8342	0.8391	0.8314	0.7859	0.7730	**0.8405**	0.8128	0.8333	0.8294
		Std	1.52E-02	1.43E-02	1.80E-02	1.89E-02	4.25E-02	3.59E-02	1.61E-02	1.58E-02	1.41E-02	**1.30E-02**
hunter	4	Ave	0.7007	0.7012	0.7007	**0.7057**	0.6735	0.6414	0.7044	0.6934	0.7032	0.7049
		Std	8.27E-03	1.12E-02	1.12E-02	1.13E-02	3.14E-02	4.10E-02	7.49E-03	1.38E-02	7.35E-03	**7.28E-03**
	6	Ave	0.7715	0.7806	0.7722	0.7799	0.7303	0.7091	0.7765	0.7713	0.7751	**0.7815**
		Std	1.17E-02	1.06E-02	1.30E-02	8.73E-03	3.22E-02	3.74E-02	1.33E-02	1.13E-02	1.31E-02	**5.93E-03**
	8	Ave	0.8168	0.8219	0.8161	0.8203	0.7681	0.7549	**0.8257**	0.8018	0.8153	0.8185
		Std	1.10E-02	1.11E-02	1.08E-02	1.30E-02	2.80E-02	2.77E-02	1.37E-02	1.20E-02	1.07E-02	**1.01E-02**

	10	Ave	0.8411	0.8489	0.8479	0.8481	0.8005	0.7813	**0.8558**	0.8329	0.8380	0.8484
		Std	9.94E-03	1.70E-02	1.26E-02	1.24E-02	2.74E-02	3.72E-02	1.39E-02	1.14E-02	1.35E-02	**9.69E-03**
peppers	4	Ave	**0.7141**	0.7113	0.7090	0.7126	0.6902	0.6603	0.7088	0.7048	0.7136	0.7139
		Std	2.47E-03	7.15E-03	9.09E-03	7.21E-03	1.93E-02	3.21E-02	9.42E-03	8.56E-03	**1.53E-03**	2.63E-03
	6	Ave	0.7796	0.7813	0.7758	0.7796	0.7400	0.7314	0.7796	0.7781	0.7820	**0.7833**
		Std	7.19E-03	6.28E-03	9.04E-03	6.06E-03	2.01E-02	3.08E-02	5.68E-03	6.96E-03	7.31E-03	**4.28E-03**
	8	Ave	0.8126	0.8167	0.8129	**0.8181**	0.7764	0.7676	0.8177	0.8125	0.8132	0.8167
		Std	6.49E-03	5.25E-03	8.60E-03	6.73E-03	2.61E-02	2.77E-02	5.78E-03	6.87E-03	7.58E-03	**5.23E-03**
	10	Ave	0.8424	0.8469	0.8355	**0.8501**	0.8038	0.7974	0.8428	0.8395	0.8494	0.8426
		Std	**6.42E-03**	1.07E-02	7.92E-03	1.05E-02	2.53E-02	3.33E-02	1.09E-02	6.77E-03	6.68E-03	1.08E-02
saturn	4	Ave	0.8308	0.8316	0.8311	0.8307	0.8258	0.8219	0.8309	**0.8322**	0.8316	0.8312
		Std	2.09E-03	1.31E-03	3.69E-03	2.61E-03	1.43E-02	1.48E-02	**1.16E-03**	1.88E-03	1.32E-03	1.32E-03
	6	Ave	0.8787	0.8781	0.8765	0.8774	0.8580	0.8579	0.8772	0.8812	0.8794	**0.8812**
		Std	4.21E-03	5.24E-03	7.51E-03	5.85E-03	1.12E-02	1.42E-02	8.01E-03	3.03E-03	4.19E-03	**2.57E-03**
	8	Ave	0.9050	0.9063	0.9019	0.9048	0.8824	0.8776	0.9026	0.9052	0.9020	**0.9086**
		Std	5.07E-03	4.21E-03	7.02E-03	3.82E-03	1.06E-02	1.07E-02	6.72E-03	5.08E-03	5.75E-03	**2.37E-03**
	10	Ave	0.9223	0.9250	0.9209	0.9236	0.8990	0.8963	0.9210	0.9242	0.9163	**0.9255**
		Std	3.43E-03	3.33E-03	5.67E-03	3.83E-03	8.54E-03	1.23E-02	6.63E-03	3.89E-03	6.12E-03	**3.07E-03**
terrace	4	Ave	0.7185	0.7194	0.7181	0.7170	0.6899	0.6527	0.7191	0.7162	**0.7196**	0.7188
		Std	3.32E-03	2.71E-03	6.52E-03	5.83E-03	3.41E-02	4.11E-02	2.96E-03	4.85E-03	1.79E-03	**1.46E-03**
	6	Ave	0.8028	0.8028	0.7949	0.8037	0.7511	0.7340	**0.8072**	0.7885	0.8039	0.8046
		Std	9.24E-03	1.20E-02	9.65E-03	1.04E-02	3.57E-02	3.80E-02	1.20E-02	1.29E-02	1.10E-02	**6.71E-03**
	8	Ave	0.8439	**0.8578**	0.8514	0.8545	0.7912	0.7929	0.8534	0.8386	0.8514	0.8568
		Std	1.48E-02	1.21E-02	1.76E-02	1.34E-02	3.25E-02	3.52E-02	1.84E-02	1.31E-02	1.54E-02	**1.07E-02**
	10	Ave	0.8735	0.8847	0.8796	**0.8881**	0.8343	0.8269	0.8857	0.8632	0.8752	0.8859
		Std	1.54E-02	1.61E-02	1.72E-02	1.54E-02	2.66E-02	2.72E-02	1.43E-02	**1.37E-02**	1.42E-02	1.46E-02
Friedman-Rank			5.14	3.89	4.99	4.24	8.64	9.18	4.63	6.52	4.07	**3.70**
Final-Rank			7	2	6	4	9	10	5	8	3	**1**

Table S3. Ave and Std of all test images for FSIM in Otsu.

**Images**	**TH**	**Metrics**	PSO	MFO	GWO	AVOA	AOA	INFO	DBO	BPBO	SOA	MFISOA
baboon	4	Ave	**0.8207**	0.8197	0.8182	0.8181	0.8058	0.7827	0.8197	0.8156	0.8200	0.8194
		Std	2.63E-03	2.02E-03	6.51E-03	1.07E-02	1.99E-02	3.70E-02	2.02E-03	5.55E-03	1.62E-03	**1.24E-03**
	6	Ave	0.8877	0.8847	0.8783	**0.8918**	0.8449	0.8427	0.8835	0.8768	0.8838	0.8876
		Std	6.73E-03	**6.03E-03**	1.37E-02	1.02E-02	2.49E-02	2.68E-02	9.40E-03	8.70E-03	6.44E-03	7.35E-03
	8	Ave	0.9145	0.9178	0.9118	0.9188	0.8922	0.8653	0.9124	0.9000	**0.9189**	0.9171
		Std	1.22E-02	**9.44E-03**	1.30E-02	1.35E-02	2.41E-02	3.10E-02	1.17E-02	1.09E-02	1.12E-02	1.04E-02
	10	Ave	0.9291	0.9345	0.9328	0.9357	0.9021	0.9059	0.9286	0.9200	**0.9370**	0.9316
		Std	1.21E-02	1.07E-02	1.30E-02	9.48E-03	2.20E-02	2.81E-02	1.32E-02	**8.76E-03**	1.04E-02	1.33E-02
brain	4	Ave	0.6749	0.6750	0.6742	0.6746	0.6709	0.6594	0.6750	0.6746	0.6749	**0.6750**
		Std	3.66E-04	**2.93E-04**	1.45E-03	7.99E-04	1.01E-02	2.09E-02	4.43E-04	1.07E-03	7.77E-04	3.12E-04
	6	Ave	0.7607	0.7953	0.7772	0.7881	0.7499	0.7503	0.8209	0.7043	**0.8496**	0.8125
		Std	1.05E-01	1.13E-01	1.03E-01	1.10E-01	9.23E-02	9.50E-02	1.15E-01	**5.53E-02**	1.05E-01	1.14E-01

	8	Ave	0.9363	0.9081	0.9078	0.8825	0.7837	0.7668	0.9187	0.7856	0.9326	**0.9421**
		Std	4.44E-02	8.95E-02	7.38E-02	9.57E-02	9.84E-02	9.09E-02	7.36E-02	1.13E-01	4.17E-02	**1.40E-02**
	10	Ave	0.9546	**0.9588**	0.9550	0.9470	0.8647	0.8268	0.9494	0.9000	0.9557	0.9528
		Std	**4.63E-03**	4.66E-03	9.38E-03	4.80E-02	9.62E-02	1.03E-01	1.65E-02	9.41E-02	6.28E-03	6.28E-03
camera	4	Ave	0.8359	0.8329	0.8323	0.8329	0.8227	0.8118	0.8348	**0.8367**	0.8320	0.8337
		Std	5.02E-03	5.84E-03	6.91E-03	8.39E-03	1.51E-02	2.04E-02	7.28E-03	6.48E-03	**4.42E-03**	4.72E-03
	6	Ave	0.8754	0.8736	0.8673	0.8728	0.8452	0.8397	0.8727	0.8685	0.8731	**0.8760**
		Std	5.82E-03	5.55E-03	1.11E-02	9.77E-03	1.79E-02	1.93E-02	7.53E-03	7.71E-03	7.82E-03	**2.95E-03**
	8	Ave	0.8982	0.8976	0.8937	0.8937	0.8707	0.8620	0.8979	0.8916	0.8953	**0.9000**
		Std	5.85E-03	6.95E-03	9.68E-03	1.00E-02	1.48E-02	2.13E-02	7.44E-03	7.85E-03	7.18E-03	**3.39E-03**
	10	Ave	0.9095	0.9079	0.9069	0.9104	0.8826	0.8819	0.9102	0.9045	0.9112	**0.9142**
		Std	5.50E-03	8.79E-03	9.05E-03	8.93E-03	1.74E-02	1.92E-02	9.92E-03	6.14E-03	7.54E-03	**3.84E-03**
face	4	Ave	0.7538	0.7538	0.7515	0.7522	0.7317	0.6967	0.7541	0.7540	**0.7542**	0.7539
		Std	2.05E-03	1.99E-03	5.56E-03	1.09E-02	1.70E-02	2.05E-02	**1.18E-03**	2.21E-03	1.68E-03	1.46E-03
	6	Ave	0.8408	0.8411	0.8333	0.8390	0.7869	0.7697	0.8402	**0.8416**	0.8400	0.8398
		Std	4.19E-03	5.24E-03	1.06E-02	8.52E-03	1.99E-02	2.21E-02	5.15E-03	4.71E-03	6.63E-03	**3.08E-03**
	8	Ave	0.8843	0.8804	0.8729	0.8867	0.8250	0.8036	0.8847	0.8852	0.8876	**0.8882**
		Std	**5.16E-03**	8.99E-03	1.28E-02	7.14E-03	2.48E-02	2.27E-02	9.35E-03	8.91E-03	6.53E-03	7.03E-03
	10	Ave	0.9113	0.9055	0.8972	0.9105	0.8498	0.8449	0.9087	0.9109	0.9133	**0.9168**
		Std	6.48E-03	9.61E-03	1.07E-02	1.10E-02	2.14E-02	2.58E-02	9.68E-03	7.48E-03	5.99E-03	**5.79E-03**
girl	4	Ave	0.8288	0.8297	0.8287	0.8291	0.8133	0.7836	**0.8302**	0.8283	0.8299	0.8300
		Std	2.33E-03	1.58E-03	3.94E-03	1.51E-03	1.42E-02	2.11E-02	1.67E-03	3.90E-03	1.59E-03	**1.16E-03**
	6	Ave	0.8670	0.8690	0.8677	0.8670	0.8428	0.8237	0.8694	0.8661	**0.8718**	0.8687
		Std	**3.90E-03**	5.04E-03	5.64E-03	7.09E-03	1.41E-02	2.08E-02	7.63E-03	5.13E-03	5.11E-03	5.47E-03
	8	Ave	0.8995	0.9010	0.8982	0.8984	0.8595	0.8485	**0.9021**	0.8983	0.9019	0.8995
		Std	3.57E-03	**3.48E-03**	6.18E-03	5.13E-03	2.48E-02	2.58E-02	4.86E-03	5.39E-03	4.25E-03	4.44E-03
	10	Ave	0.9172	0.9229	0.9179	0.9199	0.8716	0.8624	0.9202	0.9153	**0.9236**	0.9193
		Std	**4.93E-03**	5.07E-03	8.58E-03	5.95E-03	2.42E-02	2.49E-02	7.94E-03	8.86E-03	5.02E-03	5.00E-03
hunter	4	Ave	0.8520	0.8518	0.8512	0.8517	0.8304	0.8029	0.8526	0.8500	**0.8527**	0.8524
		Std	1.46E-03	2.03E-03	1.93E-03	2.91E-03	1.35E-02	2.25E-02	**8.68E-04**	3.29E-03	9.71E-04	1.11E-03
	6	Ave	0.9038	0.9067	0.9030	0.9044	0.8715	0.8481	0.9056	0.9037	0.9056	**0.9073**
		Std	2.15E-03	1.89E-03	4.67E-03	4.03E-03	1.47E-02	2.22E-02	2.95E-03	2.41E-03	2.95E-03	**1.11E-03**
	8	Ave	0.9319	**0.9350**	0.9312	0.9319	0.8887	0.8826	0.9336	0.9274	0.9315	0.9347
		Std	3.23E-03	2.99E-03	6.50E-03	4.20E-03	1.43E-02	1.64E-02	5.10E-03	5.19E-03	4.04E-03	**2.78E-03**
	10	Ave	0.9449	0.9493	0.9473	0.9463	0.9105	0.8959	0.9487	0.9448	**0.9500**	0.9429
		Std	3.82E-03	4.86E-03	4.68E-03	4.87E-03	1.21E-02	2.00E-02	6.48E-03	4.73E-03	**2.88E-03**	5.68E-03
peppers	4	Ave	0.7863	0.7864	0.7848	0.7859	0.7735	0.7564	0.7864	0.7854	0.7863	**0.7866**
		Std	1.06E-03	9.13E-04	2.93E-03	2.19E-03	1.03E-02	1.58E-02	1.03E-03	1.49E-03	9.41E-04	**4.25E-04**
	6	Ave	0.8457	0.8471	0.8431	0.8439	0.8118	0.8020	0.8462	0.8436	0.8474	**0.8475**
		Std	3.17E-03	2.55E-03	6.84E-03	5.80E-03	1.26E-02	1.71E-02	2.75E-03	3.38E-03	**2.35E-03**	3.00E-03
	8	Ave	0.8800	0.8790	0.8764	0.8808	0.8378	0.8306	0.8802	0.8779	0.8822	**0.8824**
		Std	4.21E-03	6.12E-03	5.67E-03	5.62E-03	1.63E-02	1.57E-02	5.21E-03	5.86E-03	4.10E-03	**3.08E-03**
	10	Ave	0.9027	0.9004	0.8950	0.9033	0.8553	0.8505	0.8976	0.9009	0.9066	**0.9071**

		Std	**4.31E-03**	5.05E-03	5.83E-03	5.80E-03	1.89E-02	2.01E-02	6.71E-03	5.46E-03	4.50E-03	5.54E-03
saturn	4	Ave	0.8478	0.8483	0.8482	0.8476	0.8464	0.8450	0.8480	**0.8492**	0.8481	0.8481
		Std	1.46E-03	7.87E-04	3.33E-03	2.62E-03	1.12E-02	1.14E-02	**7.02E-04**	1.43E-03	7.70E-04	8.15E-04
	6	Ave	0.8842	0.8830	0.8826	0.8828	0.8697	0.8706	0.8841	**0.8883**	0.8860	0.8851
		Std	3.21E-03	3.35E-03	5.94E-03	3.00E-03	9.30E-03	1.30E-02	4.64E-03	3.04E-03	4.50E-03	**2.83E-03**
	8	Ave	0.9120	0.9117	0.9086	0.9099	0.8906	0.8855	0.9097	0.9116	0.9080	**0.9145**
		Std	4.35E-03	4.69E-03	5.96E-03	3.37E-03	9.91E-03	1.04E-02	5.47E-03	3.96E-03	5.66E-03	**2.90E-03**
	10	Ave	0.9278	0.9302	0.9256	0.9290	0.9042	0.9033	0.9261	0.9304	0.9221	**0.9306**
		Std	3.33E-03	**3.09E-03**	5.78E-03	3.47E-03	7.56E-03	1.11E-02	5.93E-03	3.51E-03	3.61E-03	4.65E-03
terrace	4	Ave	0.8447	0.8448	0.8438	0.8428	0.8235	0.7929	0.8446	0.8428	0.8446	**0.8449**
		Std	1.60E-03	9.61E-04	2.98E-03	3.93E-03	2.05E-02	2.50E-02	1.33E-03	2.71E-03	**7.35E-04**	7.60E-04
	6	Ave	0.9014	0.9032	0.8978	0.9019	0.8638	0.8488	**0.9038**	0.8957	0.9033	0.9028
		Std	4.95E-03	3.74E-03	5.60E-03	5.88E-03	1.80E-02	2.33E-02	4.16E-03	5.63E-03	**2.98E-03**	3.21E-03
	8	Ave	0.9290	**0.9354**	0.9288	0.9321	0.8864	0.8839	0.9307	0.9261	0.9341	0.9305
		Std	5.44E-03	4.50E-03	9.25E-03	6.64E-03	1.85E-02	1.72E-02	6.72E-03	5.91E-03	6.57E-03	**3.98E-03**
	10	Ave	0.9448	0.9498	0.9443	0.9488	0.9126	0.9059	0.9474	0.9406	0.9424	**0.9503**
		Std	6.27E-03	6.48E-03	8.17E-03	6.59E-03	1.42E-02	1.33E-02	6.19E-03	**5.91E-03**	6.34E-03	**5.05E-03**
Friedman-Rank			4.81	3.71	5.28	4.37	8.98	9.68	4.67	6.33	3.87	**3.33**
Final-Rank			6	2	7	4	9	10	5	8	3	**1**

Table S4. Ave and Std of all test images for PSNR in Otsu.

**Images**	**TH**	**Metrics**	PSO	MFO	GWO	AVOA	AOA	INFO	DBO	BPBO	SOA	MFISOA
baboon	4	Ave	**18.2274**	18.2083	18.1845	18.1458	1.67E+01	17.6539	18.2066	18.0075	18.2150	18.2022
		Std	1.04E-01	8.64E-02	2.37E-01	4.02E-01	1.18E+00	7.05E-01	7.87E-02	2.38E-01	6.39E-02	**5.20E-02**
	6	Ave	21.3898	21.2854	20.9955	**21.5233**	1.93E+01	19.6508	21.2524	20.8644	21.2796	21.4238
		Std	3.00E-01	2.83E-01	5.78E-01	3.35E-01	1.01E+00	1.07E+00	4.71E-01	4.13E-01	2.97E-01	**2.63E-01**
	8	Ave	23.2480	23.4539	23.1387	23.4738	2.06E+01	21.7837	23.1897	22.5259	23.3115	**23.5111**
		Std	5.33E-01	4.67E-01	6.07E-01	5.25E-01	1.41E+00	1.02E+00	5.38E-01	5.92E-01	4.61E-01	**4.42E-01**
	10	Ave	24.6356	24.9678	24.7333	24.9884	2.27E+01	22.5517	24.5022	24.1378	24.6413	**25.1029**
		Std	6.27E-01	5.54E-01	6.95E-01	**4.24E-01**	1.31E+00	9.30E-01	7.29E-01	5.42E-01	4.60E-01	6.77E-01
brain	4	Ave	25.0638	25.0848	25.0671	25.0257	2.36E+01	24.3856	25.0703	**25.1653**	25.0804	25.0864
		Std	8.76E-02	7.25E-02	1.35E-01	1.56E-01	1.00E+00	6.25E-01	7.23E-02	1.07E-01	8.02E-02	**6.84E-02**
	6	Ave	27.3888	27.4452	27.2552	27.2648	2.55E+01	25.9379	27.3864	**27.5187**	27.3323	27.3570
		Std	2.48E-01	2.58E-01	3.43E-01	3.99E-01	8.63E-01	7.60E-01	**1.79E-01**	2.60E-01	2.80E-01	2.86E-01
	8	Ave	29.0796	**29.2074**	28.8699	28.9303	2.69E+01	27.3215	29.1153	28.9077	29.1952	28.8279
		Std	2.72E-01	3.07E-01	4.36E-01	3.85E-01	6.30E-01	6.83E-01	3.90E-01	3.13E-01	4.15E-01	**2.61E-01**
	10	Ave	30.2201	**30.4718**	30.1744	30.3318	2.79E+01	28.1993	30.1926	30.0744	30.3784	30.1335
		Std	3.97E-01	3.90E-01	6.34E-01	5.29E-01	7.55E-01	7.16E-01	4.92E-01	4.41E-01	4.29E-01	**3.48E-01**
camera	4	Ave	18.9298	19.1203	18.7813	18.6073	1.77E+01	18.1498	18.5009	18.1488	**19.5198**	19.3303
		Std	9.05E-01	9.80E-01	9.16E-01	8.90E-01	1.18E+00	7.94E-01	9.02E-01	**5.92E-01**	6.10E-01	7.98E-01
	6	Ave	**21.7799**	21.5989	21.2798	21.6398	1.95E+01	19.9039	21.5264	20.7025	21.5976	21.7040
		Std	3.54E-01	7.98E-01	7.78E-01	5.95E-01	1.37E+00	1.42E+00	6.56E-01	1.26E+00	5.41E-01	**3.46E-01**
	8	Ave	23.1158	23.0478	22.8471	22.7733	2.10E+01	21.4784	22.9956	22.5575	22.9904	**23.1650**

		Std	4.82E-01	5.34E-01	6.69E-01	5.69E-01	1.50E+00	1.43E+00	5.34E-01	5.37E-01	6.06E-01	**3.08E-01**
	10	Ave	23.9441	23.8624	23.7947	23.8671	2.23E+01	22.7052	23.9184	23.3699	24.2454	**24.2781**
		Std	5.79E-01	5.81E-01	7.42E-01	8.36E-01	1.78E+00	1.40E+00	9.19E-01	**4.19E-01**	7.97E-01	8.65E-01
face	4	Ave	19.7285	19.7284	19.6050	19.6574	1.77E+01	18.9315	19.7430	19.5968	**19.7698**	19.7456
		Std	5.72E-02	1.07E-01	2.00E-01	3.19E-01	7.78E-01	7.27E-01	5.77E-02	1.63E-01	7.19E-02	**4.66E-02**
	6	Ave	22.5077	22.5480	22.3199	22.4533	2.03E+01	21.0685	22.5344	22.3241	**22.5722**	22.5479
		Std	**1.75E-01**	1.89E-01	3.54E-01	3.32E-01	7.28E-01	7.15E-01	2.56E-01	2.97E-01	2.37E-01	1.94E-01
	8	Ave	24.5753	24.6139	24.1830	24.5438	2.17E+01	22.2590	24.4826	24.3517	**24.6919**	24.3333
		Std	4.36E-01	3.48E-01	5.01E-01	3.12E-01	7.55E-01	9.91E-01	4.72E-01	4.98E-01	3.52E-01	**1.91E-01**
	10	Ave	26.0320	**26.2787**	25.4943	25.9579	2.34E+01	23.5428	25.9410	25.7531	26.1505	25.7378
		Std	3.21E-01	3.47E-01	4.67E-01	5.83E-01	1.04E+00	7.78E-01	4.70E-01	5.20E-01	**3.17E-01**	5.41E-01
girl	4	Ave	21.9776	21.9890	21.9656	21.9693	1.94E+01	21.0612	21.9993	**22.1642**	21.9886	21.9877
		Std	1.56E-01	8.85E-02	1.63E-01	3.55E-01	1.41E+00	9.20E-01	9.29E-02	1.81E-01	7.79E-02	**5.62E-02**
	6	Ave	24.3456	24.3957	24.2740	24.1105	2.16E+01	22.7919	24.2964	24.5748	**24.5866**	24.3674
		Std	2.36E-01	2.55E-01	3.85E-01	6.55E-01	1.39E+00	9.38E-01	4.90E-01	2.63E-01	2.26E-01	**2.16E-01**
	8	Ave	26.0854	26.1990	26.1704	26.1805	2.31E+01	23.6353	26.2587	26.2421	26.4768	**26.4853**
		Std	**3.32E-01**	4.40E-01	3.55E-01	3.40E-01	1.37E+00	1.81E+00	3.42E-01	3.88E-01	3.63E-01	3.66E-01
	10	Ave	27.4423	27.7294	27.5241	27.6736	2.39E+01	24.4203	27.5960	27.7087	**27.9347**	27.4999
		Std	4.53E-01	4.80E-01	5.79E-01	3.61E-01	1.63E+00	1.29E+00	4.92E-01	4.37E-01	**3.05E-01**	4.32E-01
hunter	4	Ave	21.9816	21.9625	21.9277	21.9235	2.01E+01	21.0379	21.9823	21.9526	21.9839	**21.9871**
		Std	5.98E-02	5.51E-02	1.25E-01	1.64E-01	8.83E-01	6.80E-01	4.10E-02	7.50E-02	3.87E-02	**3.57E-02**
	6	Ave	24.4189	24.5552	24.4040	24.5549	2.19E+01	22.8908	24.5608	24.5739	24.5112	**24.7046**
		Std	2.70E-01	2.27E-01	3.27E-01	2.37E-01	9.73E-01	6.42E-01	2.20E-01	2.04E-01	2.32E-01	**6.69E-02**
	8	Ave	26.0199	26.1514	26.0357	26.0073	2.35E+01	23.6181	26.1001	26.1349	25.9855	**26.2698**
		Std	2.38E-01	2.49E-01	3.48E-01	3.16E-01	8.13E-01	6.38E-01	3.34E-01	1.84E-01	**1.40E-01**	3.28E-01
	10	Ave	27.2268	27.5171	27.4490	27.3693	2.42E+01	24.9590	27.3669	27.5026	27.0834	**27.7084**
		Std	2.64E-01	3.22E-01	3.83E-01	3.93E-01	9.33E-01	8.42E-01	5.30E-01	3.15E-01	3.60E-01	**2.48E-01**
peppers	4	Ave	20.4516	20.3603	20.3403	20.3901	1.82E+01	19.3131	20.2748	20.2522	20.4502	**20.4524**
		Std	4.55E-02	2.24E-01	2.04E-01	1.17E-01	9.74E-01	6.47E-01	2.91E-01	2.80E-01	2.56E-02	**1.60E-02**
	6	Ave	23.0715	23.1400	22.9675	22.9108	2.06E+01	21.3891	23.1532	23.0751	**23.1974**	23.1184
		Std	1.39E-01	1.54E-01	2.28E-01	3.13E-01	1.04E+00	6.14E-01	1.14E-01	1.76E-01	1.83E-01	**9.16E-02**
	8	Ave	24.6590	24.7827	24.5161	24.6497	2.20E+01	22.4559	24.7579	24.6958	24.6310	**24.7968**
		Std	1.92E-01	2.31E-01	2.83E-01	2.34E-01	1.03E+00	9.78E-01	2.26E-01	2.66E-01	3.33E-01	**1.74E-01**
	10	Ave	26.0244	26.2035	25.6435	26.1071	2.31E+01	23.4122	25.8353	25.8539	**26.2312**	25.8894
		Std	**2.89E-01**	3.38E-01	3.15E-01	3.74E-01	1.41E+00	1.06E+00	4.20E-01	3.35E-01	3.12E-01	3.05E-01
saturn	4	Ave	22.3282	22.3464	22.2975	22.3010	2.13E+01	21.7383	22.3462	**22.3880**	22.3352	22.3404
		Std	5.58E-02	2.81E-02	1.56E-01	1.80E-01	6.74E-01	5.38E-01	3.34E-02	6.51E-02	4.14E-02	**2.05E-02**
	6	Ave	25.2506	25.2662	25.0547	25.2058	2.38E+01	23.9815	25.1796	**25.3799**	25.3139	25.1885
		Std	1.23E-01	1.88E-01	3.38E-01	3.21E-01	6.53E-01	5.94E-01	3.04E-01	**9.07E-02**	1.27E-01	1.49E-01
	8	Ave	27.1651	27.3364	26.9990	27.2416	2.51E+01	25.3691	27.1294	27.2920	**27.4351**	26.9750
		Std	2.41E-01	2.34E-01	4.79E-01	2.99E-01	7.31E-01	6.46E-01	3.67E-01	2.84E-01	3.14E-01	**1.79E-01**
	10	Ave	28.6316	28.8595	28.5153	28.8541	2.65E+01	26.6278	28.6532	28.8500	28.2007	**28.8854**
		Std	2.50E-01	**1.96E-01**	5.11E-01	3.07E-01	8.76E-01	6.51E-01	4.08E-01	2.95E-01	2.70E-01	3.40E-01

terrace	4	Ave	21.4676	21.4769	21.4540	21.4517	1.98E+01	20.7383	21.4751	21.4682	21.4775	**21.4800**
		Std	1.85E-02	1.09E-02	6.48E-02	7.79E-02	6.34E-01	5.38E-01	1.29E-02	3.59E-02	8.38E-03	**6.84E-03**
	6	Ave	23.8851	23.9220	23.8280	23.8868	2.17E+01	22.3114	23.9341	23.8665	23.9186	**24.0105**
		Std	1.18E-01	1.56E-01	1.77E-01	2.37E-01	6.97E-01	7.79E-01	1.77E-01	1.91E-01	1.67E-01	**3.95E-02**
	8	Ave	25.5502	25.7507	25.5102	25.6281	2.32E+01	23.4243	25.5915	25.6611	25.5058	**25.8469**
		Std	1.79E-01	2.11E-01	4.23E-01	2.64E-01	6.53E-01	7.17E-01	2.98E-01	2.41E-01	3.10E-01	**1.03E-01**
	10	Ave	26.8775	27.0616	26.8592	27.0747	2.44E+01	24.7990	26.8052	26.9382	26.5911	**27.2994**
		Std	2.72E-01	3.28E-01	4.42E-01	2.67E-01	7.77E-01	6.87E-01	4.39E-01	2.91E-01	2.43E-01	**1.86E-01**
Friedman-Rank			5.28	3.82	5.28	4.32	9.73	9.26	4.62	4.63	5.27	**2.83**
Final-Rank			7	2	8	3	10	9	4	5	6	**1**
